# Enantioselective assembly of tertiary stereocenters *via* multicomponent chemoselective cross-coupling of geminal chloro(iodo)alkanes†
†Electronic supplementary information (ESI) available. See DOI: 10.1039/c5sc04378f


**DOI:** 10.1039/c5sc04378f

**Published:** 2016-01-06

**Authors:** X. Jiang, K. Kulbitski, G. Nisnevich, M. Gandelman

**Affiliations:** a Schulich Faculty of Chemistry , Technion-Israel Institute of Technology , Technion City , Haifa-32000 , Israel . Email: chmark@tx.technion.ac.il

## Abstract

Tertiary stereocenters represent a ubiquitous and highly important motif in many pharmaceutically active compounds and natural products. Development of efficient and straightforward approaches to their enantioselective construction from readily available simple substrates is an important yet challenging goal for synthetic chemistry. Herein we describe an efficient, versatile and facile method for the highly enantioselective construction of tertiary stereocenters *via* unprecedented consecutive one-pot Suzuki reactions of non-activated racemic 1-chloro-1-iodoalkanes with alkylboranes. It represents the first cross-coupling approach which employs simple and readily available primary alkyl substrates for the direct multicomponent assembly of enantioenriched tertiary stereocenters. A simple and effective preparation of 1-chloro-1-iodoalkanes from ubiquitous α-chloroalkanoic acids is also described. Collectively, the developed methods open a door to efficient catalytic enantioselective synthesis of alkanes bearing tertiary stereocenters from carboxylic acids just in few steps.

## Abstract 2

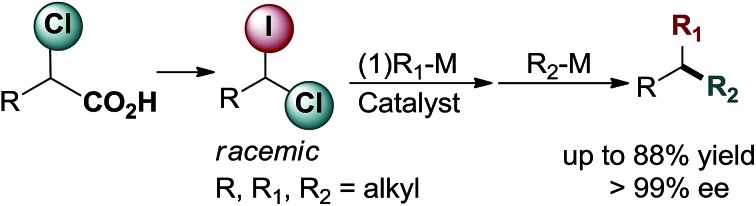
Catalytic enantioselective method of consecutive Suzuki–Miyaura alkylations of *gem*-chloro(iodo)alkanes to form two C–C bonds in one pot transformation is described.

## Introduction

Metal catalyzed cross-couplings are extremely useful and efficient reactions for construction of carbon–carbon bonds.^1^ Contrary to C_sp^2^_-based electrophiles, coupling of C_sp^3^_-based partners has proven to be a daunting challenge due to the relatively slow oxidative addition of alkyl halides and the undesirable β-hydride elimination that alkyl halides might undergo.^2^ Development of efficient catalysts for alkyl–alkyl couplings of primary and secondary alkyl electrophiles has emerged as an important frontier with a potential to significantly extend the synthetic power of the cross-coupling transformations.^3^ In this context, utilization of secondary alkyl electrophiles (or nucleophiles) in cross-couplings is especially attractive as it opens a door to the catalytic asymmetric synthesis of stereogenic tertiary centers.3d–e,^4^ All-carbon tertiary stereocenters represent an ubiquitous and highly important motif in many pharmaceutically active compounds and natural products, and the development of efficient and straightforward approaches to their construction is an important goal for organic chemistry.^5^

**Scheme 1:**
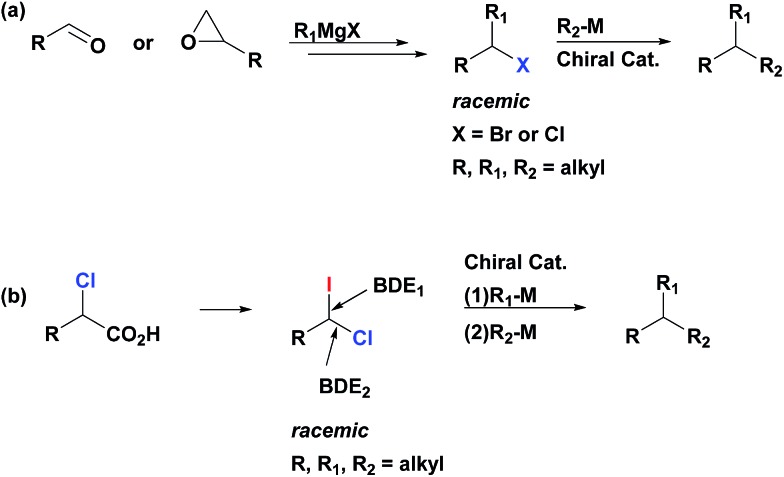
Cross-coupling approaches for stereoconvergent construction of tertiary stereocenters (a) established method employing secondary partners; (b) one-pot sequential cross-couplings of geminal 1-chloro-1-iodoalkanes with primary alkylborons (this work).

There are two major approaches that utilize cross-coupling reactions of non-activated alkyl partners for the synthesis of enantioenriched products with tertiary stereocenters.^4^–^6^ The first stereospecific approach depends on substrate control (electrophile or nucleophile) as the source of stereochemical information.4*a* This process is mediated by achiral catalysts, and requires use of the enantioenriched secondary electrophiles or nucleophiles in the stoichiometric amount. The second approach employs racemic secondary electrophiles (or nucleophiles) as starting materials, and the stereoselectivity of the product is controlled by substoichiometric amounts of the chiral catalyst (Scheme 1a).4*a* Recently, remarkable catalyst-controlled enantioselective cross-couplings have been developed for a number of families of non-activated secondary alkyl halides.^7^ It was demonstrated that this process is stereoconvergent as both stereoisomers of the racemic alkyl halides were converted into a single stereoisomer of product as the reaction proceeds through a common achiral intermediate.7*c* Notably, all reported cross-coupling processes for the preparation of tertiary stereocenters utilize secondary alkyl partners as starting materials.4*a* However, synthesis of non-symmetrical secondary alkyl halides (or, alternatively, alkylmetal reagents) might be multistep and tedious, typically involving reaction of Grignard reagents (functional group sensitive) with carbonyls or epoxides. Therefore, alternative approaches which rely on new easily available substrates for stereoselective construction of tertiary centers *via* cross-coupling reactions could significantly extend the potential of this method in terms of applicability and functional group compatibility.

## Results and discussion

Herein we describe a novel multicomponent method for the facile and highly enantioselective construction of tertiary stereocenters *via* Suzuki reactions of non-activated racemic geminal dihaloalkanes with primary alkylboranes. This represents the first example of cross-coupling that employs simple primary alkyl electrophiles and nucleophiles for the assembly of tertiary centers (Scheme 1b). We demonstrate the unprecedented use of geminal chloro(iodo)alkanes in cross-coupling reactions, in which the chemoselective functionalization of the C–I bond (bond dissociation energy (BDE_1_) = 57 kcal mol^–1^)^8^ with the first alkylborane, followed by functionalization of the stronger C–Cl bond (BDE_2_ = 84 kcal mol^–1^)^8^ with a second alkylborane in a consecutive step resulting in a one-pot transformation. Importantly, the same catalyst system and reaction conditions are used for both steps of this stereoconvergent transformation. Moreover, we disclose a general and efficient approach to access 1-chloro-1-iodoalkanes. This chemistry relies on the facile iododecarboxylation of α-chloroalkanoic acids, which, in turn, are easily prepared by α-chlorination of the corresponding alkanoic acids.^9^ Collectively these methods constitute a short, straightforward and efficient approach for the construction of highly enantioenriched alkanes bearing tertiary stereocenters (up to 99% ee) from abundant primary alkanoic acids.

**Scheme 2:**
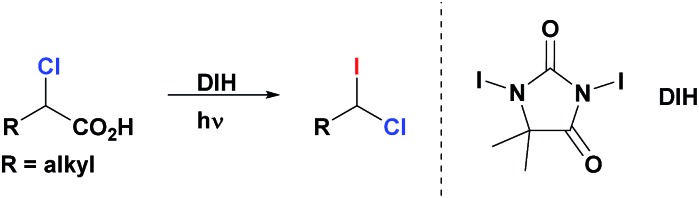
Synthesis of geminal chloro(iodo)alkanes *via* iododecarboxylation of α-chloroalkanoic acids.

1-Chloro-1-iodoalkanes constitute an attractive class of polyfunctional compounds. However, the development of their chemistry has been an uneven process, most likely, due to the lack of selective, practical and high yielding methods of their synthesis.^10^ Recently, we reported a robust, general and efficient method for synthesis of alkyl iodides from carboxylic acids through their simple treatment with commercially available 1,3-diiodo-5,5-dimethylhydantoin (DIH) under visible light (VIS) irradiation.^11^ We now report that, contrary to other iododecarboxylation approaches,10*d* our method can be uniquely extended to α-chloroalkanoic acids. Thus, these compounds react smoothly with DIH under VIS-irradiation to generate the corresponding 1-chloro-1-iodoalkanes (Scheme 2). These products are isolated in an essentially pure form after a simple aqueous work-up of the reaction mixture (see ESI†). All 1-chloro-1-iodoalkanes presented in this paper (Tables 1, 3 and 4) were prepared and isolated by this method, starting from the corresponding α-chloroalkanoic acids, in a selective manner and high yields.

**Table 1:** Investigation of the double alkyl–alkyl Suzuki cross-coupling reaction^*a*^

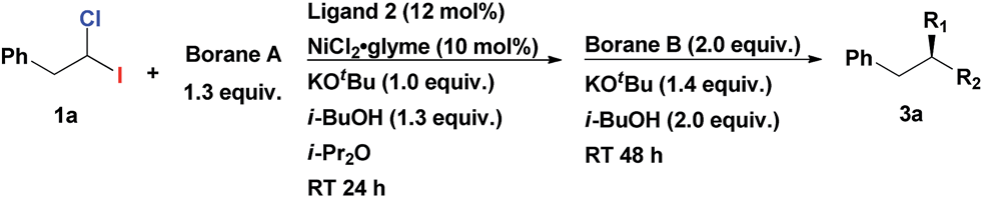					
Entry	Borane A	Borane B	R_1_	R_2_	Yield^*b*^, ee (%)
1	Pr_3_B	Ph(CH_2_)_3_-9-BBN	Pr	Ph(CH_2_)_3_	73, 86
2	Pr-9-BBN	Ph(CH_2_)_3_-9-BBN	Pr	Ph(CH_2_)_3_	84, 96
3	Ph(CH_2_)_3_-9-BBN	Pr-9-BBN	Ph(CH_2_)_3_	Pr	70, 82
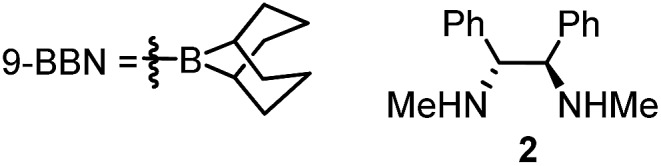					

^*a*^Reactions were conducted with **1a** (0.2 mmol), NiCl_2_·glyme (0.02 mmol), ligand (0.024 mmol), borane A (0.26 mmol), KO^*t*^Bu (0.2 mmol), i-BuOH (0.26 mmol) in i-Pr_2_O (2 mL) at RT. After 24 h, borane B (0.4 mmol), KO^*t*^Bu (0.28 mmol), i-BuOH (0.4 mmol) were added and adjusted the total volume to 4 mL with i-Pr_2_O.

^*b*^Isolated yields.

Having an efficient synthetic access to these synthons in hand, we examined their applicability in the construction of tertiary stereocenters through a direct multicomponent reaction. Based on the substantial difference in the BDE's of C–I and C–Cl bonds, we hypothesized that it might be possible to selectively activate the C–I bond in a cross-coupling reaction with alkylmetal R_1_–M, following by functionalization of the C–Cl bond in a second cross-coupling with a different alkylmetal R_2_–M (see Scheme 1b). Ideally, one catalyst system might be able to mediate both steps. Since the starting material, 1-chloro-1-iodoalkane, is racemic, a stereoconvergent synthesis of enantiomerically enriched products with tertiary chiral centers may be envisioned if an appropriate chiral catalyst could be identified.

Initiating an active program on studies of this general concept of multicomponent assembly of the tertiary chiral centers, we started from investigating homobenzylic geminal chloroiodides in the asymmetric Suzuki alkyl–alkyl coupling reactions. Recently, Fu *et al.* reported the first asymmetric Suzuki cross-coupling of secondary homobenzylic bromides with alkyl-9-BBN.7*a* In this seminal work, it was demonstrated that ArCH_2_-group in electrophile is necessary for ensuring good enantioselectivity, most likely, due to its weak secondary interaction with the catalyst. We firstly investigated the reaction of homobenzylic dihalide substrate **1a** in the reaction with different alkylborane agents in the presence of NiCl_2_·glyme and diamine ligand **2** (Table 1). Gratifyingly, subjecting **1a** to the coupling conditions with tripropylborane, following by addition of Ph(CH_2_)_3_-9-BBN to the reaction mixture (after complete conversion of **1a**), resulted in formation of the tertiary asymmetric alkane **3a** which was isolated in 73% yield and 86% ee (Table 1, entry 1). Remarkably, the same catalyst system is operative in both steps of the transformation. The use of alkyl-9-BBN as the nucleophile source in both stages of the reaction proved to be superior to other tested boron reagents. The replacement of Pr_3_B with Pr-9-BBN furnished the product in even higher yield and enantioselectivity (84% yield, 96% ee; entry 2). Inversion of the addition sequence, *i.e.* first adding the bulkier Ph(CH_2_)_3_-9-BBN in the first step following by the less bulky Pr-9-BBN, proved to be less efficient (entry 3).

Any deviation from these reaction conditions resulted in inferior results (Table 2). We tested the chiral secondary diamine ligands **4–9** in the multicomponent cross-coupling of **1a** with Pr-9-BBN and Ph(CH_2_)_3_-9-BBN; however, none of these outperformed the original ligand **2** (compare entry 1 with entries 2–7). Reaction using ligand **6**, bearing electron rich aryl substituents on the ethylenediamine backbone, provided **3a** with enantioselectivity comparable to ligand **2** (94% ee, entry 4). However analogs of **6** with electron poor aryls or bulky naphthyl substituents gave the product in significantly lower ee (entries 2, 3 and 5).

**Table 2:** Optimization of reaction conditions^*a*^

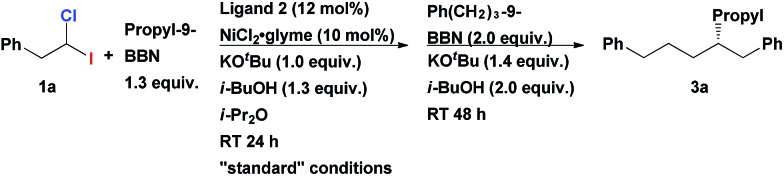		
Entry	Variation from the “standard” conditions	Yield^*b*^, ee (%)
1		84, 96
2	**4** as ligand instead of **2**	79, 86
3	**5** as ligand instead of **2**	71, 73
4	**6** as ligand instead of **2**	80, 94
5	**7** as ligand instead of **2**	82, 68
6	**8** as ligand instead of **2**	65, 90
7	**9** as ligand instead of **2**	67, 78
8	NiBr_2_·diglyme instead of NiCl_2_·glyme	77, 89
9	Ni(cod)_2_ instead of NiCl_2_·glyme	68, 87
10	Hexanol instead of i-BuOH	80, 92
11	Et_2_O instead of i-Pr_2_O	81, 96
12	Dioxane instead of i-Pr_2_O	75, 80
13	6% cat. and 8% ligand instead of 10%/12%	61, 84
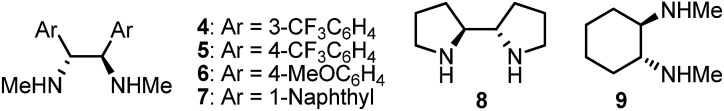		

^*a*^Reactions were conducted with **1a** (0.2 mmol), Ni salt (0.02 mmol), ligand (0.024 mmol), propyl-9-BBN (0.26 mmol), KO^*t*^Bu (0.2 mmol), i-BuOH or hexanol (0.26 mmol) in i-Pr_2_O or dioxane (2 mL) RT. After 24 h, Ph(CH_2_)_3_-9-BBN (0.4 mmol), KO^*t*^Bu (0.28 mmol), i-BuOH or hexanol (0.4 mmol) were added and adjusted the total volume to 4 mL with the corresponding solvent.

^*b*^Isolated yield.

Interestingly, bis-pyrrolidine ligand **8**, which was recently used by us for the efficient synthesis of chiral secondary fluoroalkanes *via* Suzuki cross-coupling of 1-bromo-1-fluoroalkanes,^12^ also afforded a product with high enantioselectivity, albeit with considerably lower yield (entry 6). Utilizing other sources of Ni(ii) or Ni(0) instead of NiCl_2_·glyme, as well as lower loadings of Ni salt and ligand, eroded the reaction efficiency (entries 8, 9 and 13). While dioxane is unsuitable as a solvent (entry 12), diethyl ether proved to be as efficient as diisopropyl ether (entry 11). We preferred to use the higher boiling diisopropyl ether in order to ensure a constant concentration of the reaction partners in the course of the process.

Having arrived at the optimal reaction conditions, we explored the substrates scope of this multicomponent coupling (Table 3 and 4). The transformation is very versatile as the R, R_1_ and R_2_ fragments can be easily varied upon assembly of tertiary center; therefore, diverse combinations can be envisioned (see Scheme 1b). We found that the introduction of a relatively compact alkyl group in the first step ensures overall high enantioselectivity (*vide supra*; see Table 1).

**Table 3:** Scope of R_1_-9-BBN^*a*^

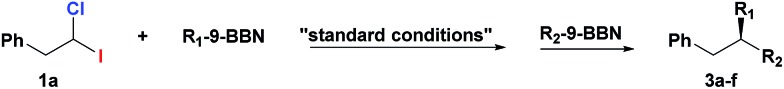				
Entry	R_1_	R_2_	Product	Yield^*b*^, ee (%)
1	Et	Ph(CH_2_)_3_	**3b**	79, 92
2	Pr	Ph(CH_2_)_3_	**3a**	85, 96
3	Bu	Ph(CH_2_)_3_	**3c**	80, 98
4	Pentyl	Ph(CH_2_)_3_	**3d**	81, 80
5	Hexyl	Ph(CH_2_)_3_	**3e**	83, 89
6	Ph(CH_2_)_3_	4-MeOC_6_H_4_(CH_2_)_3_	**3f**	68, 78

^*a*^Reactions were conducted with **1a** (0.5 mmol), Ni salt (0.05 mmol), ligand 2 (0.06 mmol), R_1_-9-BBN (0.65 mmol), KO^*t*^Bu (0.5 mmol), i-BuOH (0.65 mmol) in i-Pr_2_O 5 mL at RT. After 24 h, R_2_-9-BBN (1.0 mmol), KO^*t*^Bu (0.7 mmol), i-BuOH (1.0 mmol) were added and adjusted the total volume to 10 mL with i-Pr_2_O.

^*b*^Isolated yields.

**Table 4:** Scope of 2-aryl-*gem*-chloro(iodo)ethanes and R-9-BBN^*a*^

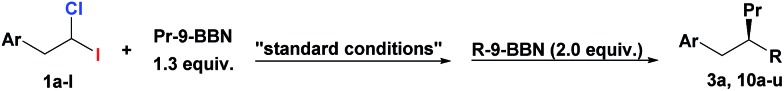					
Entry	Ar	Substrate	R	Product	Yield^*b*^, ee (%)
1	Ph	**1a**	Ph(CH_2_)_3_	**3a**	85, 96
2	Ph	**1a**	4-FC_6_H_4_(CH_2_)_3_	**10a**	81, 92
3	Ph	**1a**	4-MeC_6_H_4_(CH_2_)_3_	**10b**	85, 87
4	Ph	**1a**	4-MeOC_6_H_4_(CH_2_)_3_	**10c**	88, 82
5	Ph	**1a**	Ph(CH_2_)_4_	**10d**	81, 78
6	4-MeOC_6_H_4_	**1b**	Ph(CH_2_)_3_	**10e**	82, >99.5
7	4-MeOC_6_H_4_	**1b**	2-MeOC_6_H_4_(CH_2_)_3_	**10f**	88, 99.5
8	4-MeOC_6_H_4_	**1b**	4-MeOC_6_H_4_(CH_2_)_3_	**10g**	87, 94
9	4-MeOC_6_H_4_	**1b**	4-CF_3_C_6_H_4_(CH_2_)_3_	**10h**	82, 95
10	4-MeOC_6_H_4_	**1b**	4-FC_6_H_4_(CH_2_)_3_	**10i**	85, 94
11	4-MeOC_6_H_4_	**1b**	4-MeC_6_H_4_(CH_2_)_3_	**10j**	86, 92
12	4-MeOC_6_H_4_	**1b**	TBSO(CH_2_)_3_	**10k**	80, 81
13	4-MeOC_6_H_4_	**1b**	Cyclohexyl(CH_2_)_3_	**10l**	84, 78
14	2-MeC_6_H_4_	**1c**	Ph(CH_2_)_3_	**10m**	78, 87
15	3-MeC_6_H_4_	**1d**	TBSO(CH_2_)_3_	**10n**	84, 80
16	4-MeC_6_H_4_	**1e**	Ph(CH_2_)_3_	**10o**	86, 78
17	2-FC_6_H_4_	**1f**	Ph(CH_2_)_3_	**10p**	83, 72
18	3-FC_6_H_4_	**1g**	Ph(CH_2_)_3_	**10q**	80, 82
19	4-FC_6_H_4_	**1h**	TBSO(CH_2_)_3_	**10r**	79, 38
20	4-CF_3_C_6_H_4_	**1i**	Ph(CH_2_)_3_	**10s**	81, 89
21	2-Napthyl	**1j**	Ph(CH_2_)_3_	**10t**	58, 71
22	3-MeOC_6_H_4_	**1k**	Ph(CH_2_)_3_	**10u**	78, 88

^*a*^Reactions were conducted with 1 (0.5 mmol), Ni salt (0.05 mmol), ligand 2 (0.06 mmol), *n*-propyl-9-BBN (0.65 mmol), KO^*t*^Bu (0.5 mmol), i-BuOH (0.65 mmol) in i-Pr_2_O (5 mL) at RT. After 24 h, R-9-BBN (1.0 mmol), KO^*t*^Bu (0.7 mmol), i-BuOH (1.0 mmol) were added and adjusted the total volume to 10 mL with i-Pr_2_O.

^*b*^Isolated yields.

As such, several unbulky alkyl-9-BBN were initially examined as *trans*-metallation partners in the first step (Table 3). Although a very good to high enantioselectivity is obtained in each case, the variation of the chain length in R_1_ group has a distinguishable influence. While the shorter ethyl, propyl and butyl motifs ensure highly enantioenriched products (entries 1–3), the longer pentyl, hexyl and 3-phenylpropyl units lead to slightly diminished ee (entries 4–6).

Selecting propyl-9-BBN as a representative nucleophile for the first cross-coupling event, we demonstrated the flexibility of the reaction with respect to the alkylborane, utilized in the second step (R_2_-9-BBN), and 2-aryl-1-chloro-1-iodoethanes (ArCH_2_CHClI; **1a–k**). These results are summarized in Table 4. A wide array of non-activated homoaryl geminal chloroiodides was tolerated in the reaction. Both phenyl as well as *ortho*-, *meta*- and *para*-substituted phenyls with both electron donating and withdrawing groups participated in the reaction affording the products in high yields and enantioselectivities with variety of organoboron partners. Interestingly, having an electron donating methoxy-group in the *para*-position of the aromatic ring in homoaryl dihalide (*i.e.* compound **1b**) was beneficial providing **10e–10l** in enantioselectivities up to 99% ee (entries 6–13).

An increase of electron density in the aromatic ring possibly strengthens its secondary interaction with the catalyst in the enantiodiscriminative step. Indeed, 1-chloro-1-fluoro-2-(*p*-fluorophenyl)ethane (**1h**), bearing a strongly withdrawing fluoride substituent in *para*-position, reacts with considerably lower enantioselectivity (compare entries 6 *vs.* 20; 12 *vs.* 19). 1-Chloro-1-iodo-2-(*meta*-methoxyphenyl)ethane (**1k**) leads to the tertiary alkane **10u** with lower optical purity (88% ee) than the analogous *para*-substituted substrate **1b** (compare entries 6 and 22). It is conceivable that the secondary interactions between the aromatic system and the catalyst are relatively weak in case of the substrate **1k** due to steric congestion imposed by the *meta*-methoxy substituent. Noteworthy, the high yields and enantioselectivities are obtained in these reactions under the mild conditions and at room temperature.

**Scheme 3:**
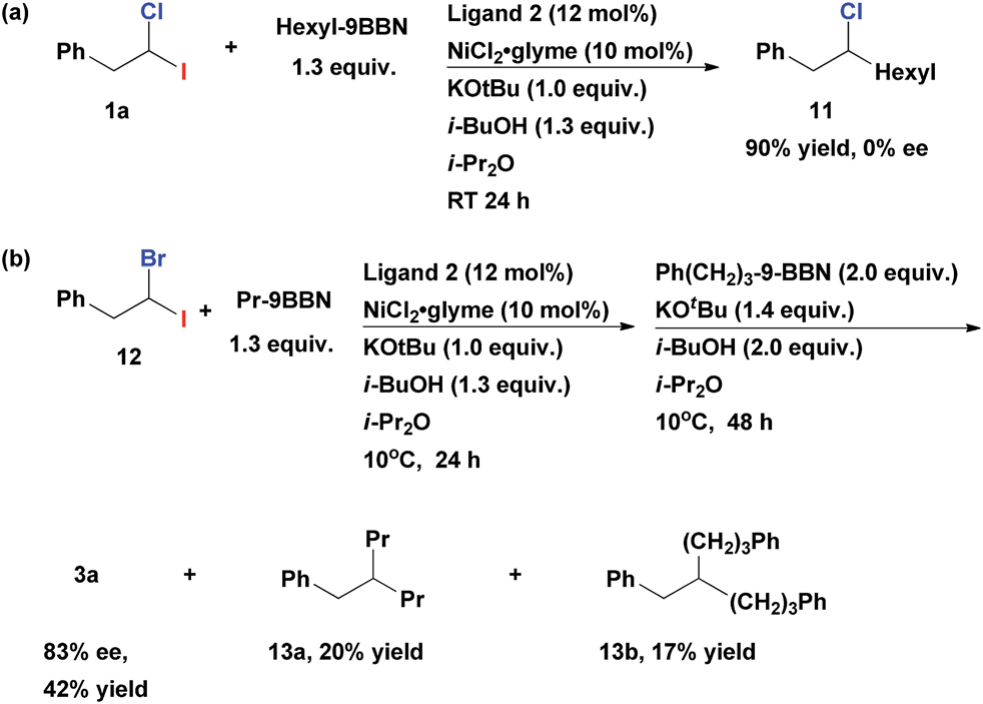
(a) Generation of the racemic intermediate **11** (b) enantioselective alkyl–alkyl Suzuki cross-coupling of geminal bromo-iodoalkane **12**.

When *gem*-dihaloalkane **1a** was cross-coupled with hexyl-9-BBN under the standard reaction conditions, the corresponding unsymmetrical secondary alkyl chloride **11** was isolated in 90% yield (Scheme 3a). However, the product was a racemic mixture of enantiomers. A complete absence of ee in **11** was surprising, since when such cross-coupling was performed with the corresponding fluoro(halo)alkanes as substrates the resulting secondary alkyl fluorides were isolated in highly enantioenriched form.^12^ This difference can be explained by the assumption that the C–Cl bond in secondary alkyl chlorides (*e.g.*, **11**) undergoes fast reversible oxidative addition/reductive elimination process under our cross-coupling conditions which leads to epimerization. This process is apparently impossible in case of stronger C–F bond. Since the first coupling step leads to racemic alkyl chlorides, we can conclude that the second cross-coupling event represents an enantiodiscriminative step under the given developed reaction conditions.^13^

It should be mentioned, that the developed multicomponent transformation is efficient and chemoselective only for geminal alkanes possessing a combination of chloro- and iodo-substituents. Geminal homo-dihaloalkanes are not suitable substrates for this method. It was previously reported, that CH_2_Cl_2_ undergoes dialkylation with BuMgBr *via* Kumada cross-couplings to result in nonane even if a large excess of dichloromethane is used and reaction is carried out at –20 °C.^14^ Similarly, reaction of 1,1-diiodo-2-phenylethane under our coupling conditions mainly resulted in double alkylation already in the first step.

When 1-bromo-1-iodo-2-phenylethane **12** was tested in the representative multicomponent cross-coupling, the asymmetric alkane **3a** was obtained in 42% yield and 83% ee (Scheme 3b). However, the main side products were doubly alkylated compounds **13a** and **13b** even if this reaction is performed at 10 °C. As such, the obtained exclusive chemoselectivity (in the first step) in our method is quite surprising, given that both coupling steps are mediated with the same catalyst under the same reaction conditions.

## Conclusions

In conclusion, we have developed an efficient, versatile and facile method for the highly enantioselective construction of tertiary stereocenters through an unprecedented Suzuki reactions of non-activated racemic geminal chloro(iodo)-alkanes with alkylboranes. We have established the first cross-coupling approach which employs simple and readily available primary alkyl substrates for the direct multicomponent assembly of enantioenriched tertiary stereocenters. The ability to selectively functionalize an alkyl C–I bond in the presence of geminal C–Cl bond is an important factor for the success of this one-pot two-step transformation. Importantly, the same catalyst system is used for both steps of this stereoconvergent transformation, and the selectivity is achieved under mild conditions and at room temperature. In addition, we have disclosed a general and efficient preparation of 1-chloro-1-iodoalkanes, the electrophiles used in the described cross-coupling transformation. These highly functional compounds have become readily available due to developed direct iododecarboxylation of the corresponding ubiquitous α-chloroalkanoic acids. Collectively, the developed methods open a door to efficient catalytic enantioselective synthesis of alkanes bearing tertiary stereocenters from carboxylic acids just in few steps. Further studies on the reactivity of unactivated geminal dihaloalkanes are underway in our labs.

## Supplementary Material

Supplementary information
