# Supplementary material for: Enantioselective assembly of tertiary stereocenters via multicomponent chemoselective cross-coupling of geminal chloro(iodo)alkanes
Source: Chem Sci. 2016 Jan 6;7(4):2762–7. doi: 10.1039/c5sc04378f (PMC5477020; doi:10.1039/c5sc04378f)
Supplement: Supplementary file 1 [file SC-007-C5SC04378F-s001.pdf]

Electronic Supplementary Material (ESI) for Chemical Science.
This journal is © The Royal Society of Chemistry 2016

Enantioselective Assembly of Tertiary Stereocenters via Multicomponent Chemoselective Cross-Coupling of Geminal Chloro(iodo)alkanes

Xiaojian Jiang, Kseniya Kulbitski, Gennady Nisnevich and Mark Gandelman*

Schulich Faculty of Chemistry, Technion - Israel Institute of Technology, Technion City, Haifa 32000, Israel.

**e-mail: chmark@tx.technion.ac.il.*

GENERAL METHODS

General procedures --- 3

Materials -- 3

Instrumentation -- 3

PREPARATION OF SUBSTRATES

Representative procedure of synthesis geminal dihalide substrate -----------------------------------4

SYTHESIS OF DOUBLE CROSS-COUPLING PRODUCTS

Representative procedure for the generation of coupling product ----------------------------------- 8

Determination of absolute configuration -- 12

References --- 38

^{1}H/^{13}C/^{19}F Spectra -- 39

GENERAL METHODS

General Procedures

All reactions were generally performed in glove box or in dried glassware under an atmosphere of dry N_2. Reaction mixtures were stirred magnetically unless otherwise indicated and monitored by thin layer chromatography (TLC) on Merck precoated glass-backed silica gel 60 F-254 0,25 mm plates with visualization by fluorescence quenching at 254 nm. TLC plates were stained using potassium permanganate. Chromatography purification of products (flash column chromatography) was performed on silica gel 60 (70-230 mesh, Merck) using a forced flow of eluent at 0.3-0.5 bar. Concentration of reaction product solutions and chromatography fractions under reduced pressure was performed by rotary evaporation at 35-45^oC at the appropriate pressure and then at rt, ca. 10 mmHg (vacuum pump) unless otherwise indicated.

Materials

All chemicals, including dry solvents were purchased from Aldrich, Fluka, Acros, TCI, Merck, Strem, DiaminoPharm or Alfa Aesar and used as such unless stated otherwise. Yields given refer to chromatographically purified compounds unless otherwise demonstrated.

Instrumentation

Infrared (IR) spectra were recorded on a FTIR-Bruker spectrophotometer and reported as wavenumber (cm^{-1}) of the absorption maxima for the range between 4000 cm^{-1} and 400 cm^{-1} with only major peaks reported. ^1H NMR spectra was recorded on Bruker 500 MHz, 400 MHz, 300 MHz and 200 MHz spectrometer. ^{13}C NMR spectra was recorded on Bruker 125 MHz, 100 MHz, 75 MHz and 50 MHz spectrometer. ^{19}F NMR spectra was recorded on Bruker 188 MHz. ^1H NMR chemical shifts are expressed in parts per million (δ) downfield from tetramethylsilane (with the $CHCl_3$ peak at 7.26 ppm used as standard). ^{13}C NMR chemical shifts are expressed in parts per million (δ) downfield from tetramethylsilane (with the central peak of $CHCl_3$ at 77.16 ppm used as standard). ^{19}F NMR chemical shifts are expressed in parts per million (δ) (with the C_6F_6 peak at -164.9 ppm used as standard). All ^{13}C and ^{19}F spectra were measured with complete proton decoupling. NMR coupling constants (J) are reported in Hertz (Hz), and splitting patterns are indicated as follows: br, broad; s, singlet; d, doublet; dd, doublet of doublet; ddd, doublet of doublet of doublet; dt, doublet of triplet; t, triplet; q, quartet; m, multiplet. High resolution mass

spectrometric measurements (HRMS) were performed by the Waters LCT Premier and Bruker maxis impact with APCI solid probe. Enantiomeric excess were determined by HPLC analysis on Shimadzu HPLC units including the following instruments: pump, LC-20AT; detector, SPD-M20A; column, Daicel chiralcel OJ-H or OD-H. Optical rotations were recorded on a polarimeter (Optical Activity LTD).

PREPARATION OF SUBSTRATES

Representative procedure of synthesis geminal dihalide substrate

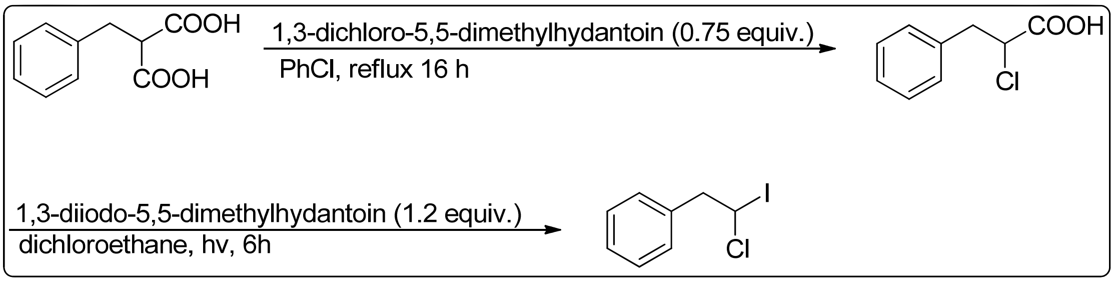

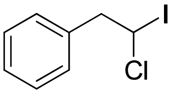

(2-chloro-2-iodoethyl)benzene

In a 100 mL round bottom flask, benzyl malonic acid (1.16 g, 6 mmol) 1,3-dichloro-5,5-dimethylhydantoin (886 mg, 4.5 mmol) and PhCl 18 mL were charged at room temperature under N_2 flow. The mixture was heated to reflux and stirred for 16 h. The reaction mixture was then cooled to room temperature. After evaporating the solvent, the mixture was directly subjected to flash column chromatography using hexanes/EtOAc as eluent and obtained the desired Chloro-acid intermediate as yellow color oil, which was subjected to iodo-decarboxylation process followed by reported procedure[1].

85%; Yellow oil; IR (neat) 3030, 3015, 699, 417; ^{1}H NMR ($CDCl_3$, 400 MHz): δ 7.30-7.22 (m, 5H), 5.79-5.76 (t, J = 13.6 Hz, 1H), 3.67-3.51 (m, 2H); ^{13}C NMR ($CDCl_3$, 100 MHz): δ 137.4, 129.4, 128.7, 128.5, 127.7, 52.8, 29.6; HRMS (APCI) calcd for C_8H_8NaClI $[M + Na]^+$: 288.9257; found: 288.9230.

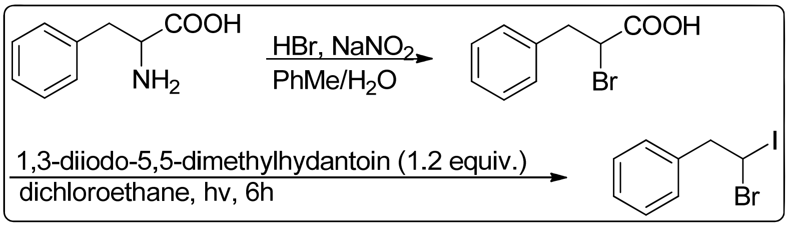

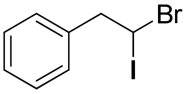

(2-bromo-2-iodoethyl)benzene

In a 100 mL round bottom flask, 48% HBr (4.08g, 24.2 mmol), water 2 mL and PhMe 6 mL were charged at 15 °C under N_2 flow. The mixture was cooled down to 0 °C and phenylalanine (1g, 6 mmol) was added. The reaction mixture was cooled to -5 °C. A solution of $NaNO_2$ (543 mg, 7.9 mmol) in water 1mL was added dropwise to the reaction mixture. After the addition, the reaction mixture was brought to room temperature and stirred for another 3 h. The organic phase was separated and diluted with 10 mL toluene which subsequently washed with water (10 mL) then brine (10 mL).The organic phase was then dried over $MgSO_4$ and evaporated to produce the desired Bromo-acid intermediate which was subjected to next step (iodo-decarboxylation) without further purification.

80%; Yellow oil; IR (neat) 3020, 3007, 698, 402; ^{1}H NMR ($CDCl_3$, 400 MHz): δ 7.31-7.19 (m, 5H), 5.53-5.50 (t, *J* = 7.2 Hz, 1H), 3.67-3.51 (m, 2H); ^{13}C NMR ($CDCl_3$, 100 MHz): δ 137.4, 129.4, 128.7, 128.5, 127.7, 52.8, 29.6; HRMS (APCI) calcd for C_8H_8NaBrI $[M + Na]^+$: 332.8752; found: 332.8750.

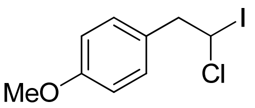

1-(2-chloro-2-iodoethyl)-4-methoxybenzene

76%; Yellow oil; IR (neat) 3031, 3015, 704, 414; ^{1}H NMR ($CDCl_3$, 400 MHz): δ 7.14-6.82 (m, 4H), 5.73-5.70 (t, *J* = 6.8 Hz, 1H), 3.77 (s, 3H), 3.60-3.43 (m, 2H); ^{13}C NMR ($CDCl_3$, 100 MHz): δ 159.1, 130.5, 129.6, 129.4, 114.0, 55.3, 51.9, 30.8; HRMS (APCI) calcd for $C_9H_{10}OClI$ $[M]^+$: 295.9465; found: 295.9467.

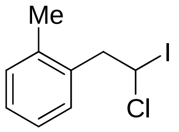

1-(2-chloro-2-iodoethyl)-2-methylbenzene

86%; Yellow oil; IR (neat) 3029, 3012, 701, 418; ^{1}H NMR ($CDCl_3$, 400 MHz): δ 7.25-7.20 (m, 4H), 5.83-5.80 (t, *J* = 7.0 Hz, 1H), 3.78-3.60 (m, 2H), 2.36 (s, 3H); ^{13}C NMR ($CDCl_3$, 100 MHz): δ 136.4, 136.0, 130.8, 130.3, 127.9, 126.3, 50.2, 28.8, 19.7.

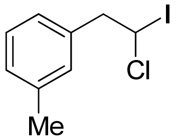

1-(2-chloro-2-iodoethyl)-3-methylbenzene

84%; Yellow oil; IR (neat) 3028, 3011, 698, 416; ^{1}H NMR ($CDCl_3$, 200 MHz): δ 7.31-7.08 (m, 4H), 5.86-5.79 (t, *J* = 6.0 Hz, 1H), 3.74-3.54 (m, 2H), 2.40 (s, 3H); ^{13}C NMR ($CDCl_3$, 50 MHz): δ 138.3, 137.3, 130.1, 128.5, 128.4, 126.4, 52.8, 29.8, 21.4; HRMS (APCI) calcd for $C_9H_{10}ClI$ $[M]^+$: 279.9516; found: 279.9456.

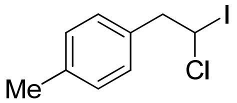

1-(2-chloro-2-iodoethyl)-4-methylbenzene

85%; Yellow oil; IR (neat) 3031, 3013, 699, 420; ^{1}H NMR ($CDCl_3$, 300 MHz): δ 7.15 (s, board, 4H), 5.80-5.76 (t, *J* = 6.9 Hz, 1H), 3.67-3.49 (m, 2H), 2.34 (s, 3H); ^{13}C NMR ($CDCl_3$, 75 MHz): δ 137.3, 134.3, 129.3, 129.2, 52.3, 30.2, 21.2.

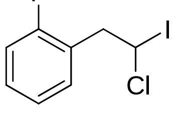

1-(2-chloro-2-iodoethyl)-2-fluorobenzene

82%; Yellow oil; IR (neat) 3024, 3012, 697, 415; ^{1}H NMR ($CDCl_3$, 300 MHz): δ 7.35-7.02 (m, 4H), 5.90-5.85 (t, J = 6.4 Hz, 1H), 3.77-3.58 (m, 2H); ^{13}C NMR ($CDCl_3$, 75 MHz): δ 131.8 (d, J = 4.2 Hz), 129.6 (d, J = 8.2 Hz), 124.1 (d, J = 21.5Hz), 115.6 (d, J = 21.5 Hz), 46.5, 27.3; ^{19}F NMR ($CDCl_3$, 188 MHz): δ -121.0.

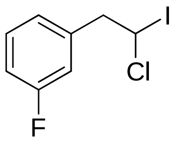

1-(2-chloro-2-iodoethyl)-3-fluorobenzene

84%; Yellow oil; IR (neat) 3027, 3014, 696, 413; ^{1}H NMR ($CDCl_3$, 200 MHz): δ 7.38-6.96 (m, 4H), 5.85-5.78 (t, J = 7.0 Hz, 1H), 3.74-3.52 (m, 2H); ^{13}C NMR ($CDCl_3$, 50 MHz): δ 130.2 (d, J = 8.2 Hz), 125.0, 116.6 (d, J = 21.4Hz), 114.9 (d, J = 20.9 Hz), 52.2, 28.2; ^{19}F NMR ($CDCl_3$, 188 MHz): δ -115.9; HRMS (APCI) calcd for $C_8H_7NaFClI$ $[M+Na]^+$: 306.9163; found: 306.9196.

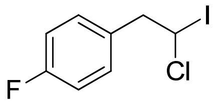

1-(2-chloro-2-iodoethyl)-4-fluorobenzene

90%; Yellow oil; IR (neat) 3020, 3011, 698, 411; ^{1}H NMR ($CDCl_3$, 200 MHz): δ 7.28-7.00 (m, 4H), 5.82-5.75 (t, J = 6.0 Hz, 1H), 3.71-3.48 (m, 2H); ^{13}C NMR ($CDCl_3$, 50 MHz): δ 131.2 (d, J = 8.1 Hz), 115.8 (d, J = 21.4 Hz), 51.7, 29.6; ^{19}F NMR ($CDCl_3$, 188 MHz): δ -117.6; HRMS (APCI) calcd for $C_8H_7NaFClI$ $[M+Na]^+$: 306.9163; found: 306.9160.

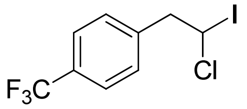

1-(2-chloro-2-iodoethyl)-4-(trifluoromethyl)benzene

91%; Yellow oil; IR (neat) 3024, 3008, 697, 409; ^{1}H NMR ($CDCl_3$, 300 MHz): δ 7.61-7.35 (m, 4H), 5.84-5.79 (t, J = 6.3 Hz, 1H), 3.76-3.59 (m, 2H); ^{13}C NMR ($CDCl_3$, 75 MHz): δ 141.0, 129.8, 125.6 (q, J = 4.1 Hz), 52.0, 27.5; ^{19}F NMR ($CDCl_3$, 188 MHz): δ -65.8; HRMS (APCI) calcd for $C_9H_9OF_3ClI$ $[M+H_2O]^+$: 351.9339; found: 351.9336.

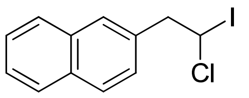

2-(2-chloro-2-iodoethyl)naphthalene

79%; Yellow oil; IR (neat) 3033, 3012, 702, 422; ^{1}H NMR ($CDCl_3$, 200 MHz): δ 7.89-7.35 (m, 7H), 5.97-5.90 (t, *J* = 6.0 Hz, 1H), 3.92-3.70 (m, 2H); ^{13}C NMR ($CDCl_3$, 50 MHz): δ 135.2, 133.4, 128.4, 128.3, 127.8, 127.1, 126.4, 126.1, 52.8, 29.5; HRMS (APCI) calcd for $C_{12}H_{10}ClI$ $[M]^+$: 315.9510; found: 315.9542.

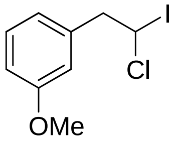

1-(2-chloro-2-iodoethyl)-3-methoxybenzene

77%; Yellow oil; IR (neat) 3025, 3014, 700, 423; ^{1}H NMR ($CDCl_3$, 300 MHz): δ 7.68-6.59 (m, 4H), 5.97-5.92 (t, *J* = 7.2 Hz, 1H), 3.85-3.58 (m, 5H); ^{13}C NMR ($CDCl_3$, 75 MHz): δ 140.0, 135.9, 130.2, 117.6, 114.5, 55.5, 50.8, 26.6; HRMS (APCI) calcd for $C_9H_{10}OClI$ $[M]^+$: 295.9465; found: 295.9467.

SYTHESIS OF DOUBLE CROSS-COUPLING PRODUCTS

Representative procedure for the generation of 3b.

1. Preparing of *n*-propyl-9BBN stock solution. In a glove box, 9-BBN dimer (1.22g, 5 mmol) *i*-Pr_2O (7 mL) was added in turn to a hydrogenation reactor. The reactor was capped and removed from the glove box. Propene gas was filled in the reactor and the reaction mixture was stirred under 5 atmospheres at room temperature (r.t.). After 16 hours, the reactor was taken back to the glove box and the reaction mixture was diluted with *i*-Pr_2O to furnish a 1 M solution. Next, a portion of the solution (2 mL, 2 mmol) was added to a solution of KO^{t}Bu (179 mg, 1.6 mmol) in *i*-BuOH (148 mg, 2 mmol) in a 10-mL vial. The resulting mixture was stirred at r.t. for at least 45 mins, extra *i*-Pr_2O was added to make the solution with 0.5 M concentration.

2. Preparing of benzenepropyl-9BBN stock solution.

In a glovebox, 9-BBN dimer (244 mg, 1 mmol), *i*-Pr_2O (2 mL), and the Allyl benzene (260 mg, 2.2 mmol) were added in turn to a 10-mL vial and stirred for 24 h at room temperature. Next, this reaction mixture was added to a solution of KO^{t}Bu (156 mg, 1.4 mmol) in *i*-BuOH (148 mg, 2.0 mmol) in a 10-mL vial. The resulting mixture was stirred at r.t. for at least 45 mins, extra *i*-Pr_2O was added to make the solution with 0.5 M concentration.

A solution of $NiCl_2$.glyme (11.0 mg, 0.050 mmol) and ligand **2** (14.4 mg, 0.06 mmol) in *i*-Pr_2O (1 mL) in a 20-mL vial was stirred at r.t. for 2 h. Next, geminal iodide-chloride electrophile **1a** (133 mg, 0.5 mmol) was added followed by 5.5 mL of *i*-Pr_2O, and then the solution of the activated *n*-propyl-9BBN (1.3 mL, 0.65 mmol) which contains KO^{t}Bu (0.5 mmol) and *i*-BuOH (0.65 mmol) was added. After 24 h stirring, activated benzenepropyl-9BBN solution (2 mL, 1mmol) which contains KO^{t}Bu (0.7 mmol) and *i*-BuOH (1 mmol) was added and the reaction mixture was stirred for another 48 h at r.t. before filtering through a silica gel, eluting with Et_2O (30 mL). The solvent was removed by rotary evaporation, and the residue was purified by chromatography using an eluent of hexane/dichloromethane. The product ((*S*)-(2-propylpentane-1,5-diyl)dibenzene), was obtained as a colorless oil (113 mg, 85%).

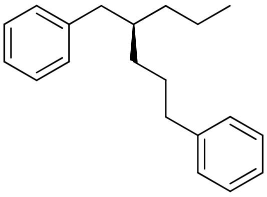

(*S*)-(2-propylpentane-1,5-diyl)dibenzene

85%; Colorless oil; $[\alpha]_D^{25}$ + 12.5 (*c* 1.0, $CHCl_3$, 96% ee); IR (neat) 3027, 1321, 998, 746; ^1H NMR ($CDCl_3$, 200 MHz): δ 7.32-7.08 (m, 10H), 2.63-2.56 (m, 4H), 1.74-1.59 (m, 3H), 1.31-1.27 (m, 6H), 0.93 (t, *J* = 6.0 Hz, 3H); ^{13}C NMR ($CDCl_3$, 50 MHz): δ 143.0, 141.7, 129.2, 128.4, 128.3, 128.1, 125.6, 125.5, 40.5, 39.4, 36.3, 35.5, 32.8, 28.6, 19.7, 14.4; HRMS (APCI) calcd for $C_{20}H_{27}$ $[M]^+$: 267.2113; found: 267.2102; HPLC (Daicel Chiralcel OJ-H, *i*-PrOH/hexane = 1.5/98.5, 1.0 mL/min, 190 nm) t1 = 6.5 min (major), t2 = 7.2 min (minor).

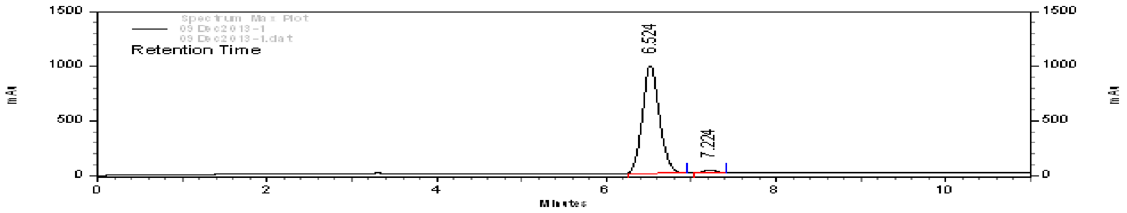

Spectrum Max Plot Results				
Retention Time	Area	Area Percent	Height	
6.524	13676632	98.066	986927	97.499
7.224	269725	1.934	25316	2.501
Totals				
	13946357	100.000	1012243	100.000

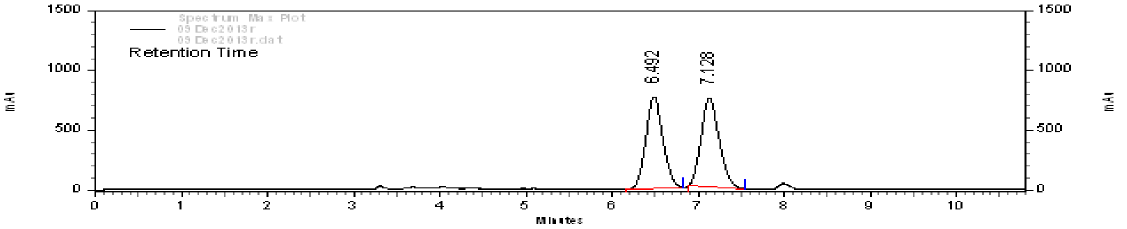

Spectrum Max Plot Results				
Retention Time	Area	Area Percent	Height	
6.492	10684829	49.436	771767	50.836
7.128	10928440	50.564	746376	49.164
Totals				
	21613269	100.000	1518143	100.000

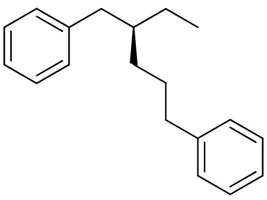

(*S*)-(2-ethylpentane-1,5-diyl)dibenzene

79%; Colorless oil; $[\alpha]_D^{25}$ + 9.1 (*c* 1.0, $CHCl_3$, 92% ee); IR (neat) 3028, 1314, 1092, 768; ^1H NMR ($CDCl_3$, 400 MHz): δ 7.24-7.08 (m, 10H), 2.54-2.48 (m, 3H), 1.62-1.57 (m, 4H), 1.23-1.20 (m, 4H), 0.84 (t, *J* = 7.2 Hz, 3H); ^{13}C NMR ($CDCl_3$, 100 MHz): δ 142.8, 141.7, 129.2, 128.4, 128.2, 128.1, 125.6, 125.5, 41.2, 40.1, 36.3, 32.3, 28.6, 25.4, 10.8; HRMS (APCI) calcd for $C_{19}H_{25}$ $[M]^+$: 253.1956; found: 253.1960; HPLC (Daicel Chiralcel OJ-H, *i*-PrOH/hexane = 1.5/98.5, 1.0 mL/min, 190 nm) t1 = 8.2 min (major), t2 = 9.6 min (minor).

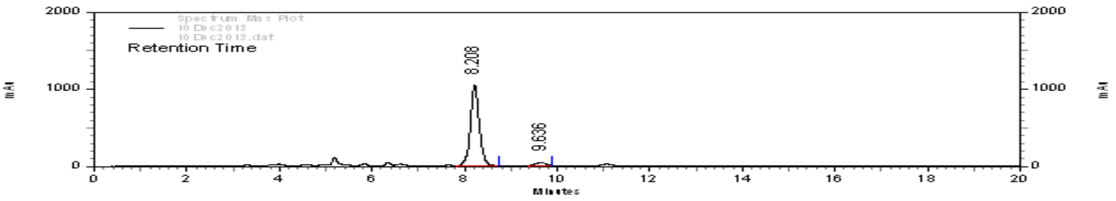

Spectrum Max Plot Results				
Retention Time	Area	Area Percent	Height	
8.208	13704878	96.137	1040036	96.322
9.636	550664	3.863	39713	3.678
Totals				
	14255542	100.000	1079749	100.000

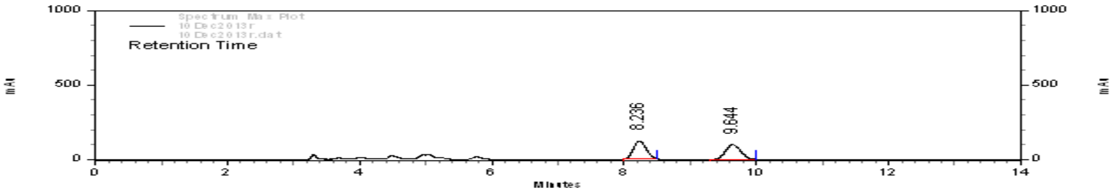

Spectrum Max Plot Results				
Retention Time	Area	Area Percent	Height	
8.236	1620901	50.361	120687	54.741
9.644	1597652	49.639	99783	45.259
Totals				
	3218553	100.000	220470	100.000

Determination of absolute configuration

The absolute configuration of the double cross-coupling product obtained with ligand (*R*,*R*)-**2** was determined to be (*S*) by comparison to the reported optical rotation[2].

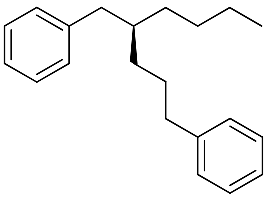

This result: $[\alpha]_D^{25}$ + 7.9 (*c* 1.0, $CHCl_3$, 98% ee)

Reference 2: $[\alpha]_D^{22}$ + 8.5 (*c* 1.0, $CHCl_3$, 93% ee)

(*S*)-(2-butylpentane-1,5-diyl)dibenzene[2]

80%; $[\alpha]_D^{25}$ + 7.9 (*c* 1.0, $CHCl_3$, 98% ee); ^{1}H NMR ($CDCl_3$, 200 MHz): δ 7.33-7.12 (m, 10H), 2.61-2.53 (m, 4H), 1.76-1.56 (m, 3H), 1.38-1.26 (m, 7H), 0.95-0.81 (m, 4H); ^{13}C NMR ($CDCl_3$, 50 MHz): δ 142.9, 141.7, 129.2, 128.3, 128.2, 128.1, 125.6, 125.5, 40.5, 39.5, 36.3, 32.8, 28.8, 28.5, 23.0, 14.1; HPLC (Daicel Chiralcel OD-H, *i*-PrOH/hexane = 0/100, 1.0 mL/min, 190 nm) t1 = 13.3 min (minor), t2 = 21.6 min (major).

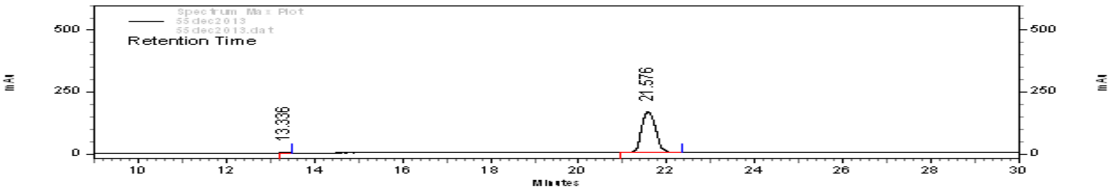

Spectrum Max Plot Results<br>Retention Time	Area	Area Percent	Height	
13.336	32379	0.887	3315	1.987
21.576	3617526	99.113	163538	98.013
Totals	3649905	100.000	166853	100.000

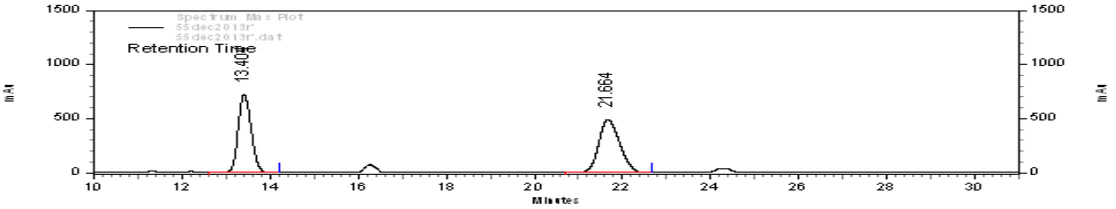

Spectrum Max Plot Results<br>Retention Time	Area	Area Percent	Height	
13.404	14824646	48.237	722319	59.798
21.664	15908105	51.763	485604	40.202
Totals	30732751	100.000	1207923	100.000

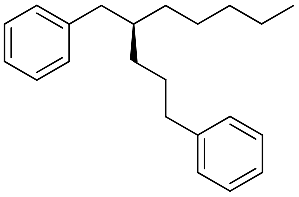

(*S*)-(2-pentylpentane-1,5-diyl)dibenzene

81%; Colorless oil; $[\alpha]_D^{25}$ + 6.7 (*c* 1.0, $CHCl_3$, 80% ee); IR (neat) 3019, 1300, 982, 708; ^1H NMR ($CDCl_3$, 300 MHz): δ 7.28-7.10 (m, 10H), 2.57-2.50 (m, 4H), 1.60-1.58 (m, 3H), 1.34-1.22 (m, 11H), 0.88 (t, *J* = 6.6 Hz, 3H); ^{13}C NMR ($CDCl_3$, 75 MHz): δ 142.6, 141.3, 129.1, 128.3, 128.1, 128.0, 125.5, 125.4, 40.4, 39.5, 36.2, 32.9, 32.6, 32.1, 28.4, 26.1, 22.6, 14.0; HRMS (APCI) calcd for $C_{22}H_{31}$ $[M+1]^+$: 295.2426; found: 295.2429. HPLC (Daicel Chiralcel OD-H, *i*-PrOH/hexane = 0.2/99.8, 1.0 mL/min, 190 nm) t1 = 11.8 min (minor), t2 = 17.8 min (major).

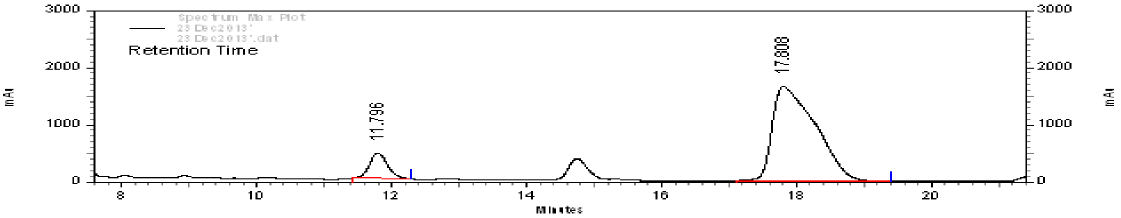

Spectrum Max Plot Results				
Retention Time	Area	Area Percent	Height	
11.796	8409734	10.227	450330	21.430
17.808	73817469	89.773	1651030	78.570
Totals				
	82227203	100.000	2101360	100.000

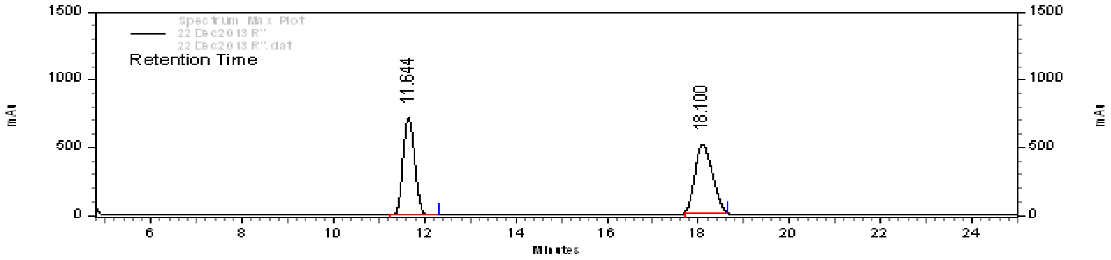

Spectrum Max Plot Results				
Retention Time	Area	Area Percent	Height	
11.644	12813448	48.628	719005	58.848
18.100	13536232	51.372	502800	41.152
Totals				
	26349680	100.000	1221805	100.000

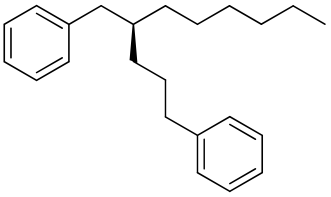

(*S*)-(2-hexylpentane-1,5-diyl)dibenzene

83%; Colorless oil; $[\alpha]_D^{25}$ - 3.2 (*c* 1.0, $CHCl_3$, 89% ee); IR (neat) 3020, 1294, 987, 729; ^1H NMR ($CDCl_3$, 200 MHz): δ 7.33-7.12 (m, 10H), 2.62-2.54 (m, 4H), 1.70-1.55 (m, 4H), 1.38-1.27 (m, 12H), 0.93 (t, *J* = 6.1 Hz, 3H); ^{13}C NMR ($CDCl_3$, 50 MHz): δ 142.8.0, 141.7, 129.2, 128.4, 128.2, 128.1, 125.6, 40.5, 39.6, 36.3, 33.1, 32.8, 31.9, 29.7, 28.5, 26.5, 22.7, 14.1; HRMS (APCI) calcd for $C_{23}H_{33}$ $[M+1]^+$: 309.2582; found: 309.2589. HPLC (Daicel Chiralcel OD-H, *i*-PrOH/hexane = 0.5/99.5, 1.0 mL/min, 190 nm) t1 = 9.2 min (major), t2 = 13.3 min (minor).

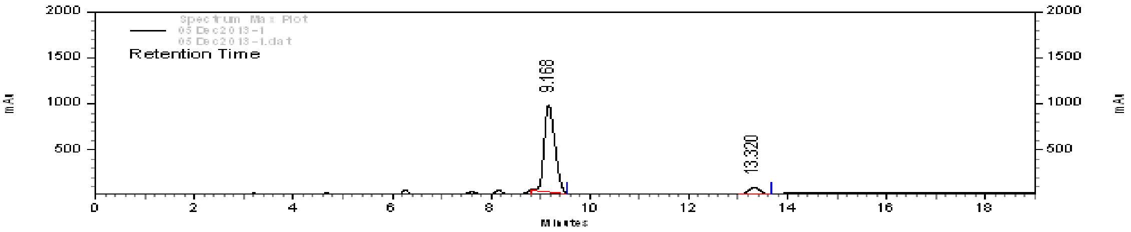

Spectrum Max Plot Results				
Retention Time	Area	Area Percent	Height	
9.168	14259245	94.508	938157	94.040
13.320	828689	5.492	59455	5.960
Totals				
	15087934	100.000	997612	100.000

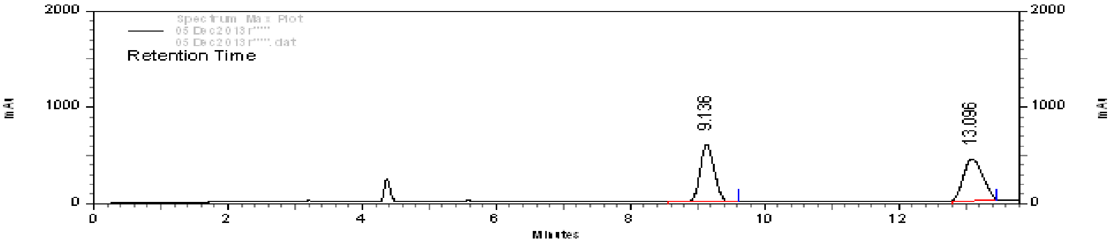

Spectrum Max Plot Results				
Retention Time	Area	Area Percent	Height	
9.136	8301800	49.169	587014	57.707
13.096	8582537	50.831	430213	42.293
Totals				
	16884337	100.000	1017227	100.000

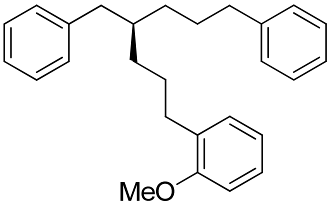

(*S*)-(2-(3-(2-methoxyphenyl)propyl)pentane-1,5-diyl)dibenzene

68%; Colorless oil; $[\alpha]_D^{25}$ - 14.6 (*c* 1.0, $CHCl_3$, 78% ee); IR (neat) 3017, 1287, 1011, 705; ^{1}H NMR ($CDCl_3$, 200 MHz): δ 7.35-6.77 (m, 14H), 3.85 (s, 3H), 2.64-2.48 (m, 6H), 1.73-1.59 (m, 5H), 1.39-1.26 (m, 4H); ^{13}C NMR ($CDCl_3$, 50 MHz): δ 157.4, 142.8, 141.6, 131.2, 129.8, 129.3, 128.4, 128.3, 128.1, 126.9, 125.6, 125.5, 120.3, 110.2, 55.3, 40.4, 39.3, 32.9, 32.7, 30.5, 28.5, 26.8; HRMS (APCI) calcd for $C_{27}H_{33}O$ $[M+1]^+$: 373.2531; found: 373.2516. HPLC (Daicel Chiralcel OD-H, *i*-PrOH/hexane = 1.5/98.5, 1.0 mL/min, 190 nm) t1 = 8.9 min (minor), t2 = 9.7 min (major).

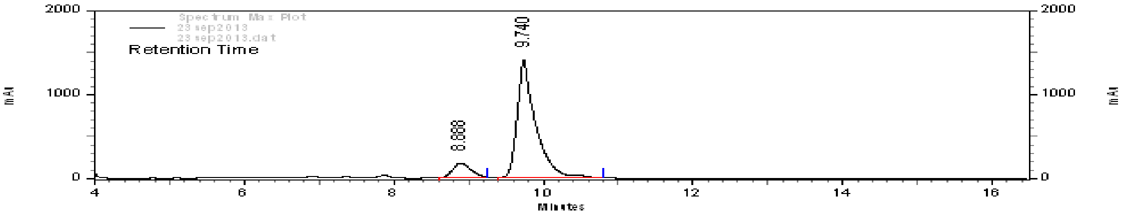

Spectrum Max Plot Results				
Retention Time	Area	Area Percent	Height	
8.888	2894026	10.807	171796	10.913
9.740	23885945	89.193	1402401	89.087
Totals				
	26779971	100.000	1574197	100.000

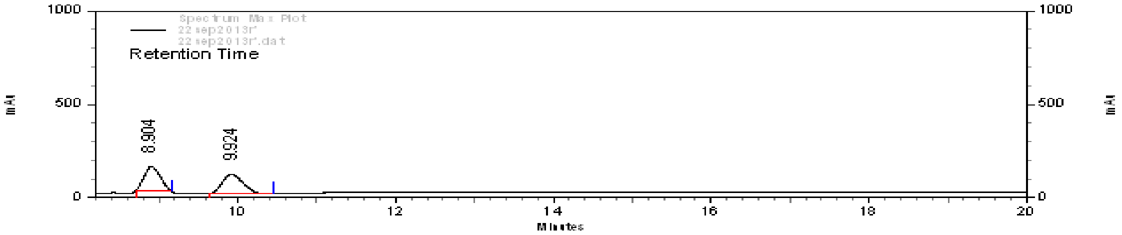

Spectrum Max Plot Results				
Retention Time	Area	Area Percent	Height	
8.904	1850823	51.796	131440	57.179
9.924	1722458	48.204	98433	42.821
Totals				
	3573281	100.000	229873	100.000

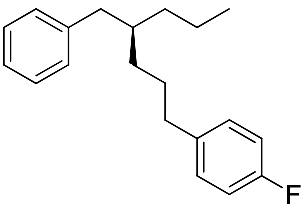

(*S*)-1-(4-benzylheptyl)-4-fluorobenzene

81%; Colorless oil; $[\alpha]_D^{25}$ + 10.3 (*c* 1.0, $CHCl_3$, 92% ee); IR (neat) 3040, 3012, 1491, 1452, 798; ^{1}H NMR ($CDCl_3$, 400 MHz): δ 7.25-6.90 (m, 9H), 2.55-2.43 (m, 4H), 1.66-1.55 (m, 3H), 1.29-1.19 (m, 6H), 0.85 (t, *J* = 6.8 Hz, 3H); ^{13}C NMR ($CDCl_3$, 100 MHz): δ 162.4 (d, *J* = 241.3 Hz), 141.6, 138.4 (d, *J* = 3.1 Hz), 129.7 (d, *J* = 7.7 Hz), 129.2, 128.3 (d, *J* = 15.8 Hz), 125.6, 115.0 (d, *J* = 20.9 Hz), 40.5, 39.4, 35.6, 35.4, 35.1, 32.6, 31.5, 29.0, 28.6, 19.7, 14.4; ^{19}F NMR ($CDCl_3$, 188 MHz): δ -121.4; HRMS (APCI) calcd for $C_{20}H_{26}F$ $[M+1]^+$: 285.2019; found: 285.2023; HPLC (Daicel Chiralcel OJ-H, *i*-PrOH/hexane = 1.5/98.5, 1.0 mL/min, 190 nm) t1 = 6.2 min (minor), t2 = 6.9 min (major).

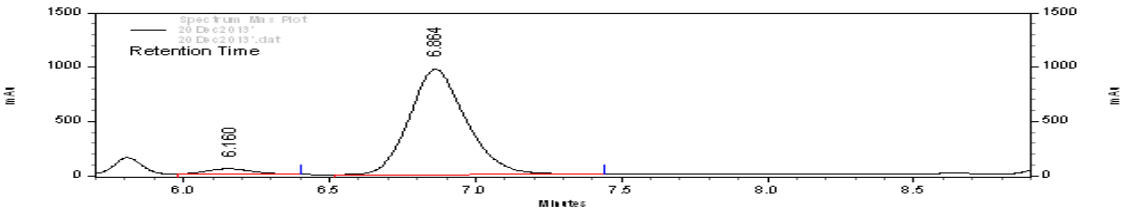

Spectrum Max Plot Results				
Retention Time	Area	Area Percent	Height	
6.160	539273	3.958	49174	4.814
6.864	13085748	96.042	972288	95.186
Totals				
	13625021	100.000	1021462	100.000

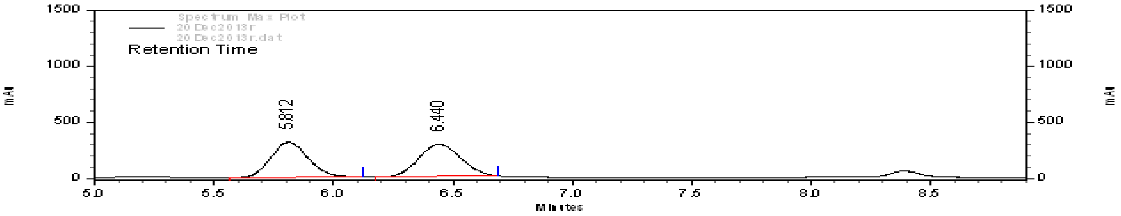

Spectrum Max Plot Results				
Retention Time	Area	Area Percent	Height	
5.812	3417457	49.177	313649	52.176
6.440	3531887	50.823	287487	47.824
Totals				
	6949344	100.000	601136	100.000

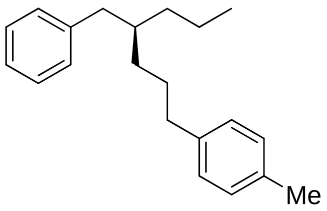

(*S*)-1-(4-benzylheptyl)-4-methylbenzene

85%; Colorless oil; $[\alpha]_D^{25}$ + 20.6 (*c* 1.0, CHCl3, 87% ee); IR (neat) 3044, 3011, 1495, 1453, 799; ^{1}H NMR ($CDCl_3$, 200 MHz): δ 7.29-7.08 (m, 9H), 2.58-2.51 (m, 4H), 2.36 (s, 3H), 1.56-1.35 (m, 3H), 1.34-1.27 (m, 6H), 0.91 (t, *J* = 7.8 Hz, 3H); ^{13}C NMR ($CDCl_3$, 50 MHz): δ 142.1, 139.9, 135.1, 129.2, 128.9, 128.2, 128.1, 40.5, 39.4, 35.9, 35.5, 32.9, 28.8, 21.0, 19.7, 14.5; HRMS (APCI) calcd for $C_{21}H_{29}$ $[M+1]^+$: 281.2269; found: 281.2273. HPLC (Daicel Chiralcel OJ-H, *i*-PrOH/hexane = 4/96, 1.0 mL/min, 190 nm) t1 = 4.8 min (minor), t2 = 5.4 min (major).

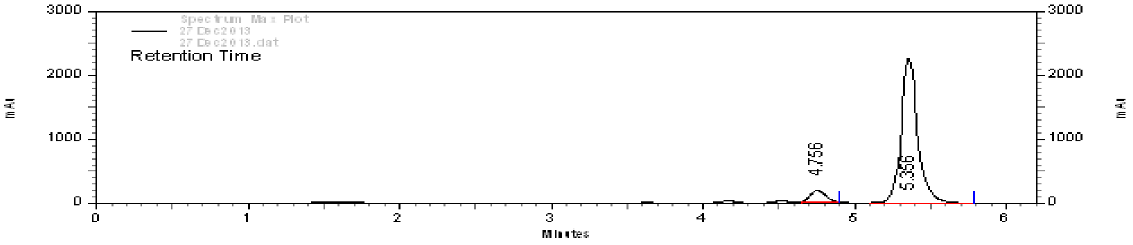

Spectrum Max Plot Results				
Retention Time	Area	Area Percent	Height	
4.756	1229157	6.614	180391	7.417
5.356	17355870	93.386	2251890	92.583
Totals				
	18585027	100.000	2432281	100.000

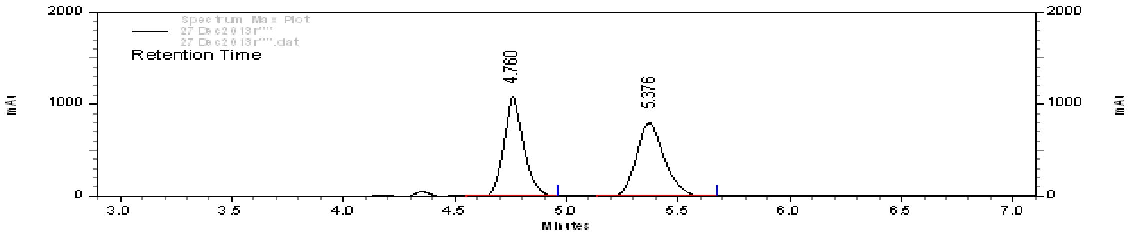

Spectrum Max Plot Results				
Retention Time	Area	Area Percent	Height	
4.760	6484724	48.905	1078324	57.610
5.376	6775040	51.095	793433	42.390
Totals				
	13259764	100.000	1871757	100.000

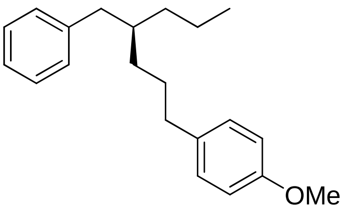

(*S*)-1-(4-benzylheptyl)-4-methoxybenzene

88%; Colorless oil; $[\alpha]_D^{25}$ + 10.1 (*c* 1.0, $CHCl_3$, 82% ee); IR (neat) 3042, 3016, 1493, 1450, 792; ^1H NMR ($CDCl_3$, 400 MHz): δ 7.24-6.77 (m, 9H), 3.76 (s, 3H), 2.57-2.44 (m, 4H), 1.66-1.55 (m, 3H), 1.28-1.15 (m, 6H), 0.94 (t, J = 6.8 Hz, 3H); ^{13}C NMR ($CDCl_3$, 100 MHz): δ 157.6, 141.7, 134.9, 129.2, 129.1, 128.1, 125.5, 113.7, 55.3, 40.5, 39.4, 35.5, 35.3, 32.7, 28.8, 19.7, 14.4; HRMS (APCI) calcd for $C_{21}H_{29}O$ $[M+1]^+$: 297.2218; found: 297.2173. HPLC (Daicel Chiralcel OJ-H, *i*-PrOH/hexane = 1.5/98.5, 1.0 mL/min, 190 nm) t1 = 11.6 min (minor), t2 = 14.0 min (major).

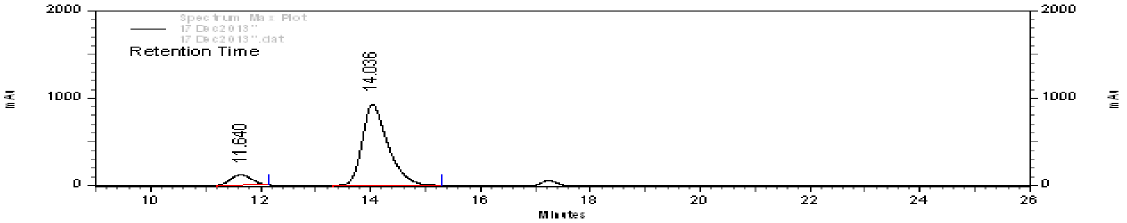

Spectrum Max Plot Results				
Retention Time	Area	Area Percent	Height	
11.640	2998757	9.158	114143	10.929
14.036	29746242	90.842	930236	89.071
Totals				
	32744999	100.000	1044379	100.000

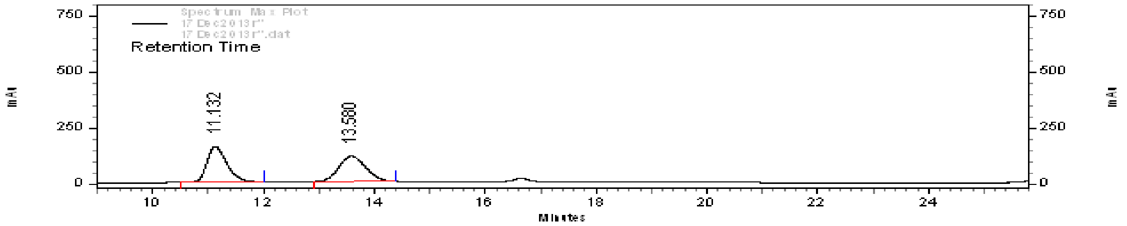

Spectrum Max Plot Results				
Retention Time	Area	Area Percent	Height	
11.132	3873340	50.253	156274	58.197
13.580	3834364	49.747	112251	41.803
Totals				
	7707704	100.000	268525	100.000

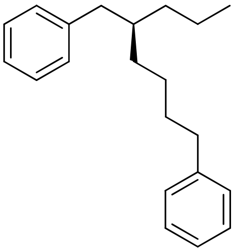

(*S*)-(2-propylhexane-1,6-diyl)dibenzene

81%; Colorless oil; $[\alpha]_D^{25}$ + 7.4 (*c* 1.0, $CHCl_3$, 78% ee); IR (neat) 3040, 3017, 1490, 1452, 799; ^{1}H NMR ($CDCl_3$, 400 MHz): δ 7.24-7.08 (m, 10H), 2.58-2.48 (m, 4H), 1.60-1.50 (m, 4H), 1.32-1.18 (m, 7H), 0.84 (t, J = 7.6 Hz, 3H); ^{13}C NMR ($CDCl_3$, 100 MHz): δ 142.7, 141.6, 129.1, 128.3, 128.1, 128.0, 125.4, 125.3, 40.4, 39.3, 35.8, 35.4, 32.8, 31.7, 26.1, 19.6, 14.3; HRMS (APCI) calcd for $C_{21}H_{29}$ $[M+1]^+$: 281.2245; found: 281.2254. HPLC (Daicel Chiralcel OJ-H, *i*-PrOH/hexane = 1/99, 1.0 mL/min, 190 nm) t1 = 13.8 min (minor), t2 = 16.2 min (major).

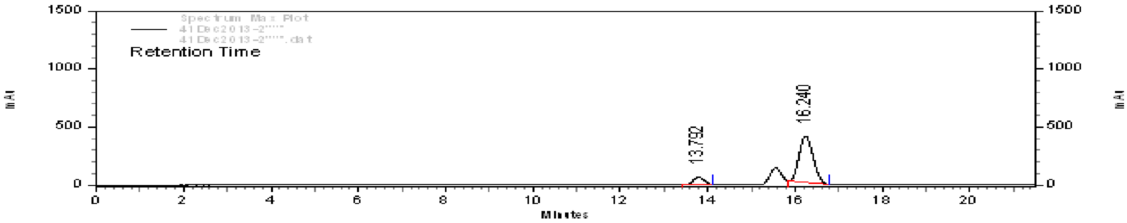

Spectrum Max Plot Results				
Retention Time	Area	Area Percent	Height	
13.792	1132423	11.145	67064	14.339
16.240	9027985	88.855	400655	85.661
Totals				
	10160408	100.000	467719	100.000

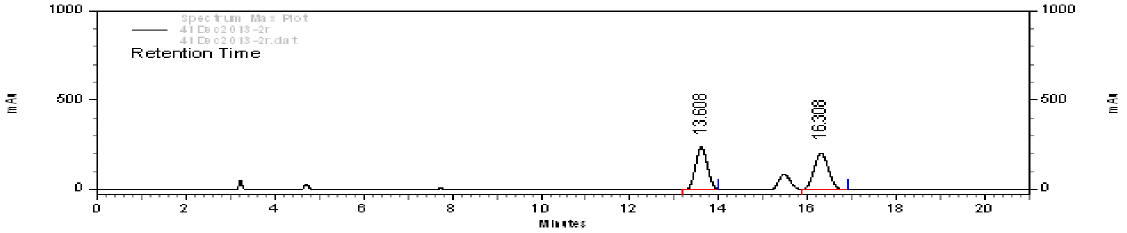

Spectrum Max Plot Results				
Retention Time	Area	Area Percent	Height	
13.608	4305038	49.296	234438	53.759
16.308	4427962	50.704	201651	46.241
Totals				
	8733000	100.000	436089	100.000

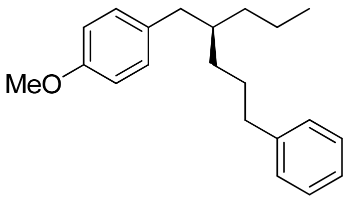

(*S*)-1-methoxy-4-(5-phenyl-2-propylpentyl)benzene

82%; Colorless oil; $[\alpha]_D^{25}$ + 5.4 (*c* 1.0, $CHCl_3$, >99% ee); IR (neat) 3029, 3015, 1458, 704; ^{1}H NMR ($CDCl_3$, 200 MHz): δ 7.34-6.80 (m, 9H), 3.81 (s, 3H), 2.61-2.47 (m, 4H), 1.71-1.36 (m, 9H), 0.91 (t, *J* = 6.0 Hz, 3H); ^{13}C NMR ($CDCl_3$, 50 MHz): δ 157.5, 142.8, 133.7, 130.0, 128.4, 128.2, 125.6, 113.5, 55.2, 39.5, 36.3, 35.5, 32.7, 28.6, 19.7, 14.4; HRMS (APCI) calcd for $C_{21}H_{29}O$ $[M+1]^+$: 297.2218; found: 297.2220; HPLC (Daicel Chiralcel OJ-H, *i*-PrOH/hexane = 4/96, 1.0 mL/min, 190 nm) t1 = 6.7 min (minor), t2 = 8.1 min (major).

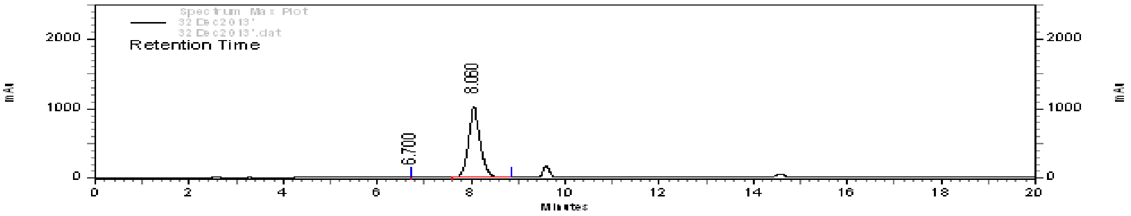

Spectrum Max Plot Results				
Retention Time	Area	Area Percent	Height	
6.700	0	0.000	0	0.000
8.060	16967386	100.000	1010643	100.000
Totals				
	16967386	100.000	1010643	100.000

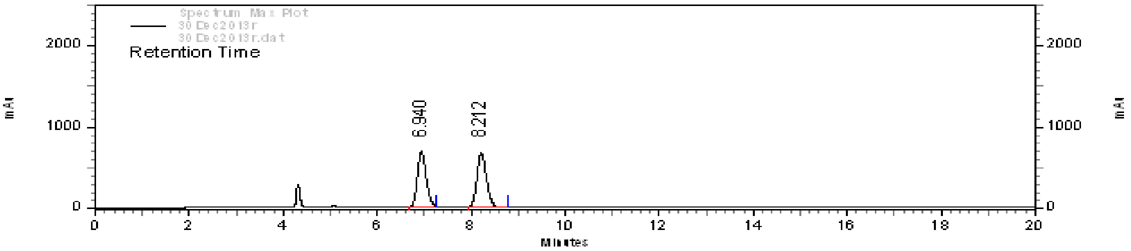

Spectrum Max Plot Results				
Retention Time	Area	Area Percent	Height	
6.940	8828168	49.264	677877	50.550
8.212	9091832	50.736	663117	49.450
Totals				
	17920000	100.000	1340994	100.000

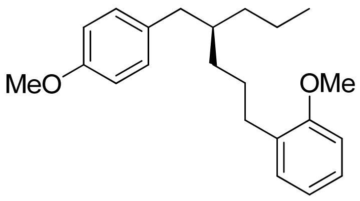

(*S*)-1-methoxy-2-(4-(4-methoxybenzyl)heptyl)benzene

88%; Colorless oil; $[\alpha]_D^{25}$ + 16.7 (*c* 1.0, $CHCl_3$, >99% ee); IR (neat) 3044, 3017, 1464, 731; ^{1}H NMR ($CDCl_3$, 200 MHz): δ 7.27-6.82 (m, 8H), 3.85 (s, 3H), 3.82 (s, 3H), 2.51-2.48 (m, 4H), 1.72-1.23 (m, 9H), 0.92 (t, *J* = 6.0 Hz, 3H); ^{13}C NMR ($CDCl_3$, 50 MHz): δ 134.1, 131.5, 130.1, 129.8, 126.8, 120.3, 113.5, 110.2, 55.2, 39.5, 35.4, 32.9, 30.5, 26.8, 19.7, 14.5; HRMS (APCI) calcd for $C_{22}H_{31}O_2$ $[M+1]^+$: 327.2324; found: 327.2320; HPLC (Daicel Chiralcel OJ-H, *i*-PrOH/hexane = 2/98, 1.0 mL/min, 190 nm) t1 = 9.1 min (minor), t2 = 9.8 min (major).

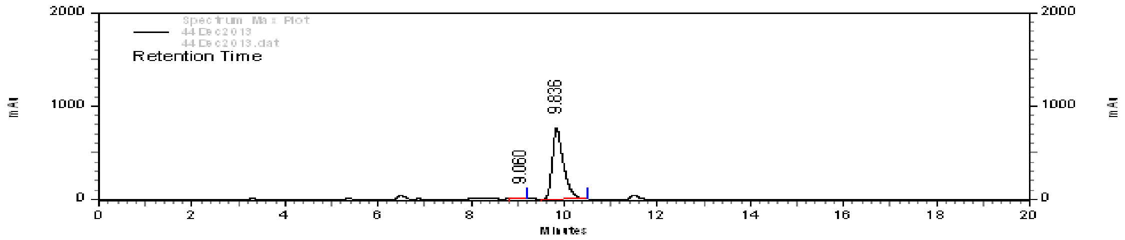

Spectrum Max Plot Results				
Retention Time	Area	Area Percent	Height	
9.060	32291	0.255	4642	0.601
9.836	12635003	99.745	768148	99.399
Totals				
	12667294	100.000	772790	100.000

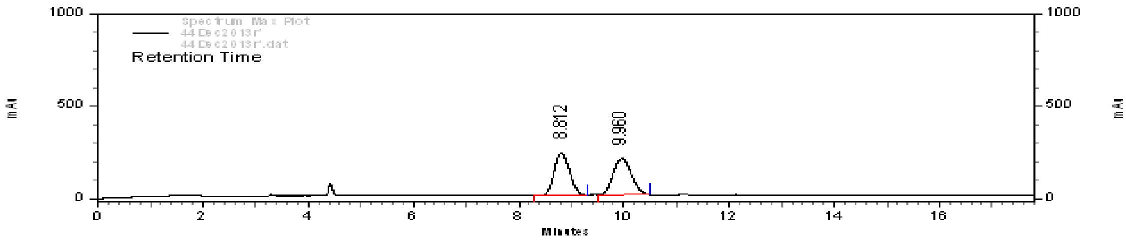

Spectrum Max Plot Results				
Retention Time	Area	Area Percent	Height	
8.812	4674004	49.944	231025	54.225
9.960	4684441	50.056	195022	45.775
Totals				
	9358445	100.000	426047	100.000

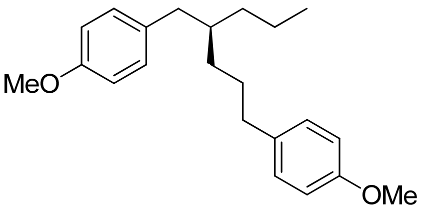

(*S*)-4,4'-(2-propylpentane-1,5-diyl)bis(methoxybenzene)

87%; Colorless oil; $[\alpha]_D^{25}$ + 12.9 (*c* 1.0, $CHCl_3$, 94% ee); IR (neat) 3062, 3013, 1467, 722; ^1H NMR ($CDCl_3$, 300 MHz): δ 7.06-6.77 (m, 8H), 3.78 (s, 6H), 2.49-2.40 (m, 4H), 1.62-1.18 (m, 9H), 0.86 (t, J = 6.3 Hz, 3H); ^{13}C NMR ($CDCl_3$, 75 MHz): δ 134.8, 133.8, 129.9, 129.1, 113.5, 113.4, 55.1, 39.4, 35.3, 35.2, 32.5, 28.7, 19.6, 14.3; HRMS (APCI) calcd for $C_{22}H_{31}O_2$ $[M+1]^+$: 327.2324; found: 327.2320. HPLC (Daicel Chiralcel OD-H, *i*-PrOH/hexane = 2/98, 1.0 mL/min, 190 nm) t1 = 5.9 min (minor), t2 = 6.3 min (major).

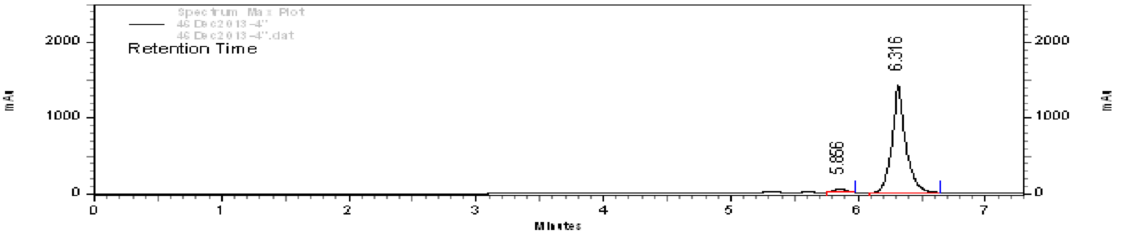

Spectrum Max Plot Results				
Retention Time	Area	Area Percent	Height	
5.856	304071	2.785	42003	2.869
6.316	10613184	97.215	1422228	97.131
Totals				
	10917255	100.000	1464231	100.000

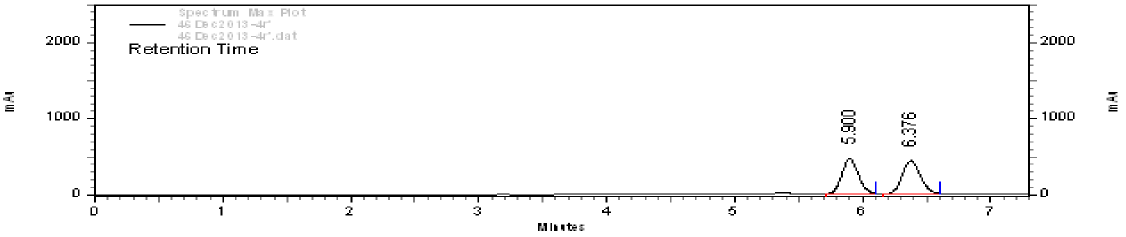

Spectrum Max Plot Results				
Retention Time	Area	Area Percent	Height	
5.900	4074350	48.954	466404	51.748
6.376	4248490	51.046	434895	48.252
Totals				
	8322840	100.000	901299	100.000

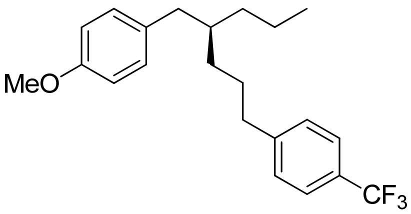

(*S*)-1-methoxy-4-(2-propyl-5-(4-(trifluoromethyl)phenyl)pentyl)benzene

82%; Colorless oil; $[\alpha]_D^{25}$ + 8.6 (*c* 1.0, $CHCl_3$, 95% ee); IR (neat) 3031, 3018, 1457, 709; ^{1}H NMR ($CDCl_3$, 400 MHz): δ 7.48-6.75 (m, 8H), 3.75 (s, 3H), 2.58-2.35 (m, 4H), 1.61-1.17 (m, 9H), 0.84 (t, *J* = 6.8 Hz, 3H); ^{13}C NMR ($CDCl_3$, 100 MHz): δ 157.7, 146.9, 133.5, 130.0, 128.7, 127.8, 125.2 (q, *J* = 3.7 Hz), 113.5, 55.3, 55.2, 39.4, 36.0, 35.5, 32.5, 28.2, 19.8, 14.4; ^{19}F NMR ($CDCl_3$, 188 MHz): δ -65.5; HRMS (APCI) calcd for $C_{22}H_{27}OF_3$ $[M]^+$: 364.2014; found: 364.2037. HPLC (Daicel Chiralcel OD-H, *i*-PrOH/hexane = 0/100, 1.0 mL/min, 190 nm) t1 = 24.3 min (minor), t2 = 30.9 min (major).

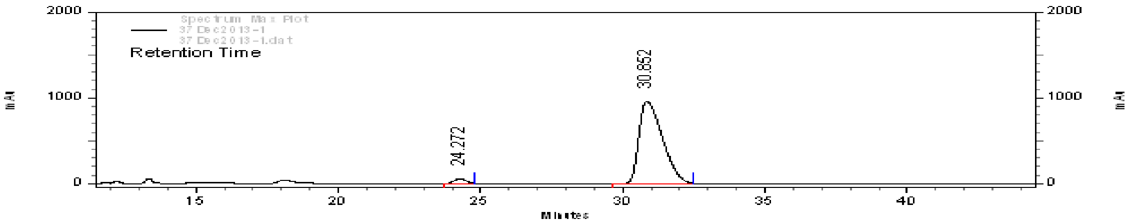

Spectrum Max Plot Results				
Retention Time	Area	Area Percent	Height	
24.272	1496687	2.677	52130	5.184
30.852	54413412	97.323	953369	94.816
Totals				
	55910099	100.000	1005499	100.000

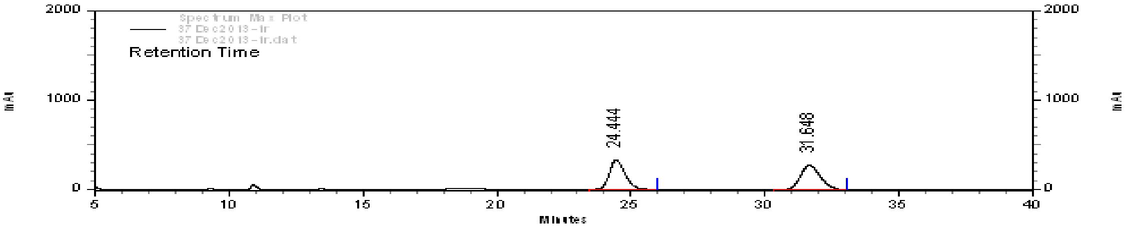

Spectrum Max Plot Results				
Retention Time	Area	Area Percent	Height	
24.444	12967234	49.364	321676	54.705
31.648	13301378	50.636	266340	45.295
Totals				
	26268612	100.000	588016	100.000

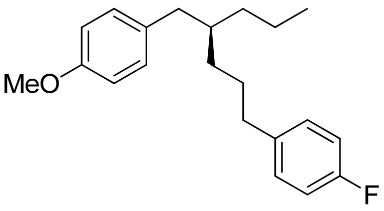

(*S*)-1-fluoro-4-(4-(4-methoxybenzyl)heptyl)benzene

85%; Colorless oil; $[\alpha]_D^{25}$ + 3.1 (*c* 1.0, $CHCl_3$, 94% ee); IR (neat) 3040, 3021, 1462, 723; ^1H NMR ($CDCl_3$, 400 MHz): δ 7.06-6.76 (m, 8H), 3.76 (s, 3H), 2.48-2.39 (m, 4H), 1.61-1.15 (m, 9H), 0.84 (t, *J* = 6.8 Hz, 3H); ^{13}C NMR ($CDCl_3$, 100 MHz): δ 162.6 (d, *J* = 380.1 Hz), 138.3, 133.5, 129.9, 129.6(d, *J* = 7.7 Hz), 114.9 (d, *J* = 20.9 Hz), 113.4, 55.1, 39.4, 39.3, 35.4, 35.3, 32.4, 28.5, 19.6, 14.3; ^{19}F NMR ($CDCl_3$, 188 MHz): δ -121.4; HRMS (APCI) calcd for $C_{21}H_{27}FO$ $[M]^+$: 314.2040; found: 314.2034; HPLC (Daicel Chiralcel OJ-H, *i*-PrOH/hexane = 1/99, 1.0 mL/min, 190 nm) t1 = 25.9 min (minor), t2 = 27.6 min (major).

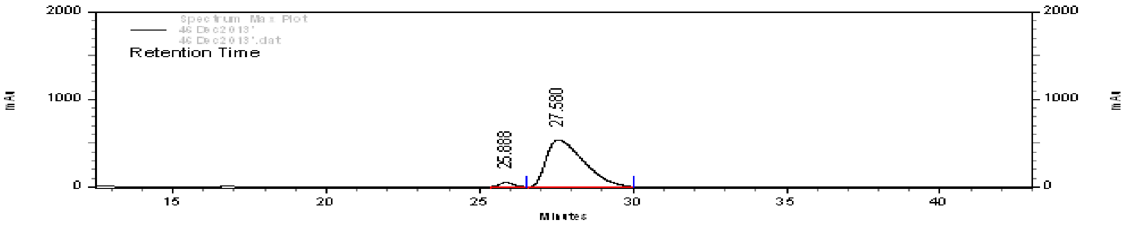

Spectrum Max Plot Results				
Retention Time	Area	Area Percent	Height	
25.888	1435957	3.106	48041	8.280
27.580	44799732	96.894	532145	91.720
Totals	46235689	100.000	580186	100.000

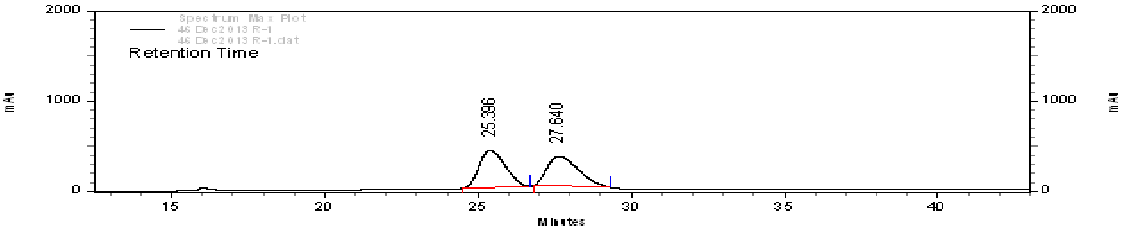

Spectrum Max Plot Results				
Retention Time	Area	Area Percent	Height	
25.396	23961701	50.586	405245	55.174
27.640	23406756	49.414	329244	44.826
Totals	47368457	100.000	734489	100.000

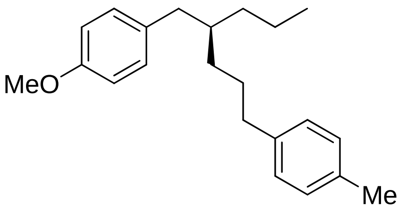

(*S*)-1-methoxy-4-(2-propyl-5-(p-tolyl)pentyl)benzene

86%; Colorless oil; $[\alpha]_D^{25}$ + 10.1 (*c* 1.0, $CHCl_3$, 92% ee); IR (neat) 3043, 3017, 1459, 711; ^1H NMR ($CDCl_3$, 400 MHz): δ 7.05-6.76 (m, 8H), 3.76 (s, 3H), 2.49-2.41 (m, 4H), 2.29 (s, 3H), 1.55-1.16 (m, 9H), 0.83 (t, J = 6.8 Hz, 3H); ^{13}C NMR ($CDCl_3$, 100 MHz): δ 157.6, 139.9, 134.9, 133.8, 129.9, 128.8, 128.1, 113.4, 55.1, 39.4, 35.7, 35.3, 32.6, 28.6, 20.9, 19.6, 14.3; HRMS (APCI) calcd for $C_{22}H_{30}O$ $[M]^+$: 310.2291; found: 310.2278; HPLC (Daicel Chiralcel OD-H, *i*-PrOH/hexane = 1/99, 1.0 mL/min, 190 nm) t1 = 5.9 min (minor), t2 = 7.4 min (major).

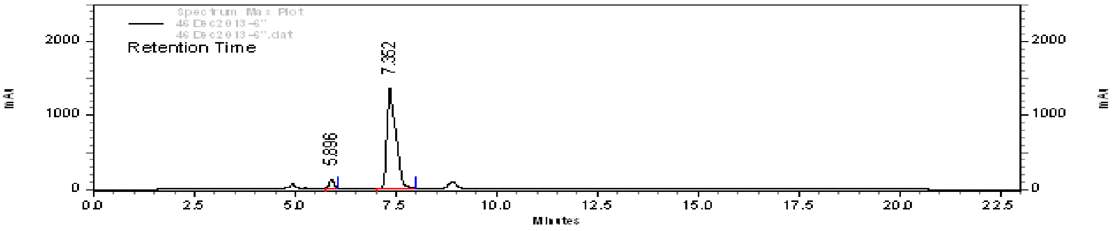

Spectrum Max Plot Results				
Retention Time	Area	Area Percent	Height	
5.896	888981	4.140	112674	7.712
7.352	20582338	95.860	1348361	92.288
Totals				
	21471319	100.000	1461035	100.000

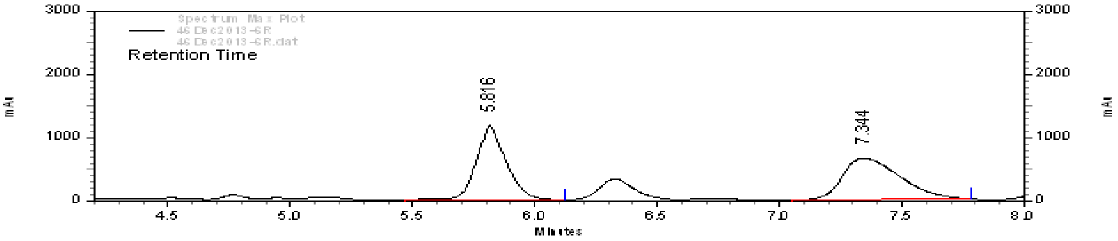

Spectrum Max Plot Results				
Retention Time	Area	Area Percent	Height	
5.816	9924809	48.291	1171455	64.147
7.344	10627128	51.709	654759	35.853
Totals				
	20551937	100.000	1826214	100.000

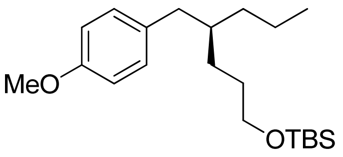

(*S*)-tert-butyl((4-(4-methoxybenzyl)heptyl)oxy)dimethylsilane

80%; Colorless oil; $[\alpha]_D^{25}$ + 9.4 (*c* 1.0, $CHCl_3$, 81% ee); IR (neat) 3020, 3007, 1458, 708; ^{1}H NMR ($CDCl_3$, 400 MHz): δ 7.08-6.80 (m, 4H), 3.80 (s, 3H), 3.60 (t, *J* = 6.0 Hz, 2H), 2.50-2.47 (m, 2H), 1.63-1.21 (m, 9H), 0.89-0.84 (m, 12H), 0(s, 6H); ^{13}C NMR ($CDCl_3$, 100 MHz): δ 157.6, 133.7, 130.0, 113.5, 63.6, 55.2, 39.5, 39.4, 35.4, 29.9, 29.8, 26.0, 19.7, 14.4, -5.3; HRMS (APCI) calcd for $C_{21}H_{39}O_2Si$ $[M+1]^+$: 351.2719; found: 351.2750; HPLC (Daicel Chiralcel OD-H, *i*-PrOH/hexane = 0/100, 1.0 mL/min, 190 nm) t1 = 12.3 min (minor), t2 = 15.2 min (major).

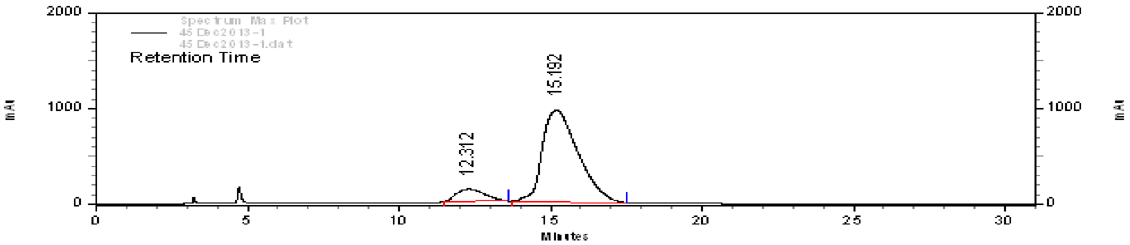

Spectrum Max Plot Results				
Retention Time	Area	Area Percent	Height	
12.312	8380830	9.638	128368	11.817
15.192	78574063	90.362	957942	88.183
Totals				
	86954893	100.000	1086310	100.000

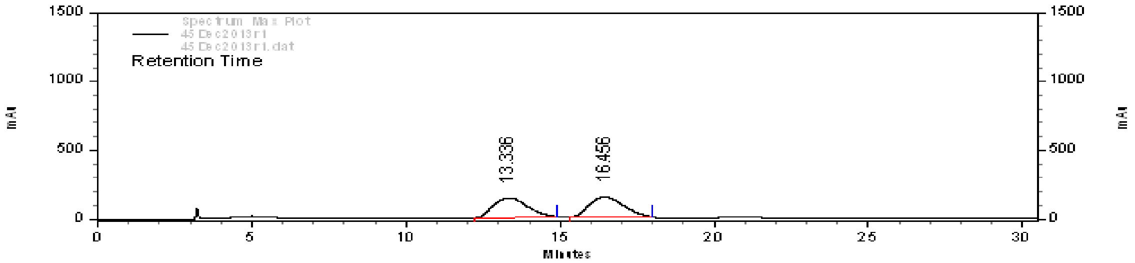

Spectrum Max Plot Results				
Retention Time	Area	Area Percent	Height	
13.336	10807591	48.723	140712	48.984
16.456	11373924	51.277	146550	51.016
Totals				
	22181515	100.000	287262	100.000

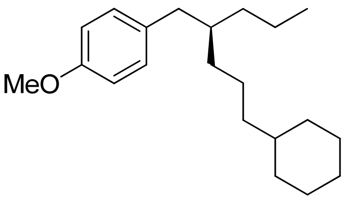

(*S*)-1-(5-cyclohexyl-2-propylpentyl)-4-methoxybenzene

84%; Colorless oil; $[\alpha]_D^{25}$ + 3.9 (*c* 1.0, $CHCl_3$, 78% ee); IR (neat) 3029, 3011, 1462, 705; ^{1}H NMR ($CDCl_3$, 400 MHz): δ 7.02-6.77 (m, 4H), 3.80 (s, 3H), 2.43-2.40 (m, 2H), 1.65-1.29 (m, 6H), 1.10-1.05 (m, 14H), 0.84-0.80 (m, 5H); ^{13}C NMR ($CDCl_3$, 100 MHz): 157.6, 133.9, 130.0, 113.5, 55.3, 55.1, 39.6, 37.9, 37.6, 35.5, 33.5, 33.4, 26.8, 26.5, 23.7, 19.8, 14.5; HRMS (APCI) calcd for $C_{21}H_{34}O$ $[M]^+$: 302.2604; found: 302.2589; HPLC (Daicel Chiralcel OD-H, *i*-PrOH/hexane = 0/100, 1.0 mL/min, 190 nm) t1 = 9.6 min (minor), t2 = 10.7 min (major).

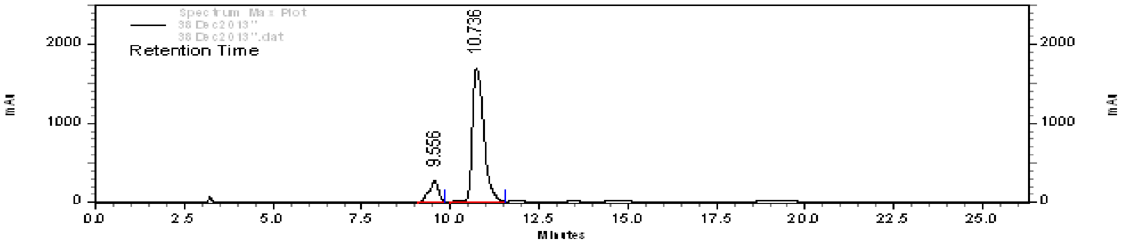

Spectrum Max Plot Results				
Retention Time	Area	Area Percent	Height	
9.556	4677062	10.995	262943	13.457
10.736	37859173	89.005	1690977	86.543
Totals				
	42536235	100.000	1953920	100.000

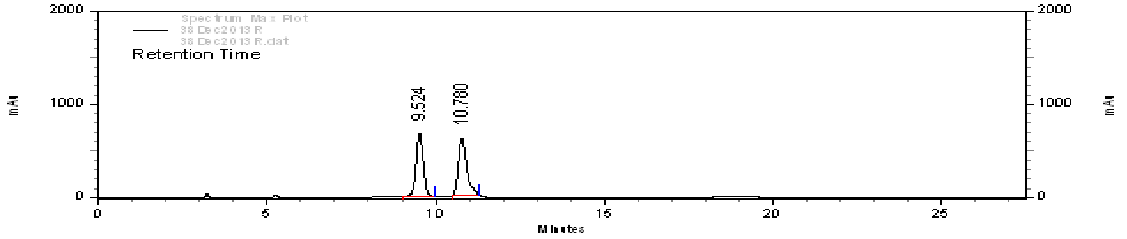

Spectrum Max Plot Results				
Retention Time	Area	Area Percent	Height	
9.524	9975756	48.503	668591	52.278
10.780	10591708	51.497	610321	47.722
Totals				
	20567464	100.000	1278912	100.000

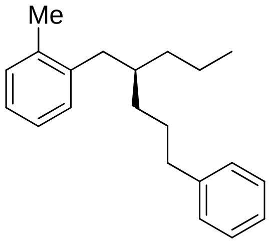

(*S*)-1-methyl-2-(5-phenyl-2-propylpentyl)benzene

78%; Colorless oil; $[\alpha]_D^{25}$ + 9.8 (*c* 1.0, $CHCl_3$, 87% ee); IR (neat) 3046, 3013, 1452, 978, 709; ^1H NMR ($CDCl_3$, 400 MHz): δ 7.27-7.01 (m, 9H), 2.61-2.49 (m, 3H), 2.27, (s, 3H), 1.68-1.47-1.26 (m, 10H), 0.87 (t, J = 6.4 Hz, 3H); ^{13}C NMR ($CDCl_3$, 100 MHz): δ 142.8, 139.9, 136.2, 130.2, 130.1, 128.4, 128.2, 125.7, 125.6, 38.1, 38.0, 36.6, 35.8, 33.1, 28.6, 19.7, 14.5; HPLC (Daicel Chiralcel OD-H, *i*-PrOH/hexane = 0/100, 1.0 mL/min, 190 nm) t1 = 12.6 min (minor), t2 = 13.8 min (major).

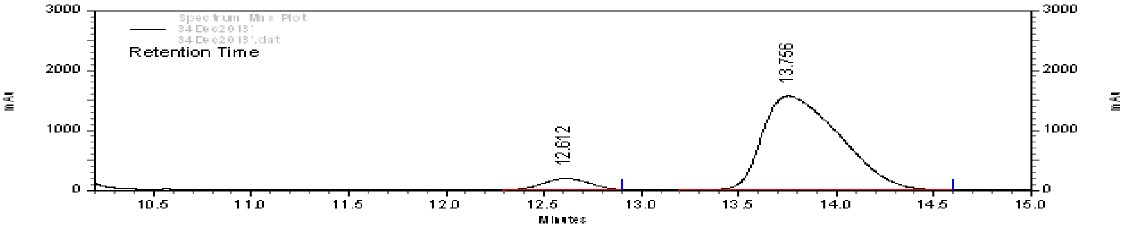

Spectrum Max Plot Results				
Retention Time	Area	Area Percent	Height	
12.612	2975827	6.464	181680	10.377
13.756	43058583	93.536	1569031	89.623
Totals				
	46034410	100.000	1750711	100.000

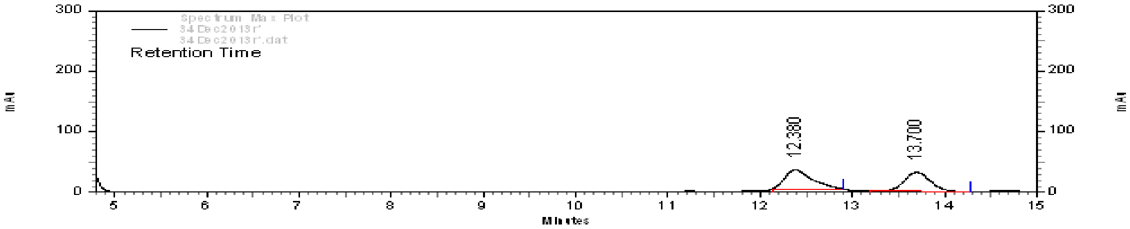

Spectrum Max Plot Results				
Retention Time	Area	Area Percent	Height	
12.380	654158	51.818	31572	50.412
13.700	608247	48.182	31056	49.588
Totals				
	1262405	100.000	62628	100.000

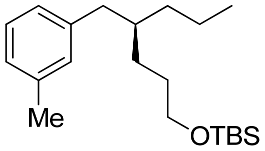

(*S*)-tert-butyldimethyl((4-(3-methylbenzyl)heptyl)oxy)silane

84%; Colorless oil; $[\alpha]_D^{25}$ + 5.7 (*c* 1.0, $CHCl_3$, 80% ee); IR (neat) 3037, 3019, 1448, 1415, 708; ^1H NMR ($CDCl_3$, 400 MHz): δ 7.14-6.90 (m, 4H), 3.54 (t, *J* = 6.8 Hz, 2H), 2.48 (t, *J* = 6.8 Hz, 2H), 2.29 (s, 3H), 1.64-1.30 (m, 9H), 1.19-0.84 (m, 12H), 0 (s, 6H); ^{13}C NMR ($CDCl_3$, 100 MHz): δ 140.1, 131.3, 130.5, 128.2, 63.5, 39.8, 35.4, 29.8, 28.9, 26.0, 18.4, 14.4, -5.2; HPLC (Daicel Chiralcel OD-H, *i*-PrOH/hexane = 0/100, 1.0 mL/min, 190 nm) t1 = 5.3 min (minor), t2 = 5.9 min (major).

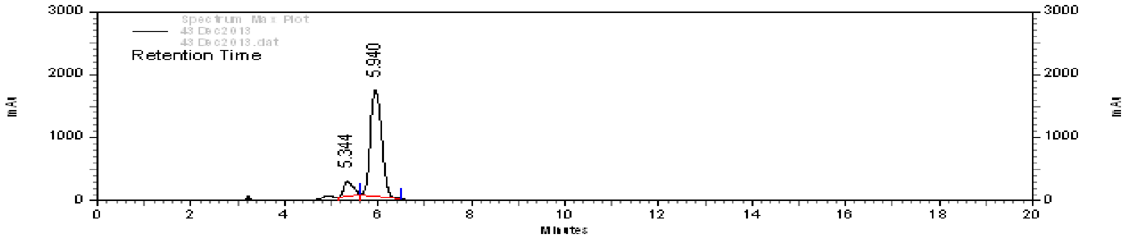

Spectrum Max Plot Results				
Retention Time	Area	Area Percent	Height	
5.344	3182253	10.191	236108	12.350
5.940	28044414	89.809	1675773	87.650
Totals	31226667	100.000	1911881	100.000

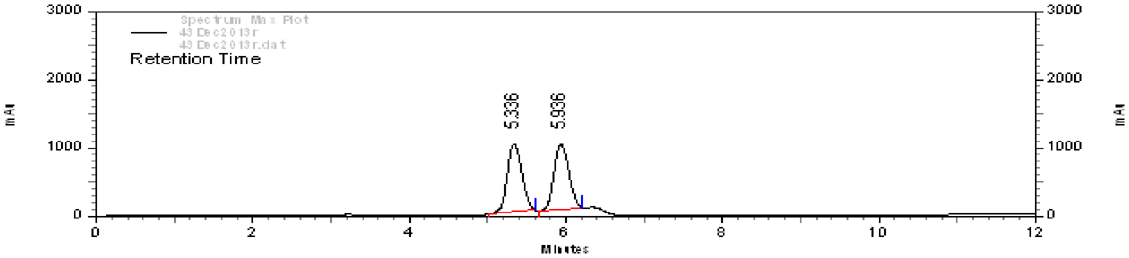

Spectrum Max Plot Results				
Retention Time	Area	Area Percent	Height	
5.336	12995113	50.494	996087	50.924
5.936	12740632	49.506	959956	49.076
Totals	25735745	100.000	1956043	100.000

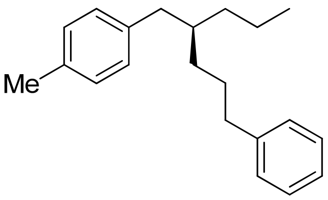

(*S*)-1-methyl-4-(5-phenyl-2-propylpentyl)benzene

86%; Colorless oil; $[\alpha]_D^{25}$ + 8.2 (*c* 1.0, $CHCl_3$, 78% ee); IR (neat) 3056, 3014, 1456, 989, 794; ^1H NMR ($CDCl_3$, 300 MHz): δ 7.29-6.99 (m, 9H), 2.57-2.46 (m, 4H), 2.32, (s, 3H), 1.68-1.23 (m, 9H), 0.87 (t, *J* = 6.4 Hz, 3H); ^{13}C NMR ($CDCl_3$, 75 MHz): δ 142.8, 138.4, 134.2, 128.9, 128.7, 128.3, 128.1, 125.5, 39.9, 39.3, 36.2, 35.4, 32.7, 28.5, 20.9, 19.6, 14.3; HRMS (APCI) calcd for $C_{21}H_{29}$ $[M+1]^+$: 281.2269; found: 281.2268; HPLC (Daicel Chiralcel OD-H, *i*-PrOH/hexane = 0/100, 1.0 mL/min, 190 nm) t1 = 11.5 min (minor), t2 = 14.3 min (major).

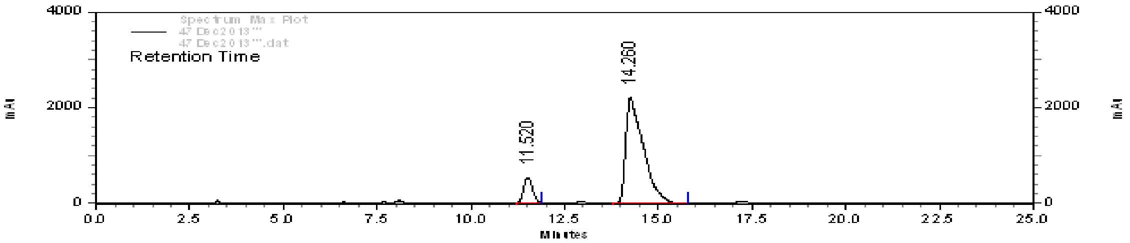

Spectrum Max Plot Results				
Retention Time	Area	Area Percent	Height	
11.520	8741593	10.887	530765	19.385
14.260	71551465	89.113	2207285	80.615
Totals				
	80293058	100.000	2738050	100.000

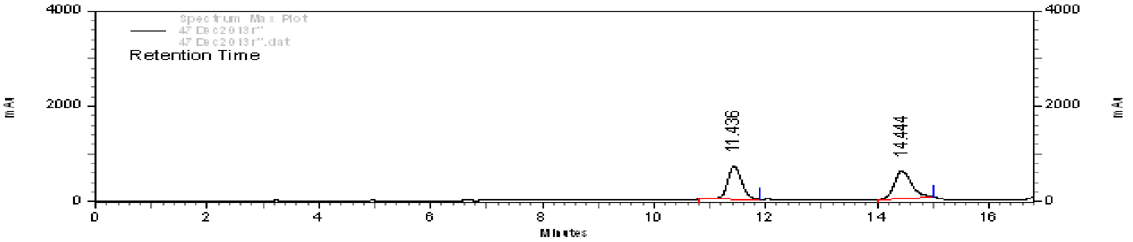

Spectrum Max Plot Results				
Retention Time	Area	Area Percent	Height	
11.436	11784640	49.315	691236	54.783
14.444	12111811	50.685	570537	45.217
Totals				
	23896451	100.000	1261773	100.000

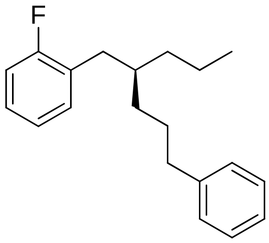

(*S*)-1-fluoro-2-(5-phenyl-2-propylpentyl)benzene

83%; Colorless oil; $[\alpha]_D^{25}$ -3.5 (*c* 1.0, $CHCl_3$, 72% ee); IR (neat) 3031, 3011, 1441, 793; ^{1}H NMR ($CDCl_3$, 200 MHz): δ 7.32-6.96 (m, 9H), 2.60-2.53 (m, 4H), 1.73-1.27 (m, 9H), 0.90 (t, *J* = 6.0 Hz, 3H); ^{13}C NMR ($CDCl_3$, 75 MHz): δ 131.4, 128.3(d, *J* = 11.3 Hz), 127.2 (d, *J* = 8.1 Hz), 125.4, 123.5, 115.2 (d, *J* = 23.3 Hz), 38.2, 36.1, 35.5, 33.5, 32.8, 28.3, 19.5, 14.3; ^{19}F NMR ($CDCl_3$, 188 MHz): δ -121.6; HRMS (APCI) calcd for $C_{20}H_{26}F$ $[M+1]^+$: 285.2019; found: 285.2009; HPLC (Daicel Chiralcel OJ-H, *i*-PrOH/hexane = 4/96, 1.0 mL/min, 190 nm) t1 = 4.3 min (minor), t2 = 5.6 min (major).

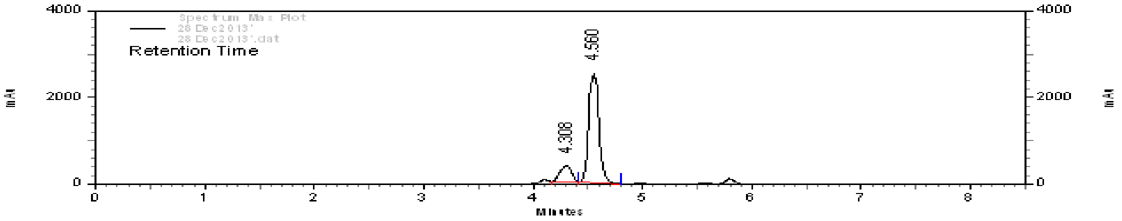

Spectrum Max Plot Results				
Retention Time	Area	Area Percent	Height	
4.308	2625446	13.801	364344	12.619
4.560	16397801	86.199	2522831	87.381
Totals				
	19023247	100.000	2887175	100.000

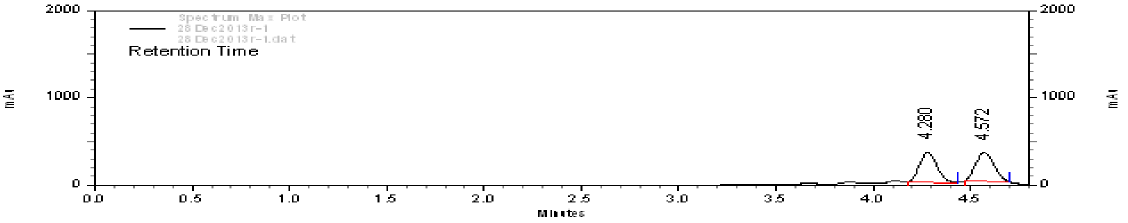

Spectrum Max Plot Results				
Retention Time	Area	Area Percent	Height	
4.280	2097213	48.762	343067	50.682
4.572	2203700	51.238	333838	49.318
Totals				
	4300913	100.000	676905	100.000

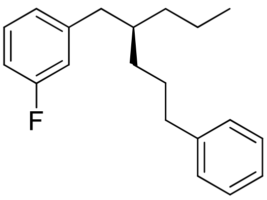

(*S*)-1-fluoro-3-(5-phenyl-2-propylpentyl)benzene

80%; Colorless oil; $[\alpha]_D^{25}$ + 3.0 (*c* 1.0, $CHCl_3$, 82% ee); IR (neat) 3028, 3009, 1492, 786; ^{1}H NMR ($CDCl_3$, 200 MHz): δ 7.28-6.82 (m, 9H), 2.57-2.43 (m, 4H), 1.70-1.25 (m, 9H), 0.90 (t, *J* = 6.0 Hz, 3H); ^{13}C NMR ($CDCl_3$, 75 MHz): δ 129.5 (d, *J* = 10 Hz), 128.4 (d, *J* = 5.1 Hz), 125.6, 124.9, 116.1 (d, *J* = 20.3 Hz), 112.7 (d, *J* = 25.0 Hz), 40.4, 39.3, 36.2, 35.4, 32.7, 28.5, 19.7, 14.4; ^{19}F NMR ($CDCl_3$, 188 MHz): δ -117.4; HRMS (APCI) calcd for $C_{20}H_{26}F$ $[M+1]^+$: 285.2019; found: 285.2020; HPLC (Daicel Chiralcel OD-H, *i*-PrOH/hexane = 0/100, 1.0 mL/min, 190 nm) t1 = 14.3 min (minor), t2 = 14.9 min (major).

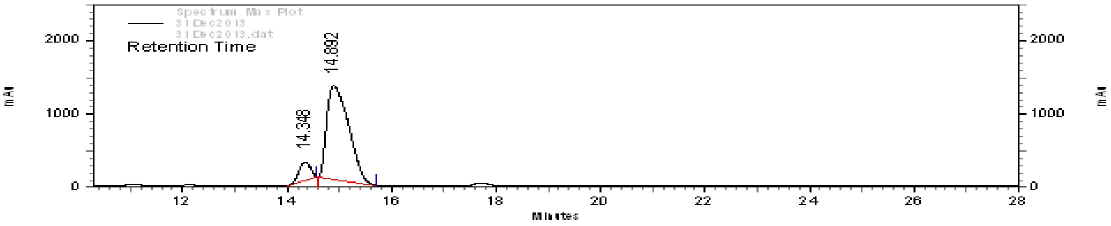

Spectrum Max Plot Results				
Retention Time	Area	Area Percent	Height	
14.348	3787793	9.151	249020	16.220
14.892	37604913	90.849	1286264	83.780
Totals	41392706	100.000	1535284	100.000

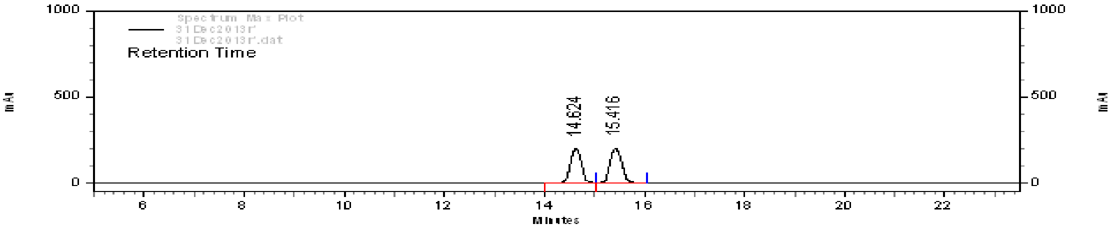

Spectrum Max Plot Results				
Retention Time	Area	Area Percent	Height	
14.624	3049876	48.071	197419	49.873
15.416	3294635	51.929	198428	50.127
Totals	6344511	100.000	395847	100.000

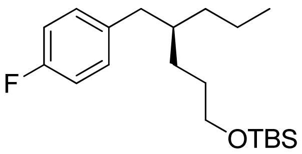

(*S*)-tert-butyl((4-(4-fluorobenzyl)heptyl)oxy)dimethylsilane

79%; Colorless oil; $[\alpha]_D^{25}$ - 5.6 (*c* 1.0, $CHCl_3$, 38% ee); IR (neat) 3021, 3018, 1487, 704; ^{1}H NMR ($CDCl_3$, 200 MHz): δ 7.13-6.91 (m, 4H), 3.59 (t, *J* = 6.0 Hz, 2H), 2.53-2.49 (m, 2H), 1.64-1.24 (m, 9H), 0.92 (m, 12H), 0 (s, 6H); ^{13}C NMR ($CDCl_3$, 50 MHz): δ 130.4 (d, *J* = 7.7 Hz), 115.0 (d, *J* = 20.8 Hz), 63.6, 39.7, 39.3, 35.4, 29.8, 29.0, 25.9, 19.7, 14.3, -5.2; ^{19}F NMR ($CDCl_3$, 188 MHz): δ -121.5; HRMS (APCI) calcd for $C_{20}H_{36}FOSi$ $[M+1]^+$: 339.2514; found: 339.2507. HPLC (Daicel Chiralcel OD-H, *i*-PrOH/hexane = 0.1/99.9, 1.0 mL/min, 190 nm) t1 = 4.4 min (minor), t2 = 4.9 min (major).

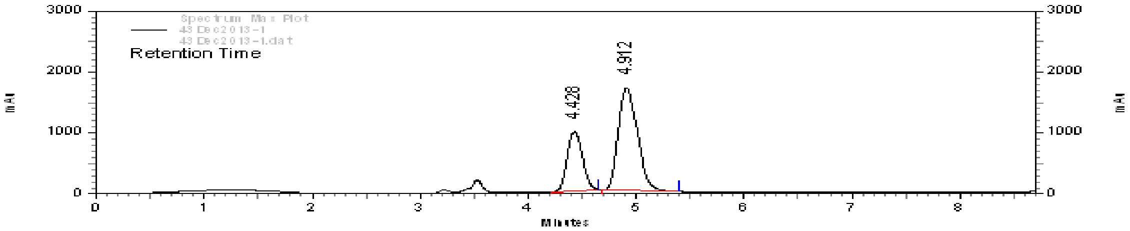

Spectrum Max Plot Results				
Retention Time	Area	Area Percent	Height	
4.428	9524812	31.243	963420	36.401
4.912	20961140	68.757	1683248	63.599
Totals				
	30485952	100.000	2646668	100.000

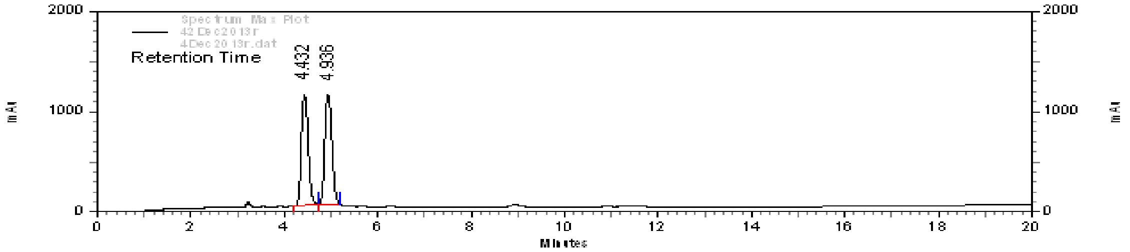

Spectrum Max Plot Results				
Retention Time	Area	Area Percent	Height	
4.432	11194952	48.774	1103869	50.337
4.936	11757519	51.226	1089071	49.663
Totals				
	22952471	100.000	2192940	100.000

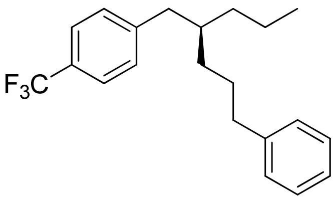

(*S*)-1-(5-phenyl-2-propylpentyl)-4-(trifluoromethyl)benzene

81%; Colorless oil; $[\alpha]_D^{25}$ -20.2 (*c* 1.0, $CHCl_3$, 89% ee); IR (neat) 3065, 3010, 1498, 766; ^{1}H NMR ($CDCl_3$, 300 MHz): δ 7.55-7.10 (m, 9H), 2.57-2.51 (m, 4H), 1.69-1.20 (m, 9H), 0.86 (t, *J* = 7.2 Hz, 3H); ^{13}C NMR ($CDCl_3$, 75 MHz): δ 145.7, 142.4, 136.4, 129.4 (d, *J* = 10.9 Hz),128.2 (d, *J* = 7.3 Hz), 126.0, 124.9 (q, *J* = 3.7 Hz), 40.2, 39.2, 36.0, 35.3, 32.4, 28.3, 19.5, 14.2 ; ^{19}F NMR ($CDCl_3$, 188 MHz): δ -65.5; HRMS (APCI) calcd for $C_{21}H_{25}F_3$ $[M]^+$: 334.1908; found: 334.1926. HPLC (Daicel Chiralcel OD-H, *i*-PrOH/hexane = 0/100, 1.0 mL/min, 190 nm) t1 = 9.0 min (minor), t2 = 11.0 min (major).

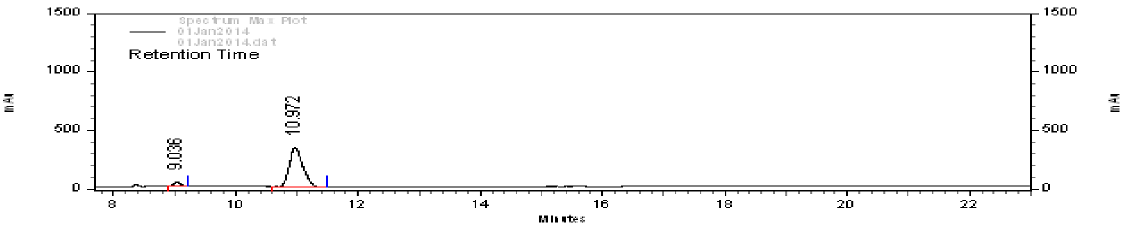

Spectrum Max Plot Results				
Retention Time	Area	Area Percent	Height	
9.036	303647	5.732	33566	9.191
10.972	4993843	94.268	331632	90.809
Totals				
	5297490	100.000	365198	100.000

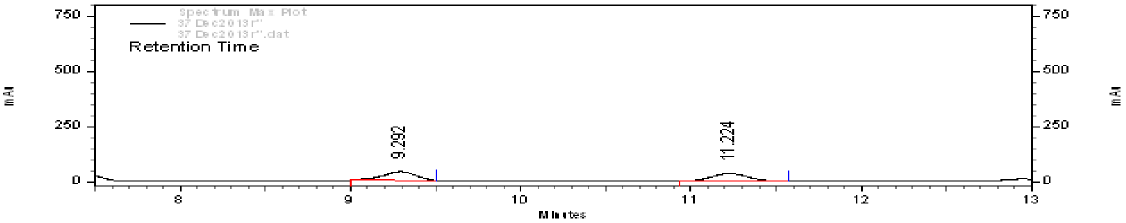

Spectrum Max Plot Results				
Retention Time	Area	Area Percent	Height	
9.292	539726	51.454	39415	53.565
11.224	509229	48.546	34169	46.435
Totals				
	1048955	100.000	73584	100.000

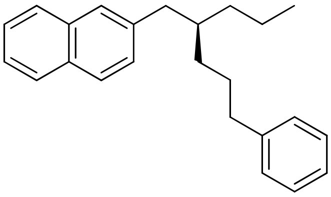

(*S*)-2-(5-phenyl-2-propylpentyl)naphthalene

58%; Yellow oil; $[\alpha]_D^{25}$ + 7.8 (*c* 1.0, $CHCl_3$, 71% ee); IR (neat) 3065, 3010, 1498, 766; ^{1}H NMR ($CDCl_3$, 200 MHz): δ 7.85-7.14 (m, 12H), 2.73-2.54 (m, 4H), 1.83-1.32 (m, 9H), 0.91 (t, J = 6.0 Hz, 3H); ^{13}C NMR ($CDCl_3$, 50 MHz): δ 142.8, 139.3, 133.5, 131.9, 128.4, 128.2, 128.0, 127.6, 127.4, 127.3, 125.8, 125.6, 125.0, 40.7, 39.4, 36.3, 35.6, 32.9, 28.6, 19.8, 14.5; HRMS (APCI) calcd for $C_{24}H_{28}$ $[M]^+$: 316.2191; found: 316.2166; HPLC (Daicel Chiralcel OD-H, *i*-PrOH/hexane = 4/96, 1.0 mL/min, 190 nm) t1 = 5.0 min (minor), t2 = 6.8 min (major).

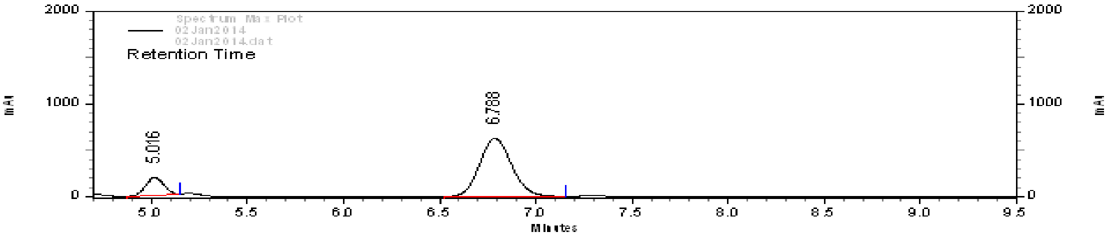

Spectrum Max Plot Results				
Retention Time	Area	Area Percent	Height	
5.016	1234119	14.541	188269	23.149
6.788	7252974	85.459	625023	76.851
Totals	8487093	100.000	813292	100.000

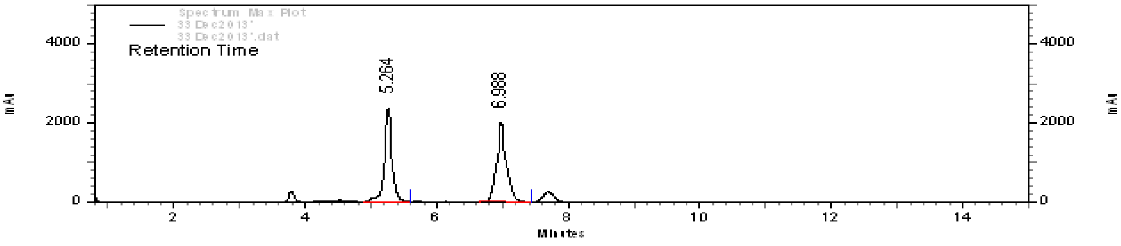

Spectrum Max Plot Results				
Retention Time	Area	Area Percent	Height	
5.264	18870726	48.232	2377895	54.265
6.988	20254033	51.768	2004106	45.735
Totals	39124759	100.000	4382001	100.000

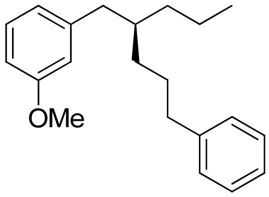

(S)-1-methoxy-3-(5-phenyl-2-propylpentyl)benzene

78%; Colorless oil; $[\alpha]_D^{25}$ + 4.4 (*c* 1.0, $CHCl_3$, 88% ee); IR (neat) 3045, 3020, 1457, 792; ^{1}H NMR ($CDCl_3$, 300 MHz): δ 7.27-6.6 (m, 9H), 3.76 (s, 3H), 2.53-2.50 (m, 4H), 1.80-1.24 (m, 9H), 0.89 (t, *J* = 6.0 Hz, 3H); ^{13}C NMR ($CDCl_3$, 50 MHz): δ 157.8, 140.3, 129.8, 128.3, 128.1, 125.5, 116.9, 112.3, 55.3, 38.5, 37.4, 36.1, 35.5, 32.8, 28.4, 19.5, 14.3; HRMS (APCI) calcd for $C_{21}H_{29}O$ $[M+1]^+$: 297.2218; found: 297.2210. HPLC (Daicel Chiralcel OD-H, *i*-PrOH/hexane = 0/100, 1.0 mL/min, 190 nm) t1 = 29.6 min (minor), t2 = 30.9 min (major).

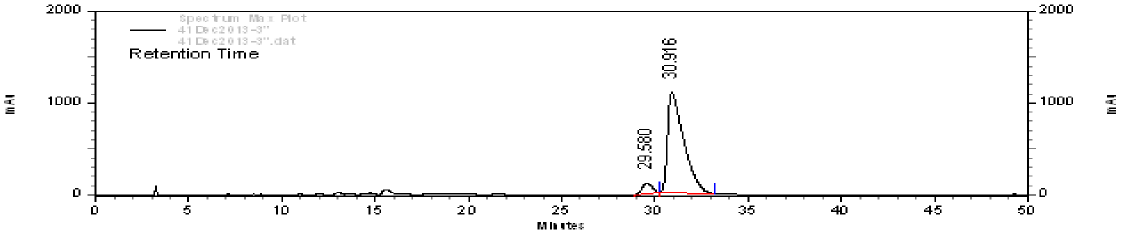

Spectrum Max Plot Results				
Retention Time	Area	Area Percent	Height	
29.580	4228575	5.983	114326	9.467
30.916	66453318	94.017	1093342	90.533
Totals	70681893	100.000	1207668	100.000

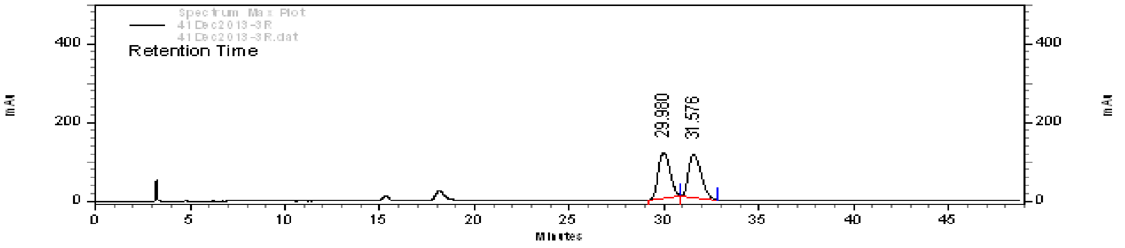

Spectrum Max Plot Results				
Retention Time	Area	Area Percent	Height	
29.980	4896767	49.593	116079	51.515
31.576	4977156	50.407	109251	48.485
Totals	9873923	100.000	225330	100.000

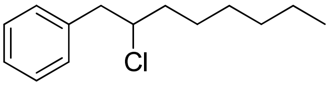

(2-chlorooctyl)benzene

90%; Colorless oil; IR (neat) 3019, 1416, 783, 405; ^{1}H NMR ($CDCl_3$, 300 MHz): δ 7.19-7.13 (m, 5H), 4.35-4.20 (m, 1H), 3.36-3.12 (m, 2H), 1.81-1.55 (m, 3H), 1.40-1.25 (m, 7H), 0.88 (t, J = 6.8 Hz 3H); ^{13}C NMR ($CDCl_3$, 100 MHz): 139.7, 128.9, 128.3, 126.6, 47.4, 39.4, 38.9, 31.5, 29.5, 28.3, 22.5, 13.9; HRMS (APCI) calcd for $C_{14}H_{21}$ $[M\text{-}Cl]^{+}$: 189.1643; found: 189.1628; HPLC (Daicel Chiralcel OD-H, *i*-PrOH/hexane = 0.2/99.8, 1.0 mL/min, 190 nm) t1 = 7.3 min,t2 = 7.7 min (racemic).

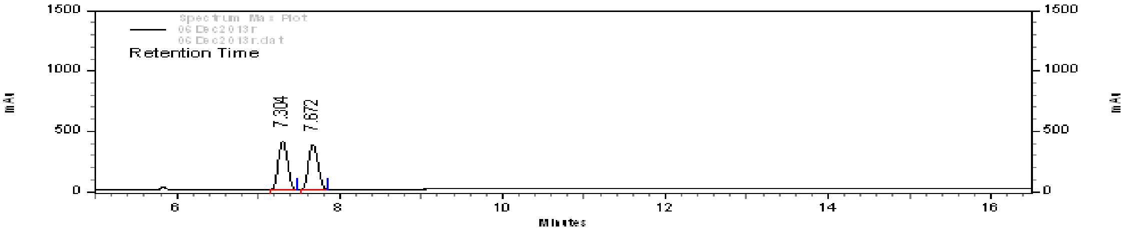

Spectrum Max Plot Results				
Retention Time	Area	Area Percent	Height	
7.304	3073510	50.633	399278	52.028
7.672	2996657	49.367	368150	47.972
Totals				
	6070167	100.000	767428	100.000

References

1. Kulbitski, K., Nisnevich, G. & Gandelman, M. Metal-free efficient, general and facile iododecarboxylation method with biodegradable co-products. *Adv. Synth. Catal.* **353**, 1438-1442 (2011).

2. Saito, B. & Fu, G. C. Enantioselective alkyl-alkyl Suzuki cross-couplings of unactivated homobenzylic halides. *J. Am. Chem. Soc.* **130**, 6694-6695 (2008).

^1H/^{13}C/^{19}F Spectra

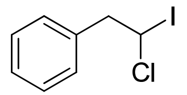

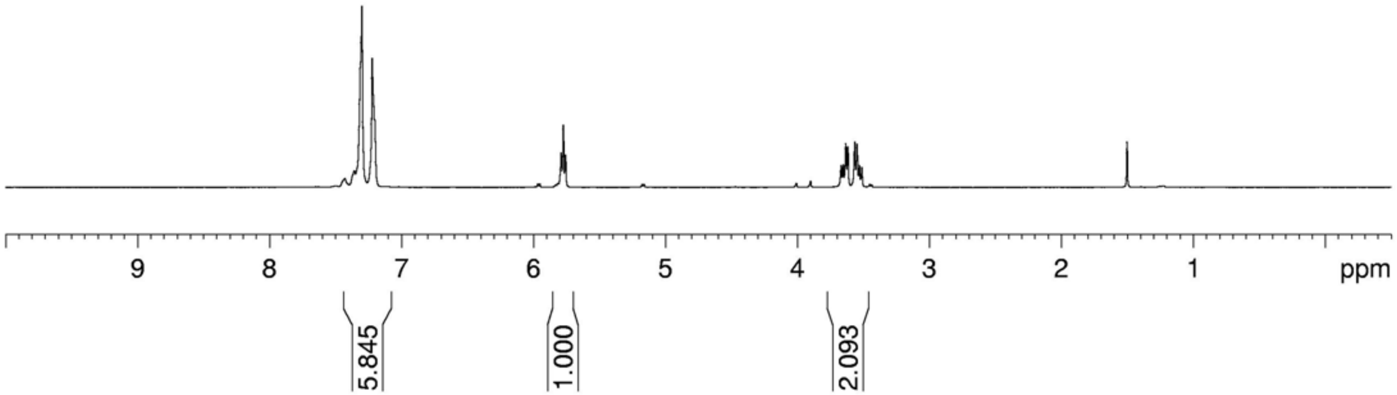

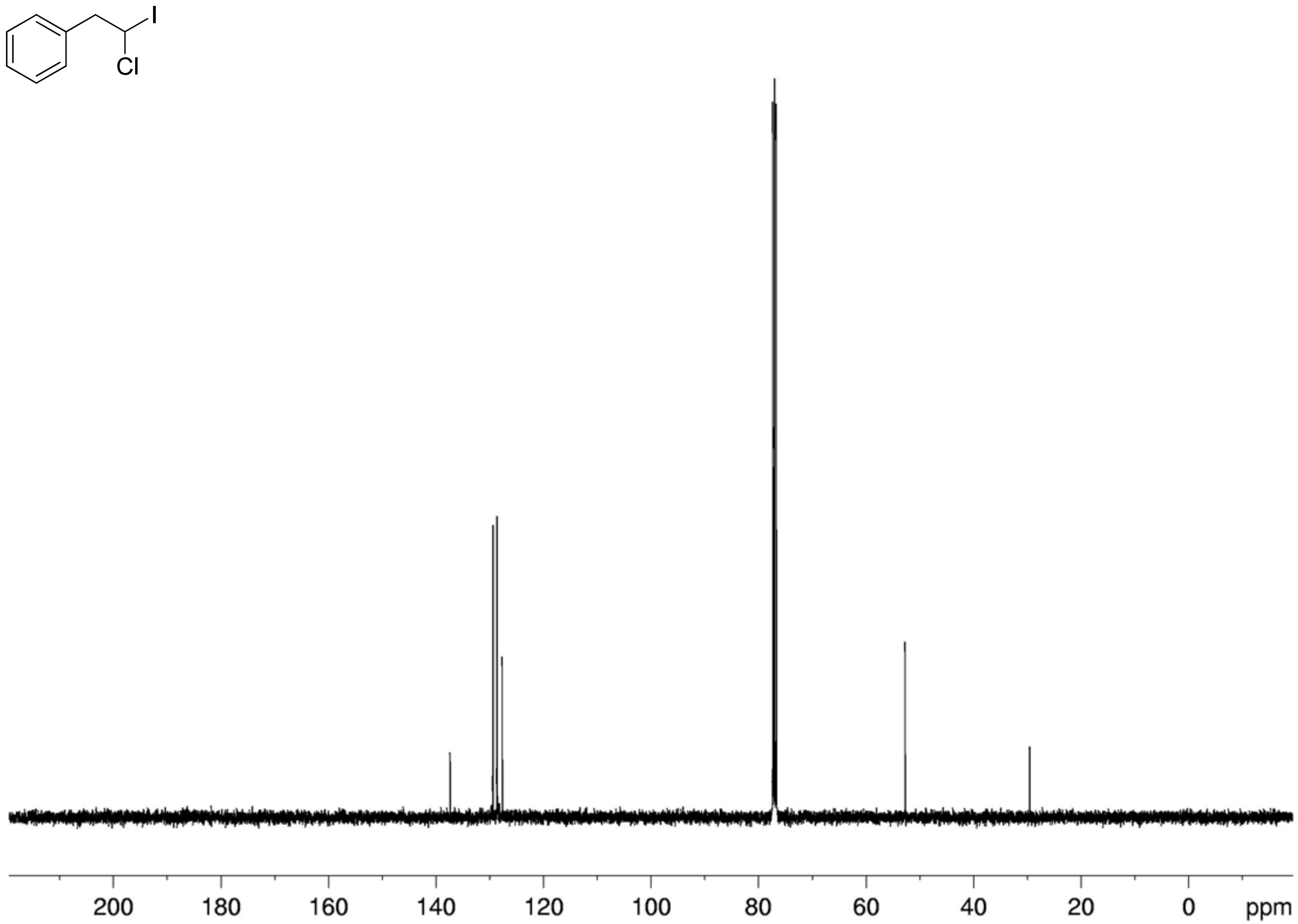
I
Cl
200
180
160
140
120
100
80
60
40
20
0
ppm

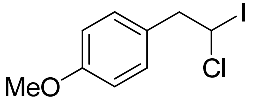

I
Cl
MeO

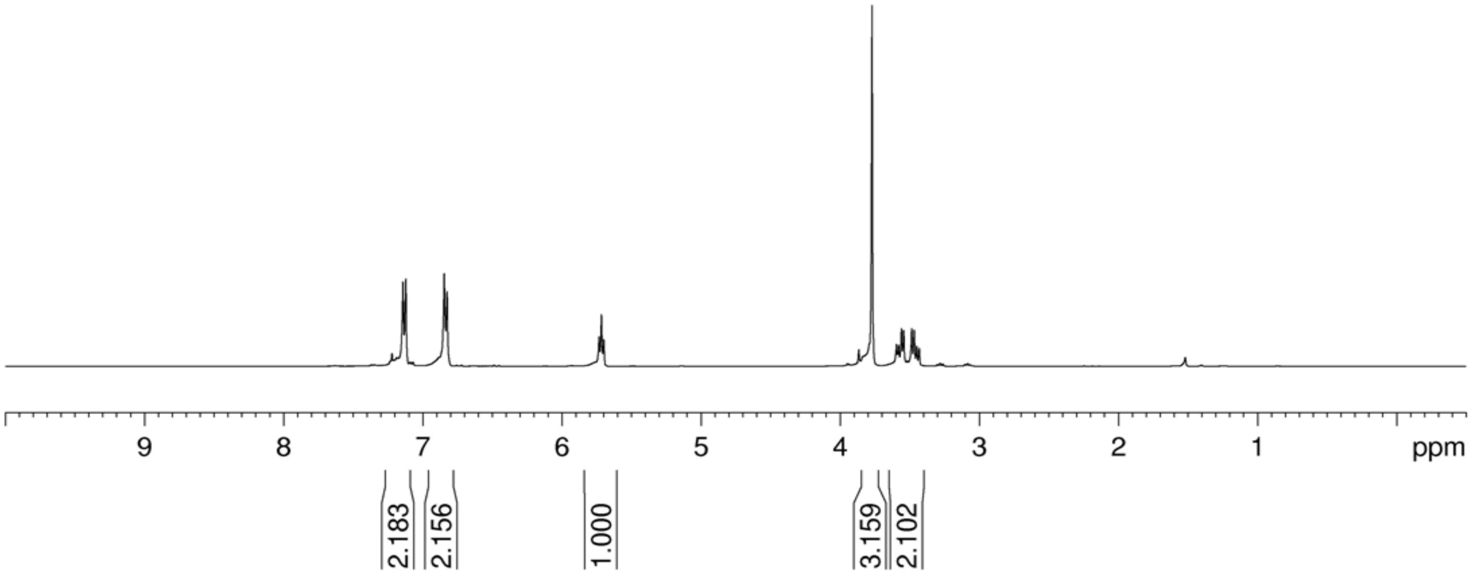

9
8
7
6
5
4
3
2
1
ppm
2.183
2.156
1.000
3.159
2.102

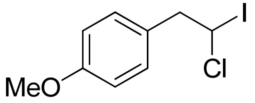

MeO
Cl
I

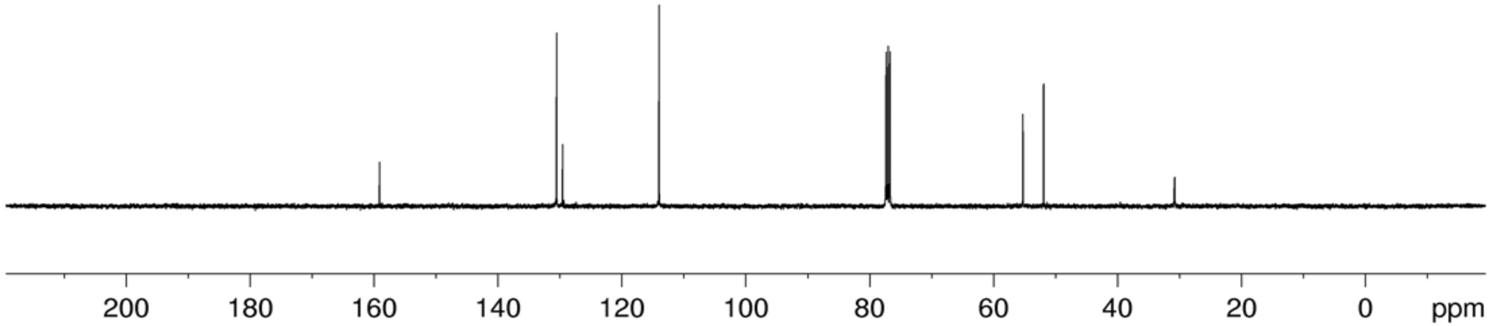

200
180
160
140
120
100
80
60
40
20
0
ppm

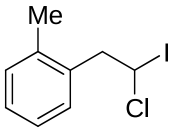
Me
I
Cl

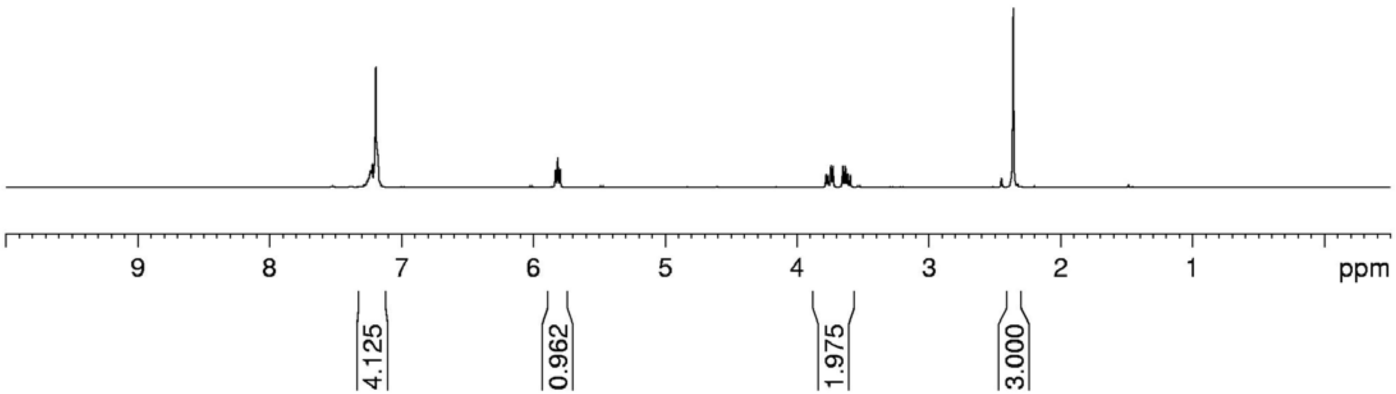
9
8
7
6
5
4
3
2
1
ppm
4.125
0.962
1.975
3.000

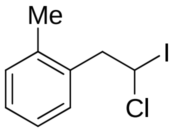
Me
I
Cl

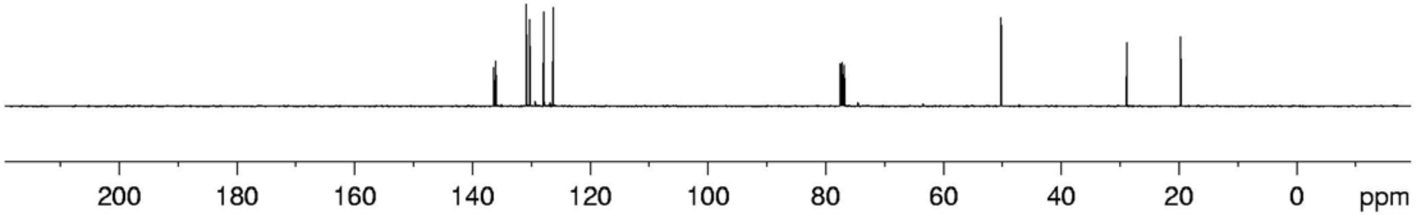
200
180
160
140
120
100
80
60
40
20
0
ppm

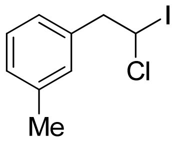
I
Cl
Me

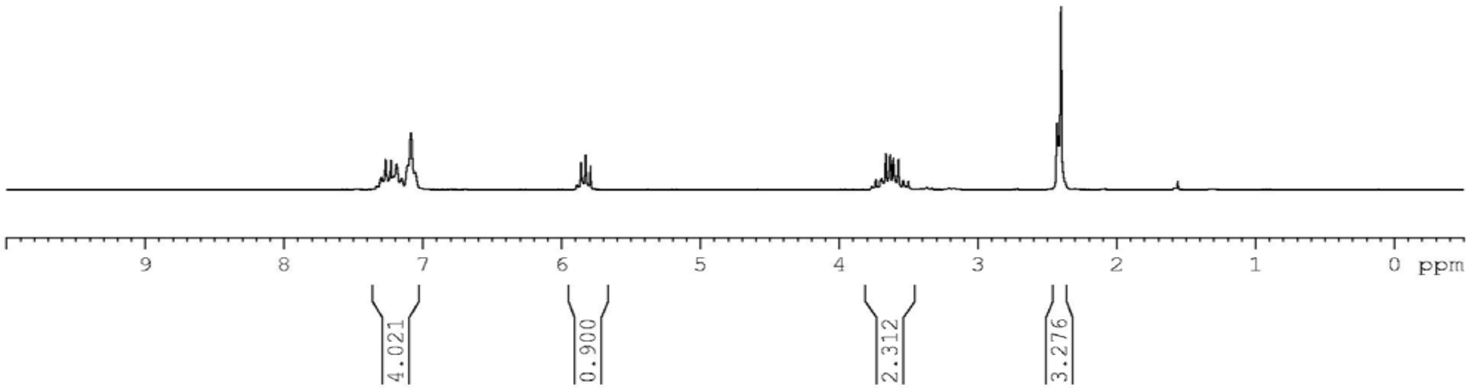
9
8
7
6
5
4
3
2
1
0 ppm
4.021
0.900
2.312
3.276

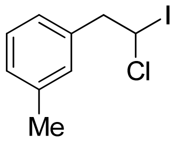

I
Cl
Me

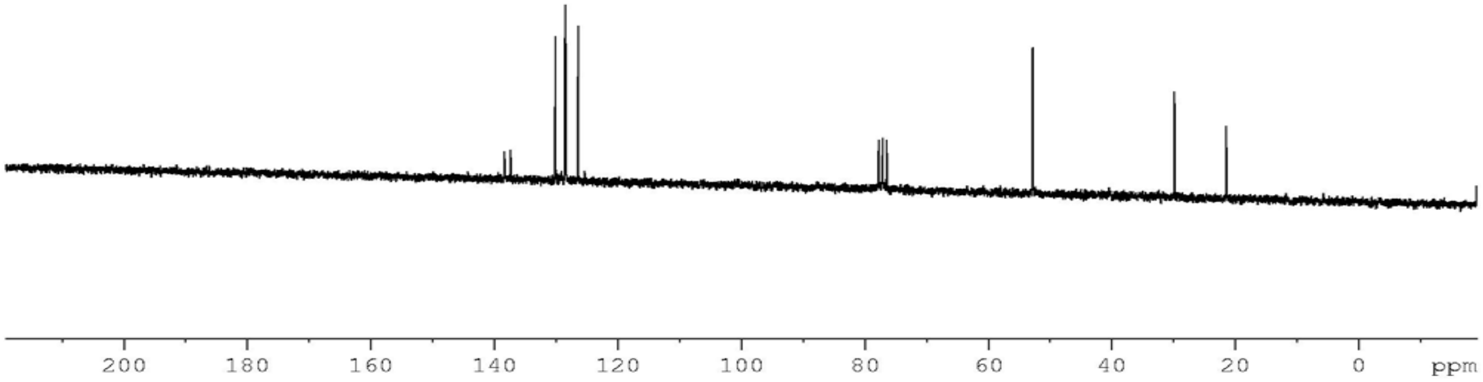

200
180
160
140
120
100
80
60
40
20
0
ppm

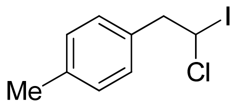

I
Cl
Me

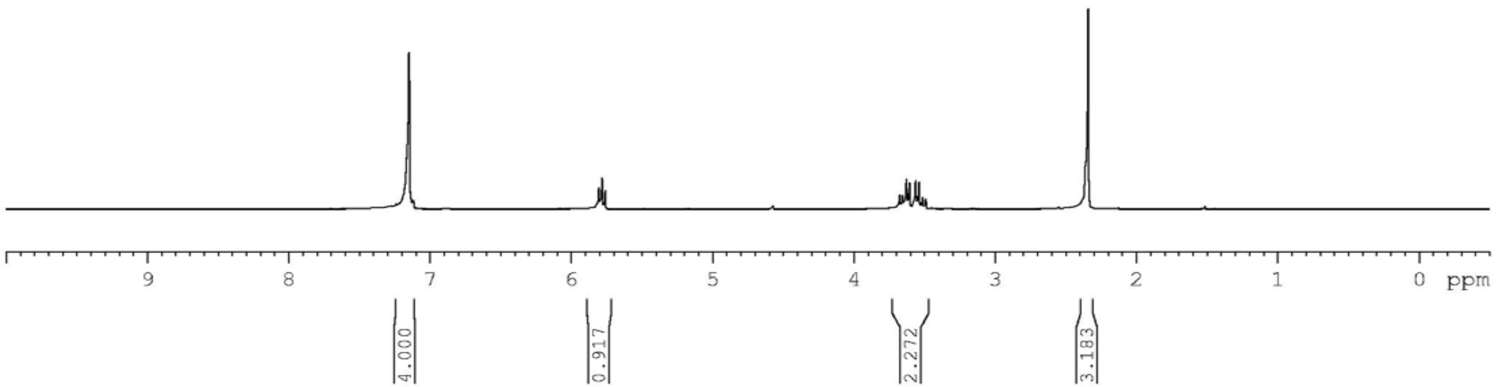

9
8
7
6
5
4
3
2
1
0
ppm
4.000
0.917
2.272
3.183

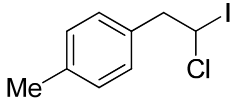
I
Cl
Me

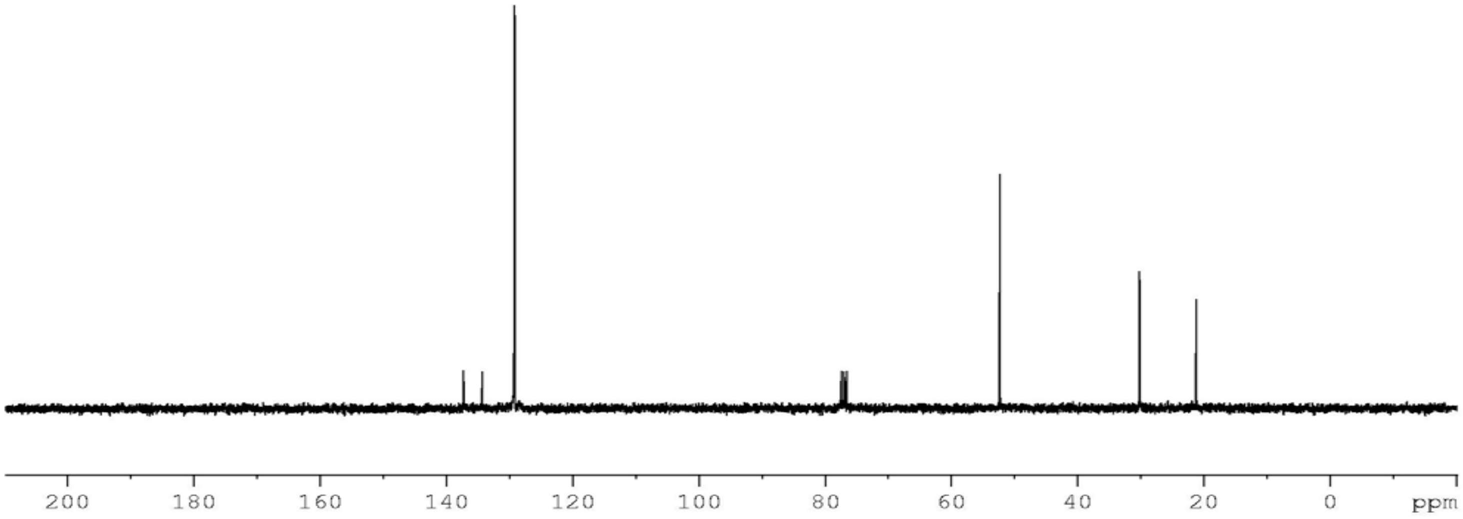
200
180
160
140
120
100
80
60
40
20
0
ppm

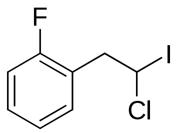
F
I
Cl

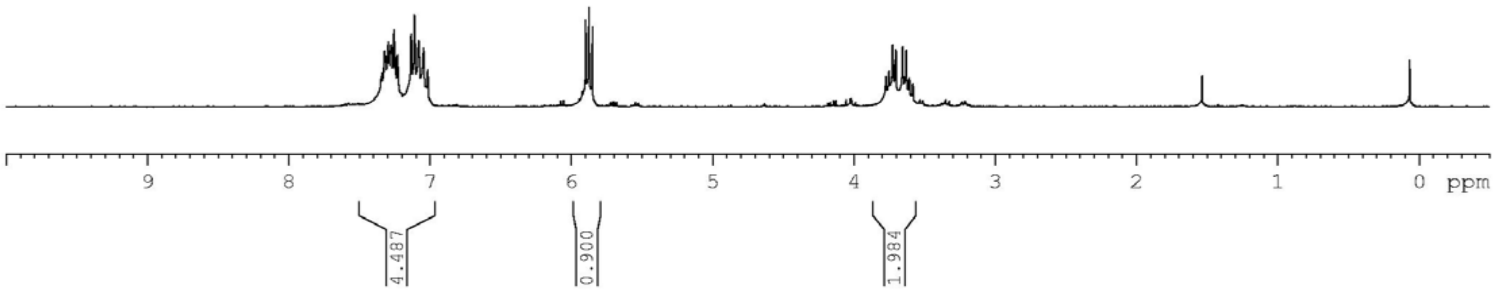
9
8
7
6
5
4
3
2
1
0
ppm
4.487
0.900
1.984

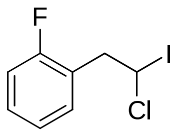
F
I
Cl

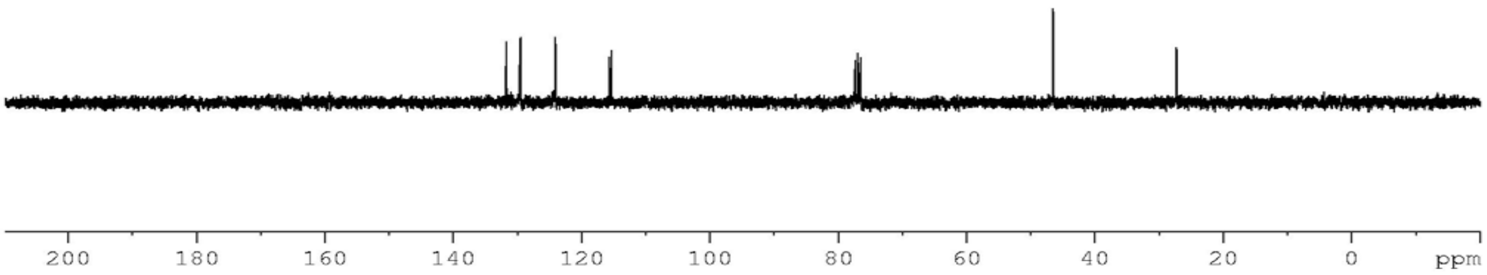
200
180
160
140
120
100
80
60
40
20
0
ppm

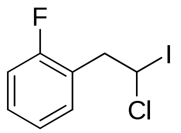
F
I
Cl

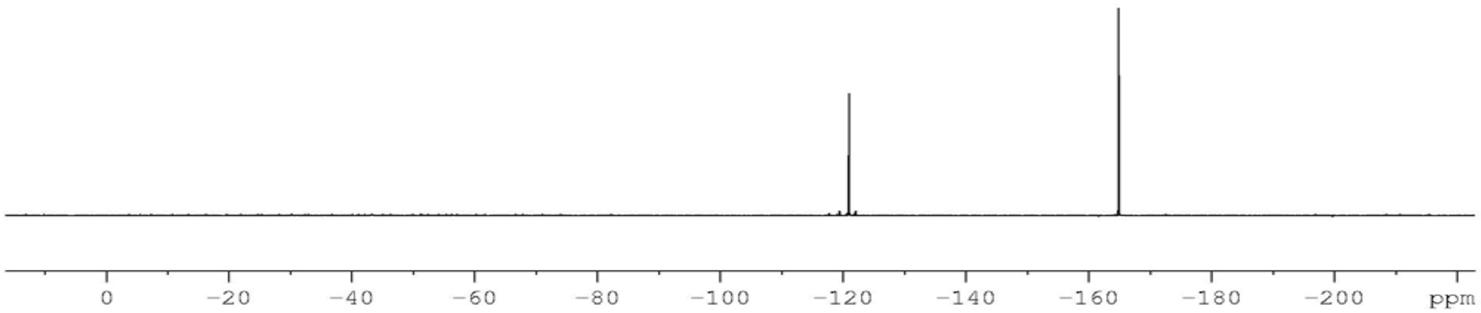
0
−20
−40
−60
−80
−100
−120
−140
−160
−180
−200
ppm

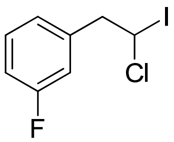
I
Cl
F

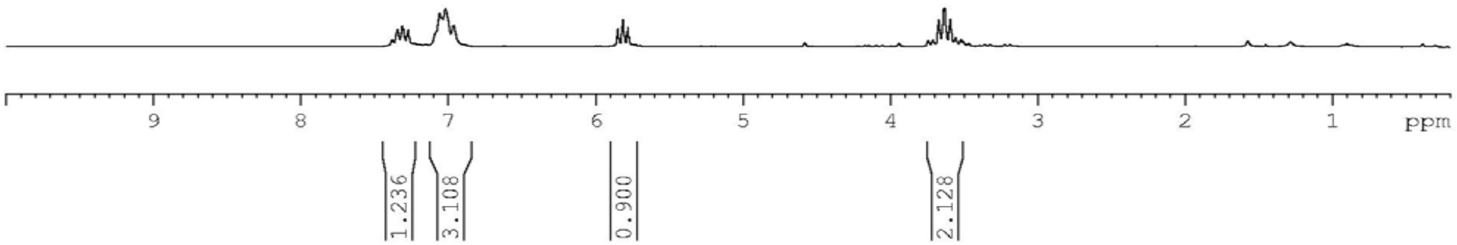
9
8
7
6
5
4
3
2
1
ppm
1.236
3.108
0.900
2.128

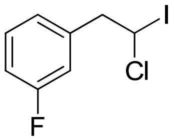
I
Cl
F

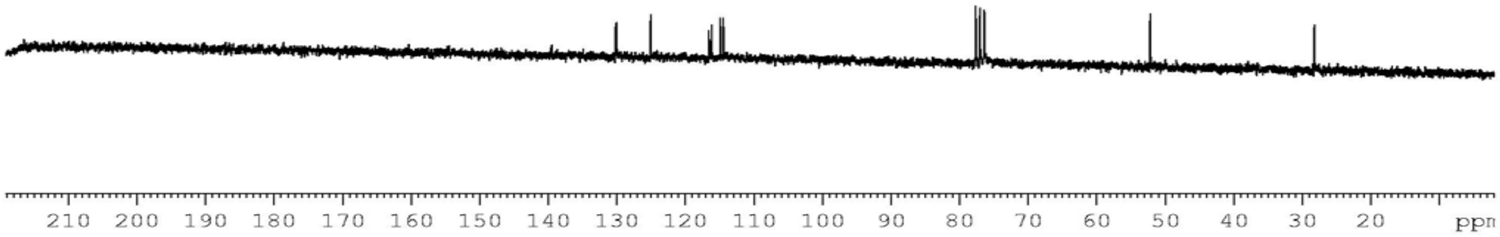
210 200 190 180 170 160 150 140 130 120 110 100 90 80 70 60 50 40 30 20 ppm

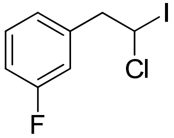
I
Cl
F

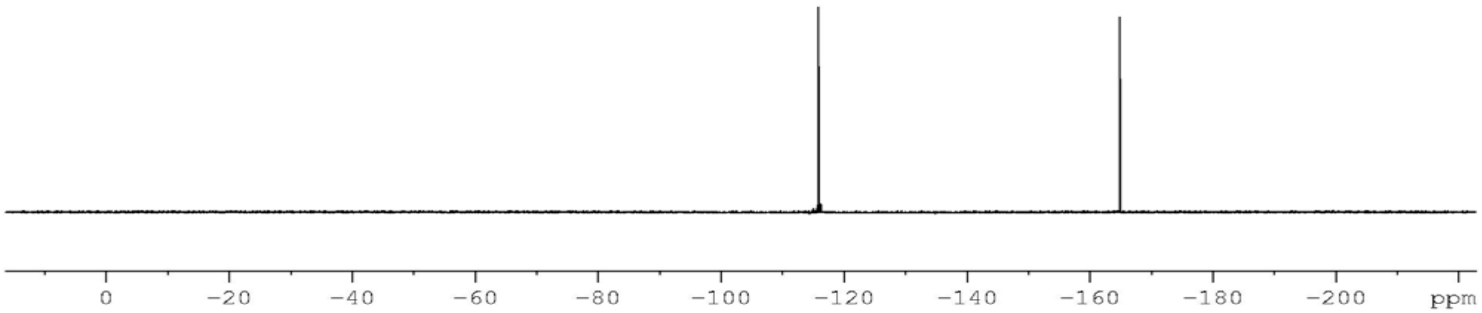
0
−20
−40
−60
−80
−100
−120
−140
−160
−180
−200
ppm

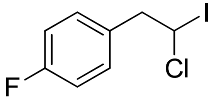

I
Cl
F

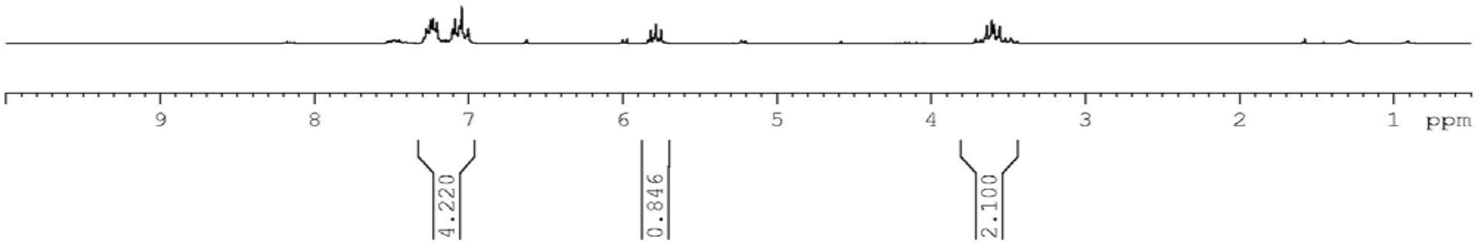

9
8
7
6
5
4
3
2
1
ppm
4.220
0.846
2.100

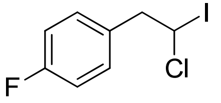

I
Cl
F

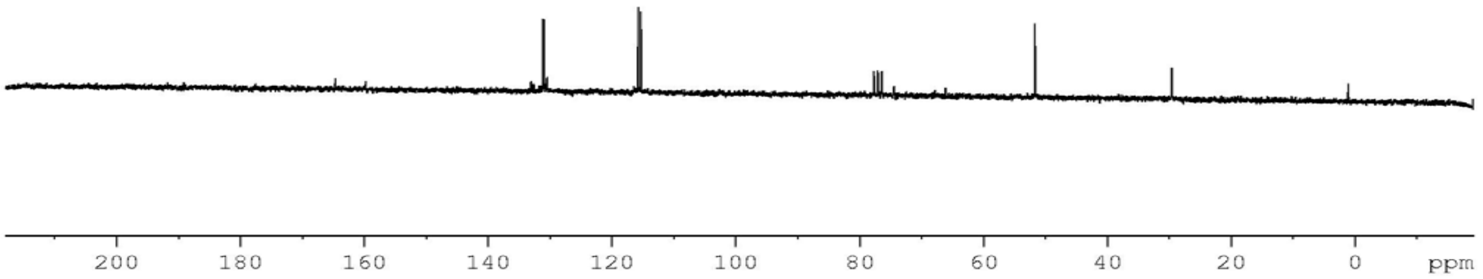

200
180
160
140
120
100
80
60
40
20
0
ppm

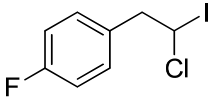
I
Cl
F

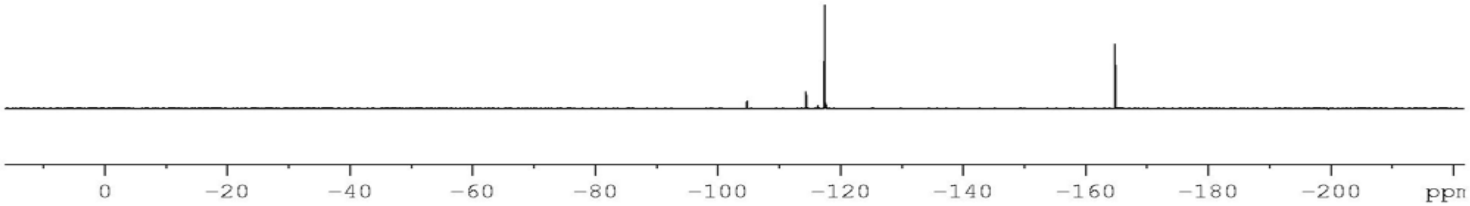
0
−20
−40
−60
−80
−100
−120
−140
−160
−180
−200
ppm

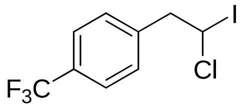
I
Cl
F3C

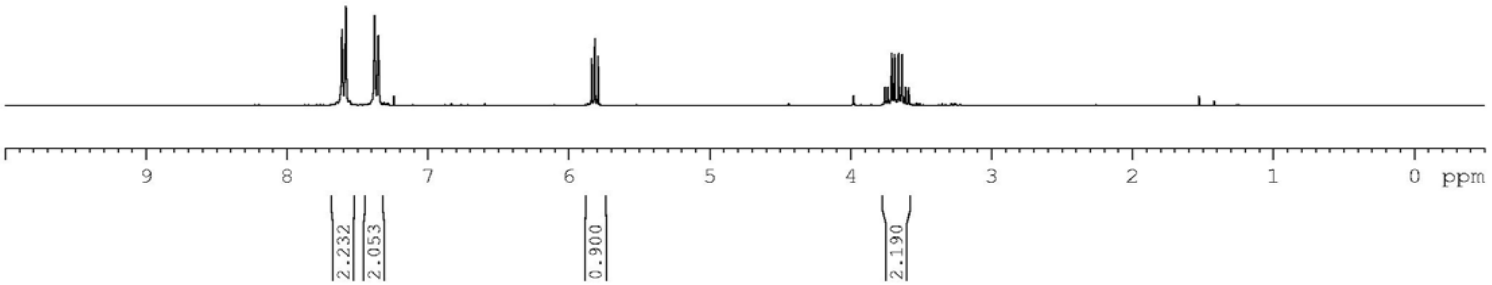
9
8
7
6
5
4
3
2
1
0 ppm
2.232
2.053
0.900
2.190

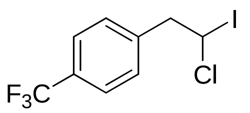
I
Cl
F3C

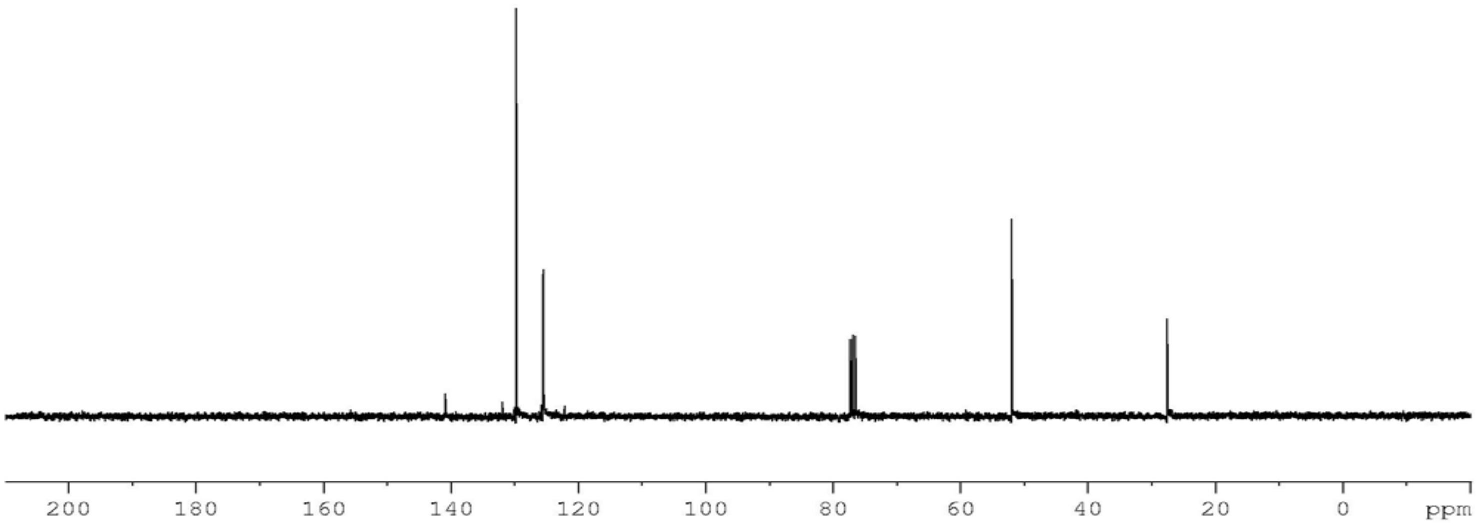
200
180
160
140
120
100
80
60
40
20
0
ppm

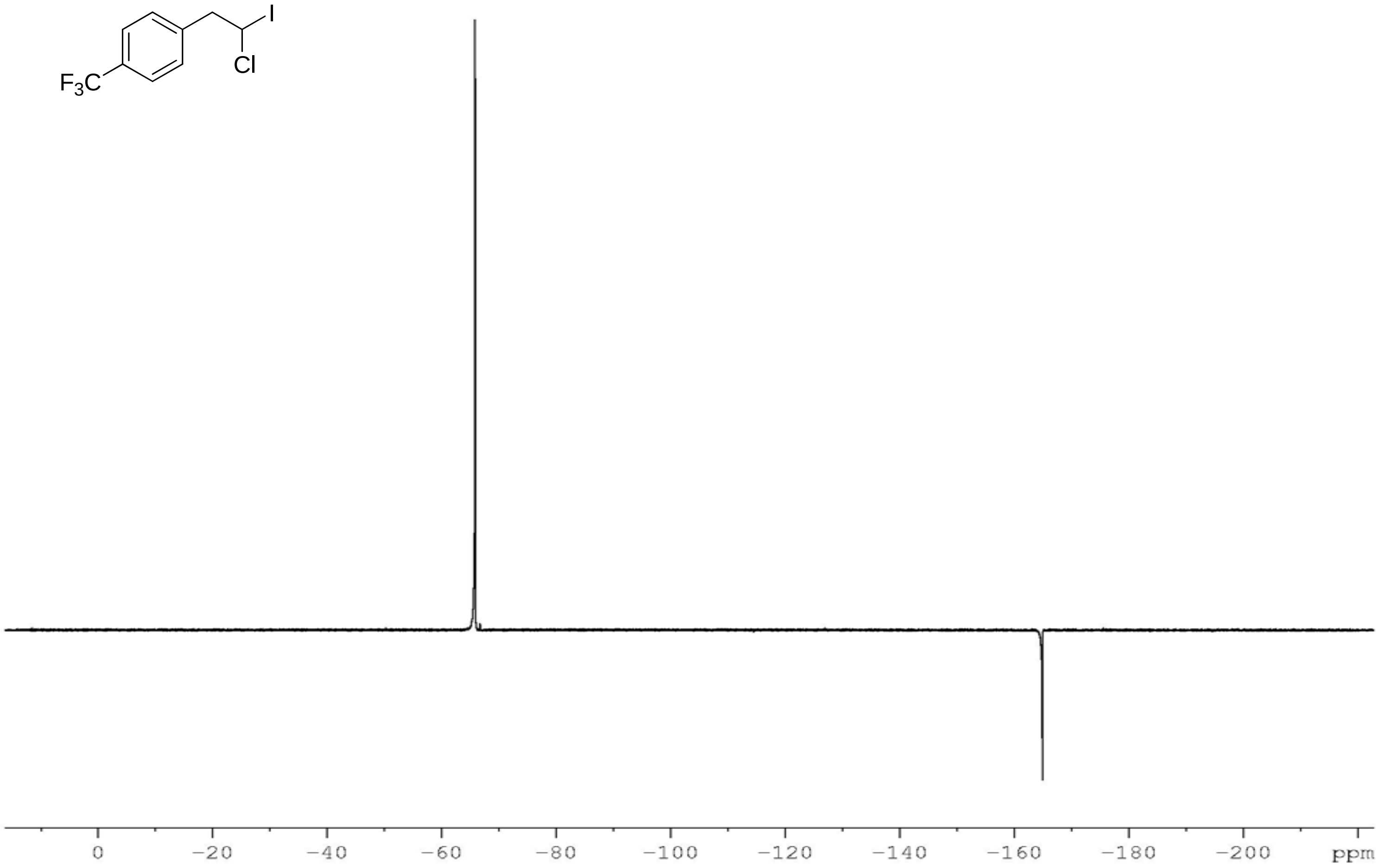
I
Cl
F3C
0
−20
−40
−60
−80
−100
−120
−140
−160
−180
−200
ppm

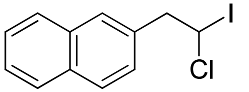
I
Cl

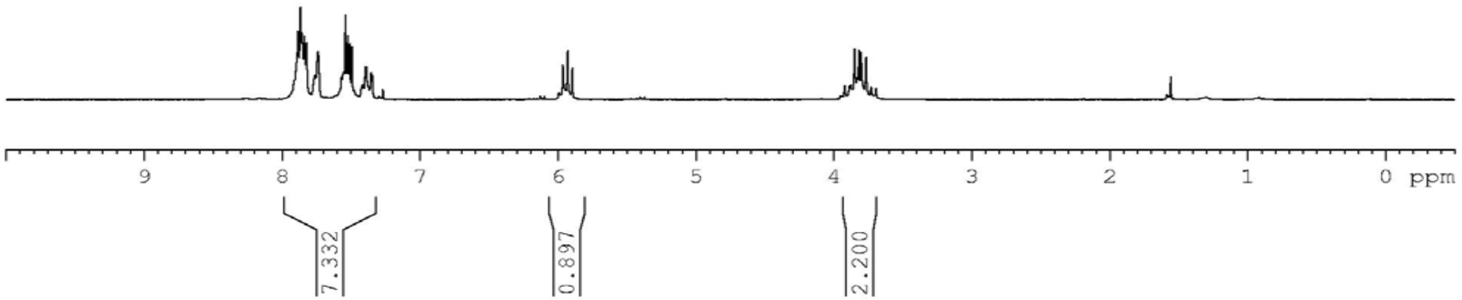
9
8
7
6
5
4
3
2
1
0 ppm
7.332
0.897
2.200

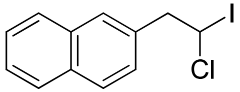
I
Cl

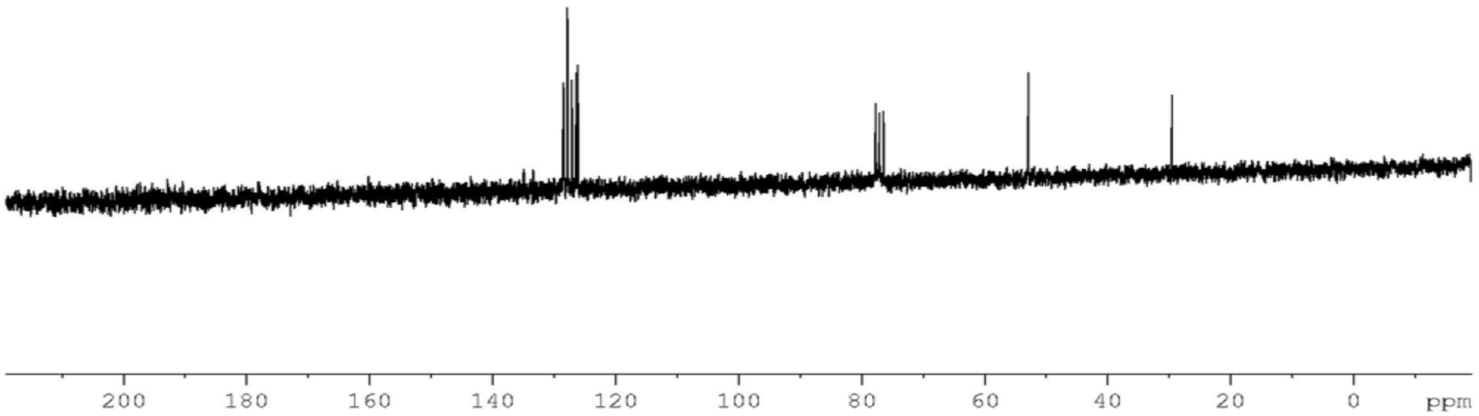
200
180
160
140
120
100
80
60
40
20
0
ppm

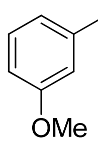
I
Cl
OMe

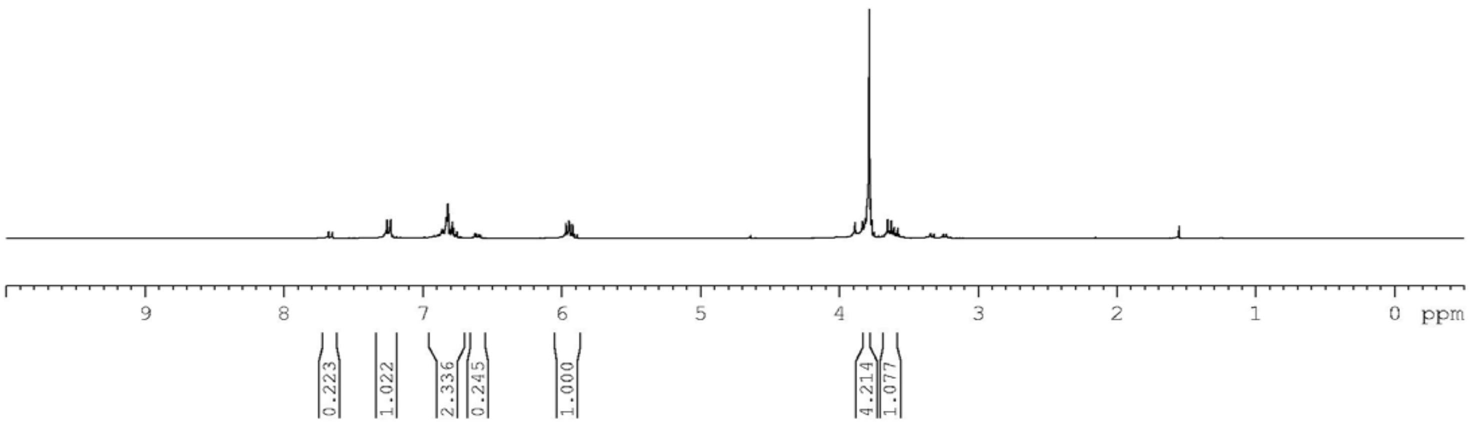
9
8
7
6
5
4
3
2
1
0
ppm
0.223
1.022
2.336
0.245
1.000
4.214
1.077

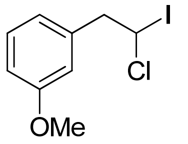

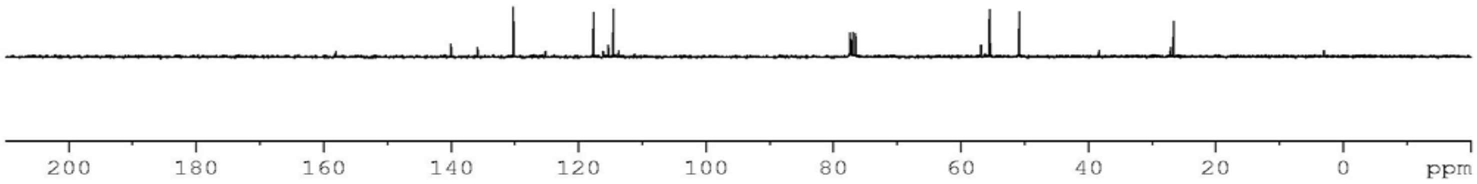
200
180
160
140
120
100
80
60
40
20
0
ppm

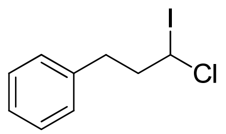
I
Cl

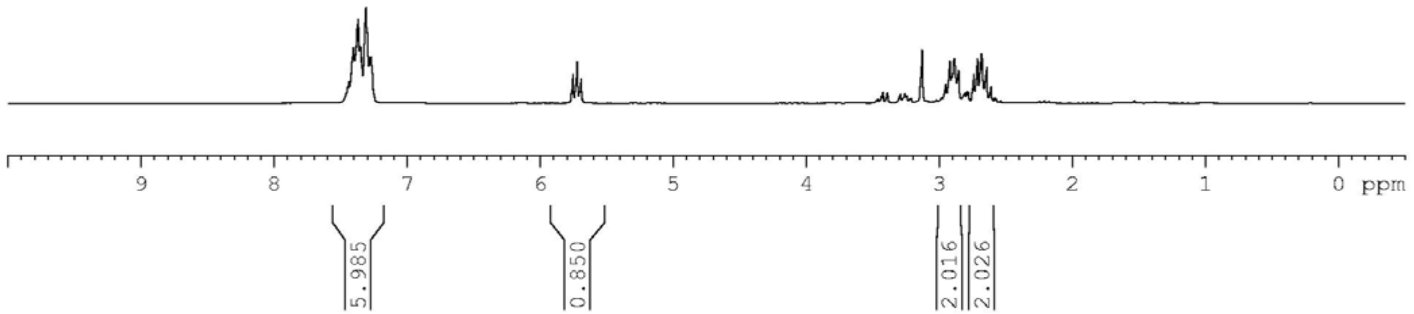
9
8
7
6
5
4
3
2
1
0 ppm
5.985
0.850
2.016
2.026

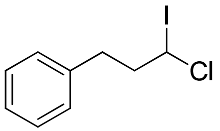

I
Cl

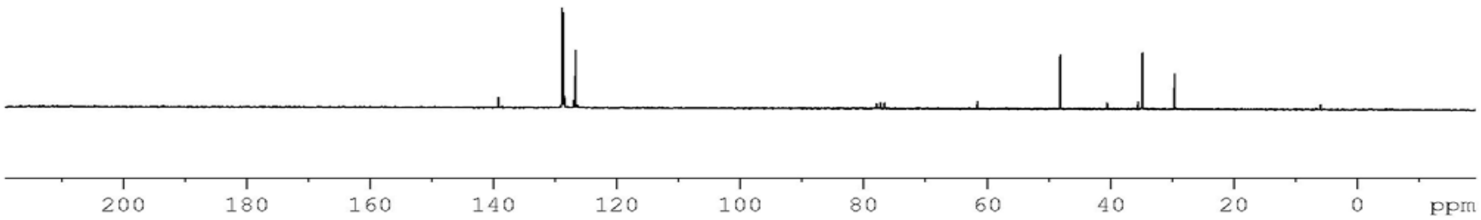

200
180
160
140
120
100
80
60
40
20
0
ppm

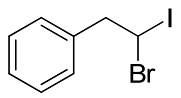
I
Br

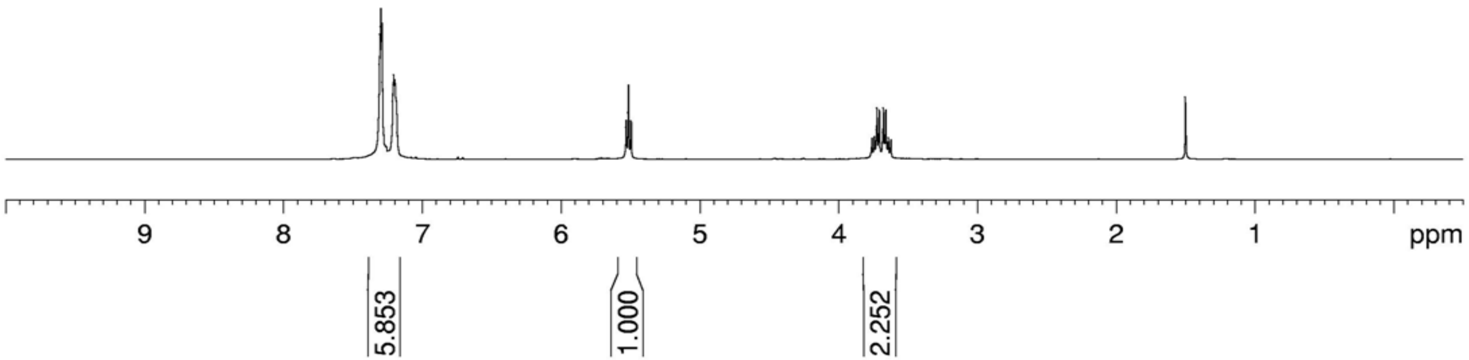
9
8
7
6
5
4
3
2
1
ppm
5.853
1.000
2.252

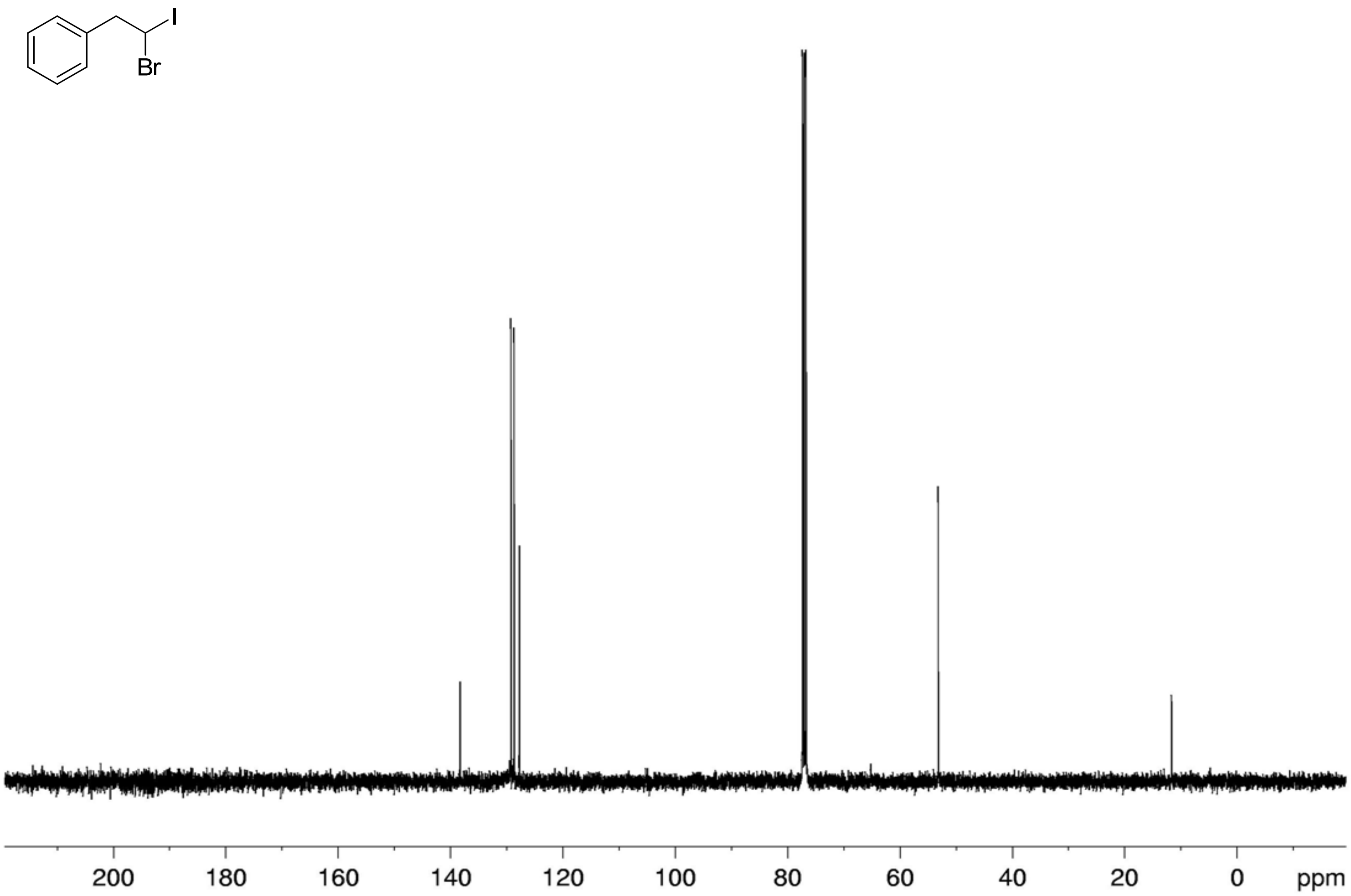
I
Br
200
180
160
140
120
100
80
60
40
20
0
ppm

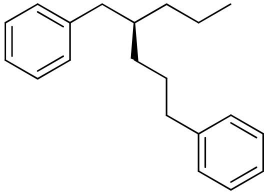

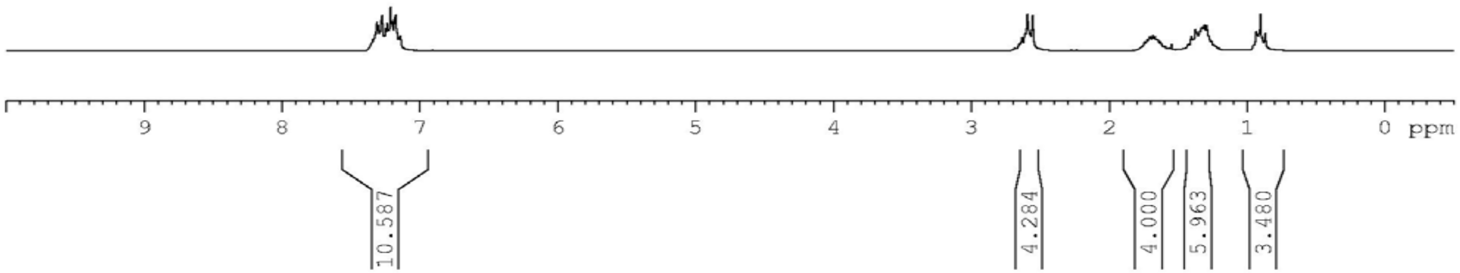
9
8
7
6
5
4
3
2
1
0 ppm
10.587
4.284
4.000
5.963
3.480

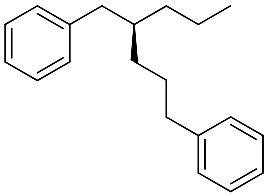

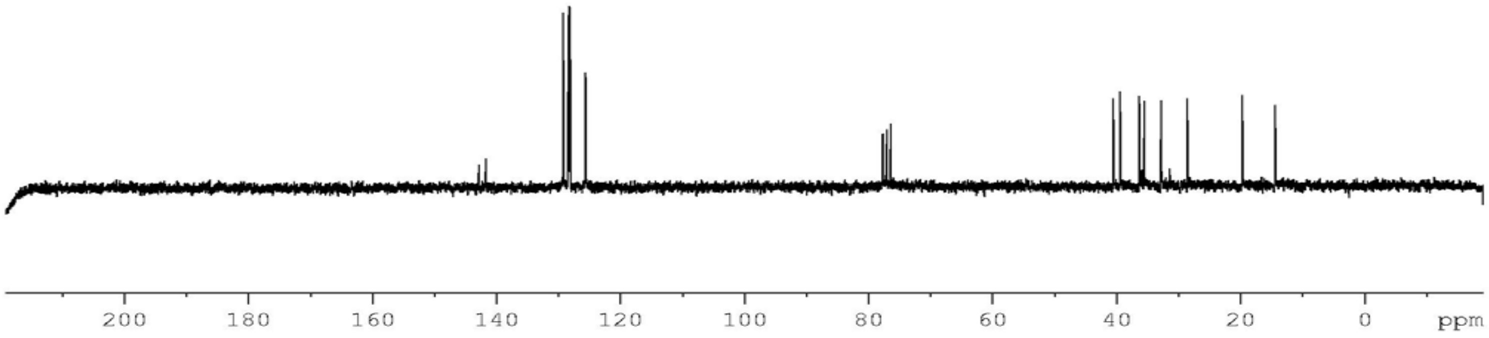

200
180
160
140
120
100
80
60
40
20
0
ppm

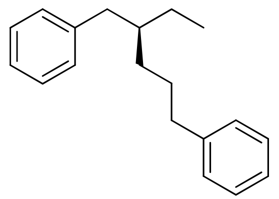

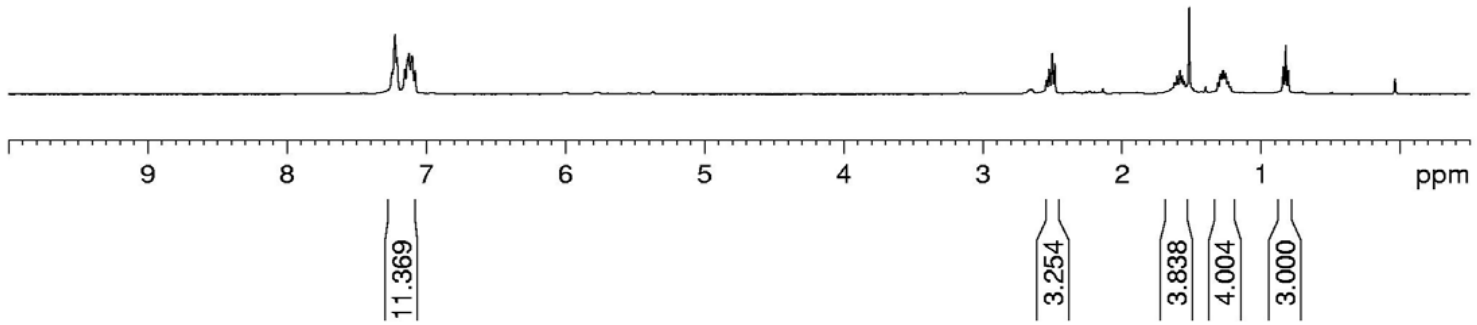

9
8
7
6
5
4
3
2
1
ppm
11.369
3.254
3.838
4.004
3.000

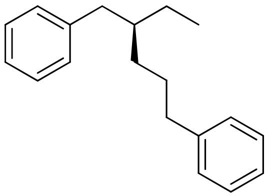

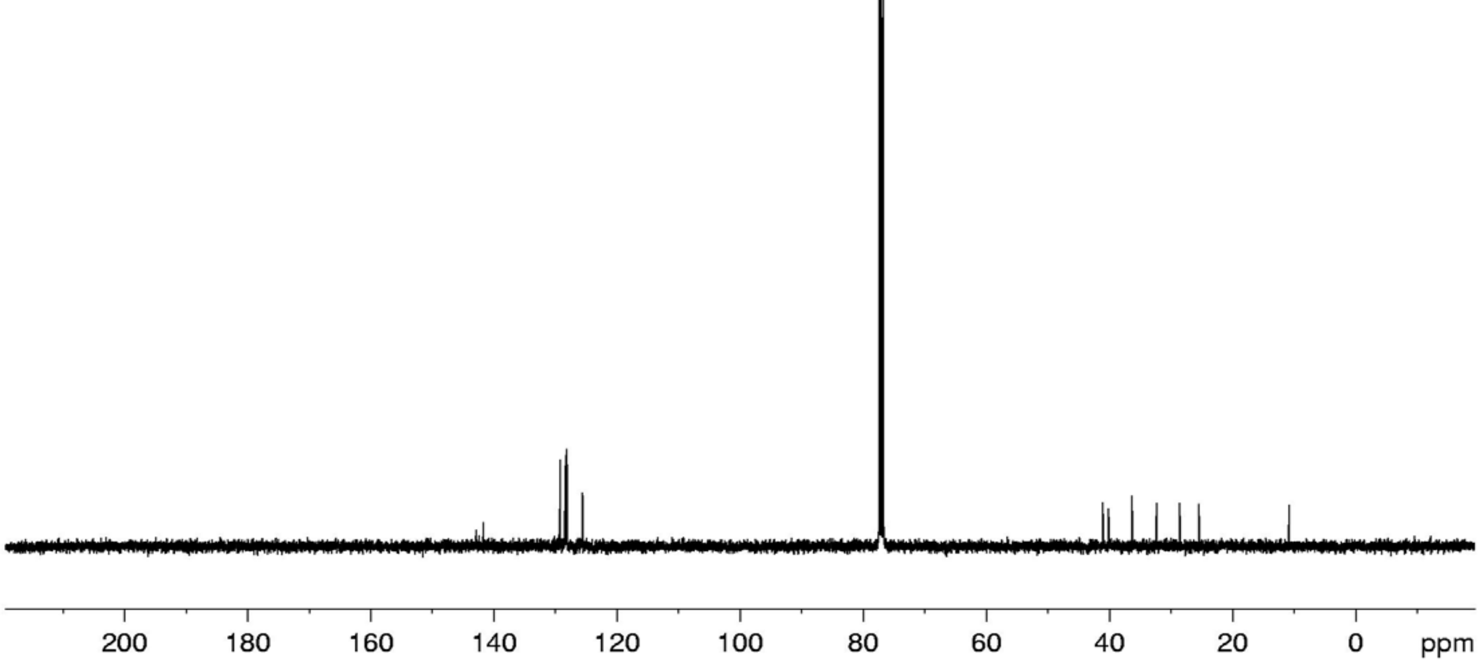

200
180
160
140
120
100
80
60
40
20
0
ppm

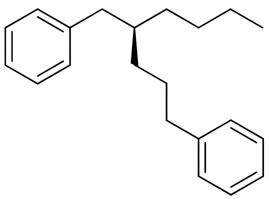

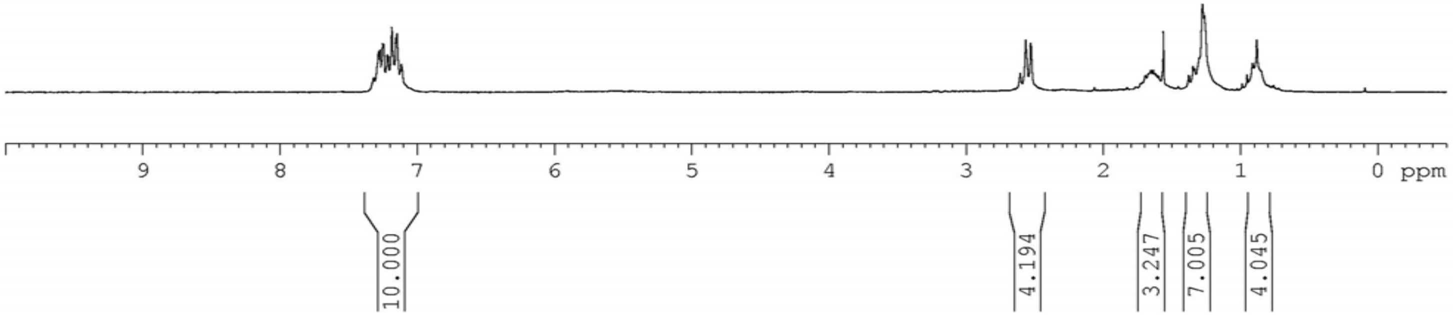

9
8
7
6
5
4
3
2
1
0 ppm
10.000
4.194
3.247
7.005
4.045

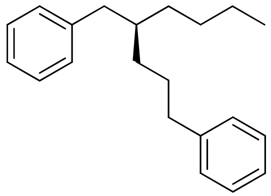

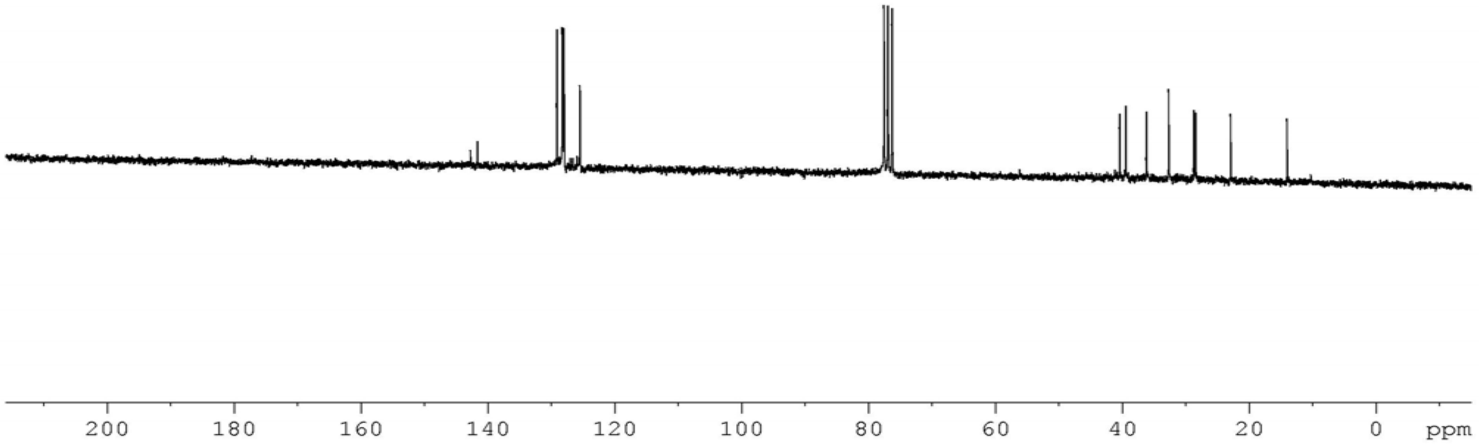
200
180
160
140
120
100
80
60
40
20
0
ppm

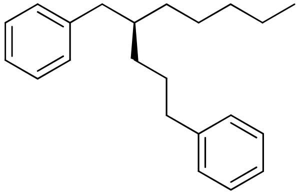

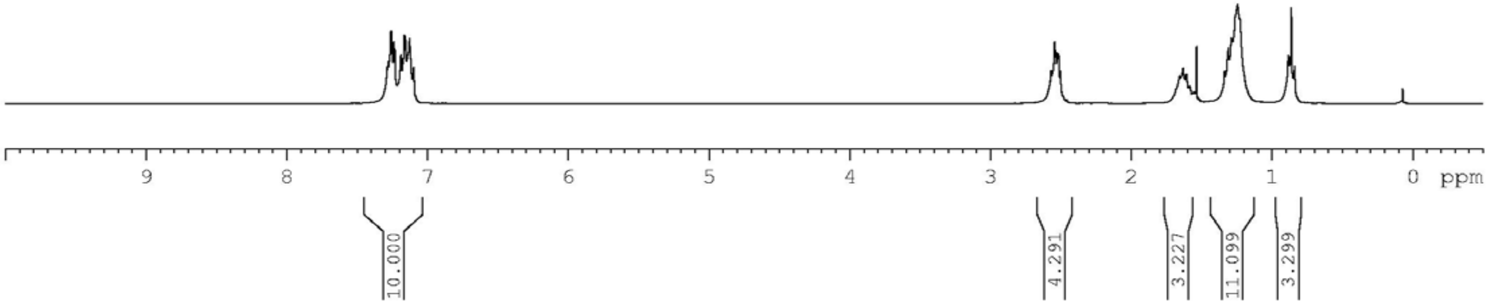
9
8
7
6
5
4
3
2
1
0 ppm
10.000
4.291
3.227
11.099
3.299

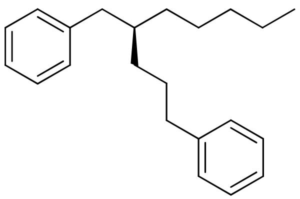

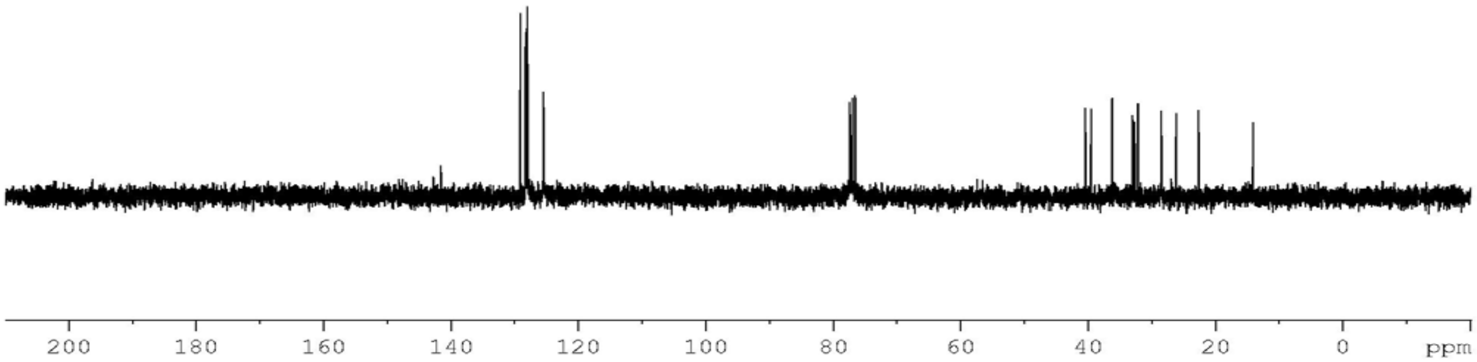
200 180 160 140 120 100 80 60 40 20 0 ppm

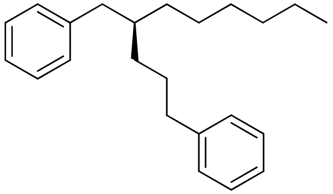

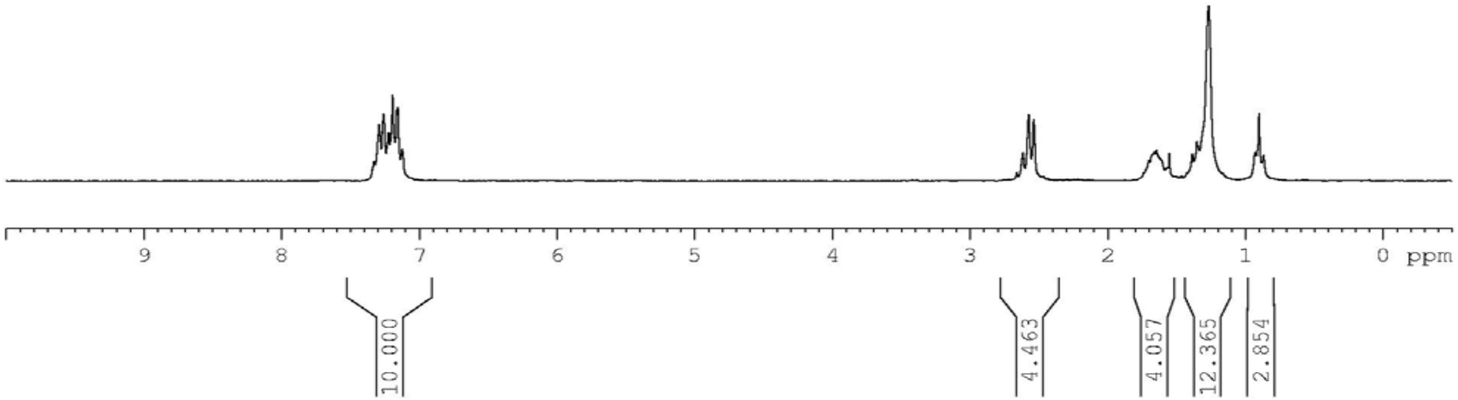
9 8 7 6 5 4 3 2 1 0 ppm
10.000
4.463
4.057
12.365
2.854

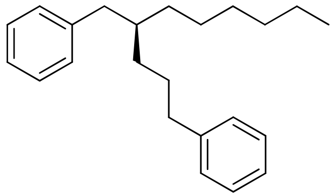

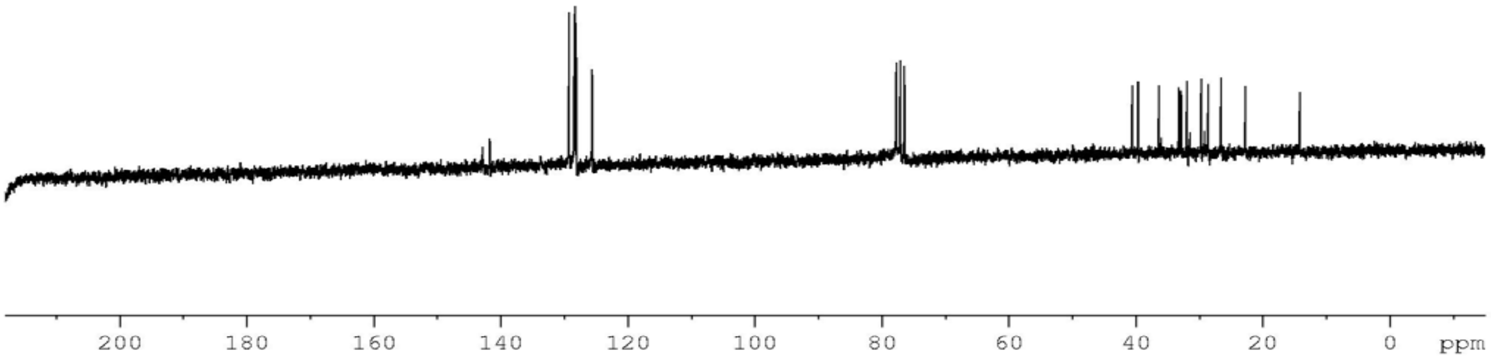
200
180
160
140
120
100
80
60
40
20
0
ppm

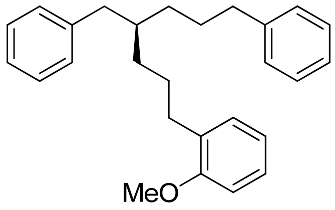
MeO

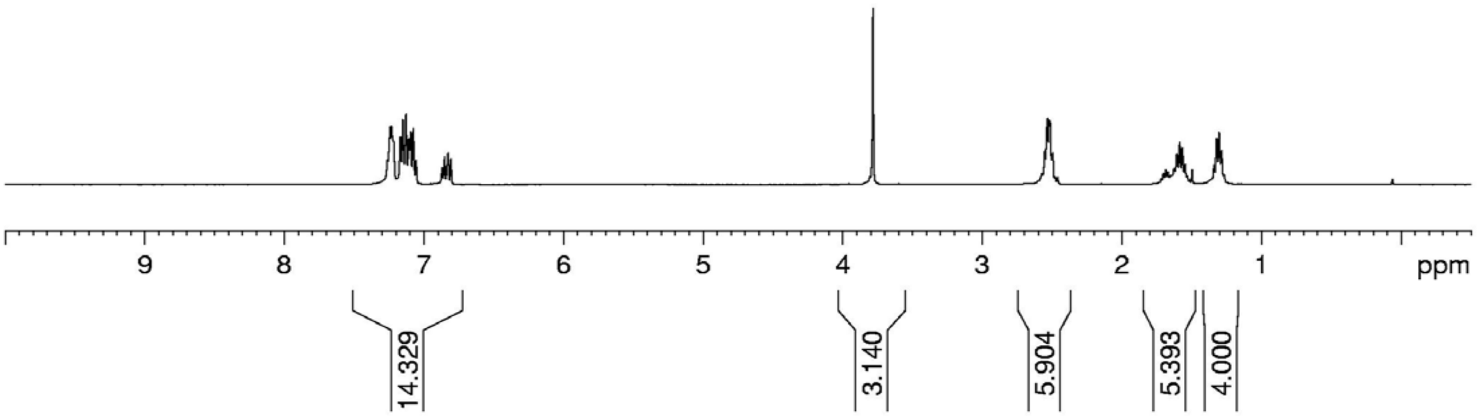
9
8
7
6
5
4
3
2
1
ppm
14.329
3.140
5.904
5.393
4.000

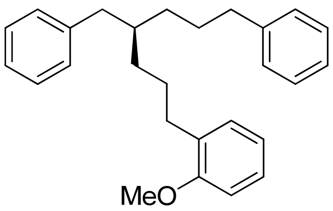
MeO

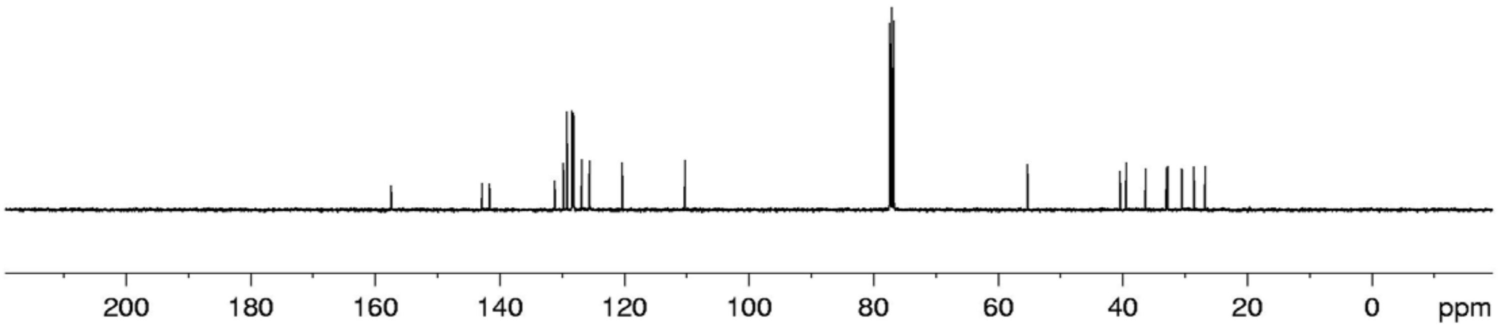
200
180
160
140
120
100
80
60
40
20
0
ppm

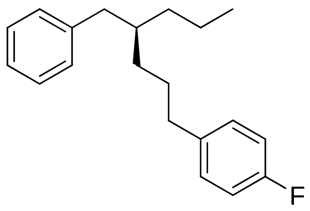
F

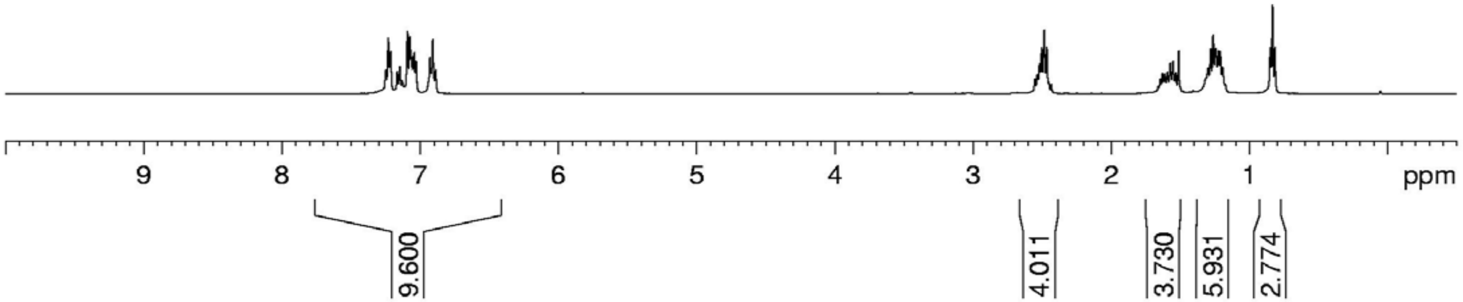
9
8
7
6
5
4
3
2
1
ppm
9.600
4.011
3.730
5.931
2.774

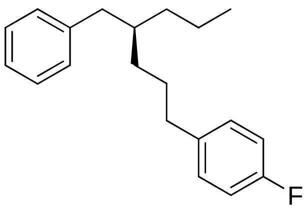
F

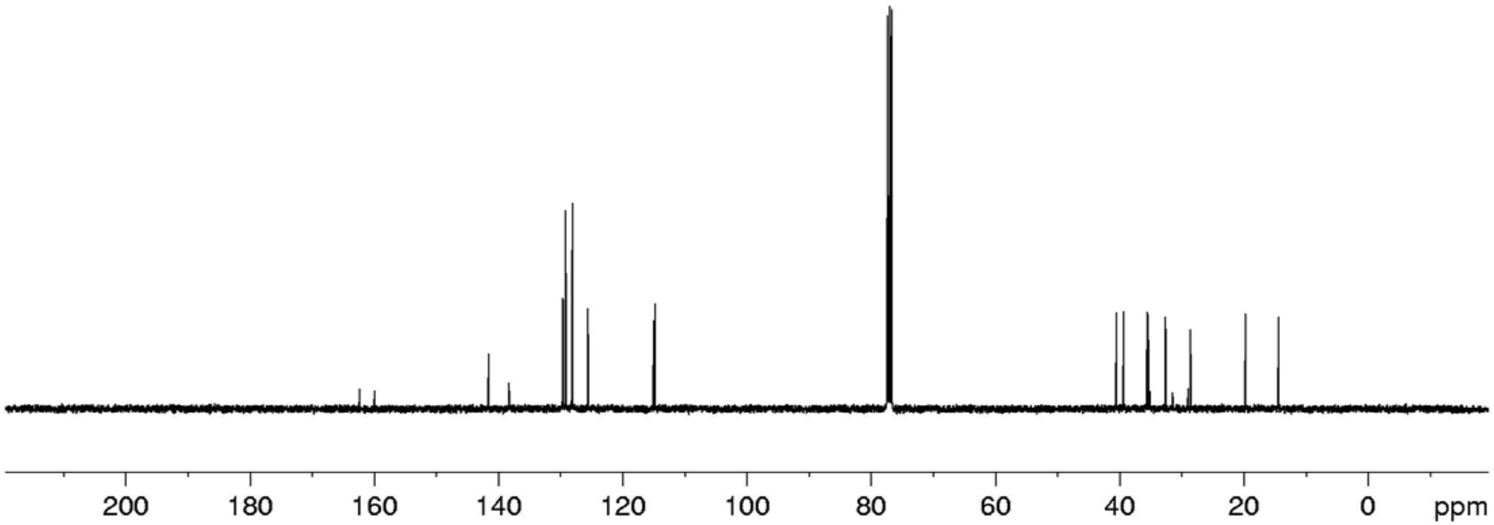
200
180
160
140
120
100
80
60
40
20
0
ppm

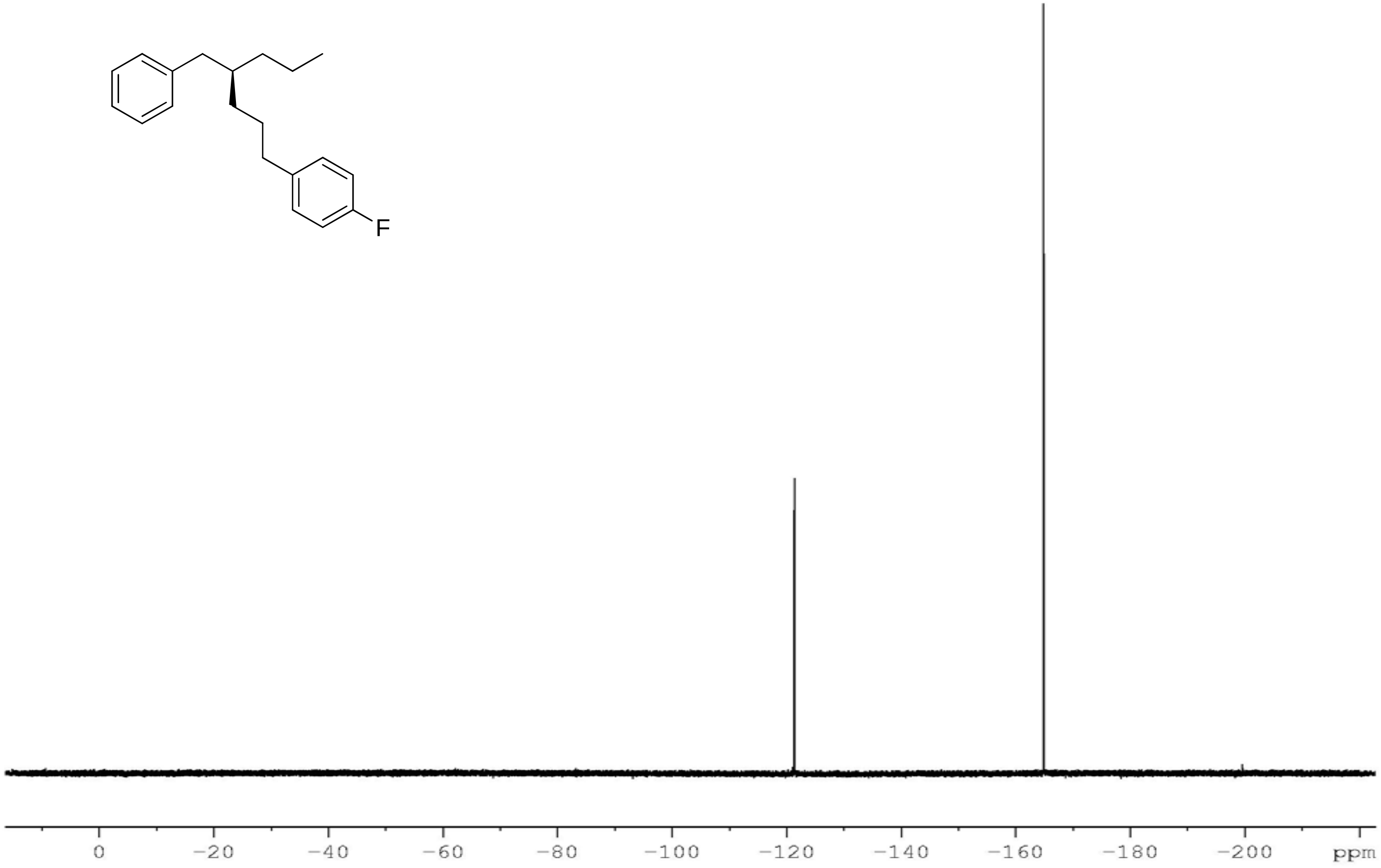
F
0
−20
−40
−60
−80
−100
−120
−140
−160
−180
−200
ppm

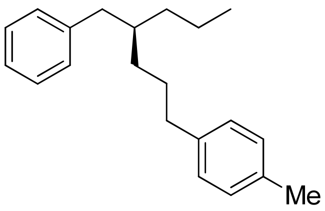
Me

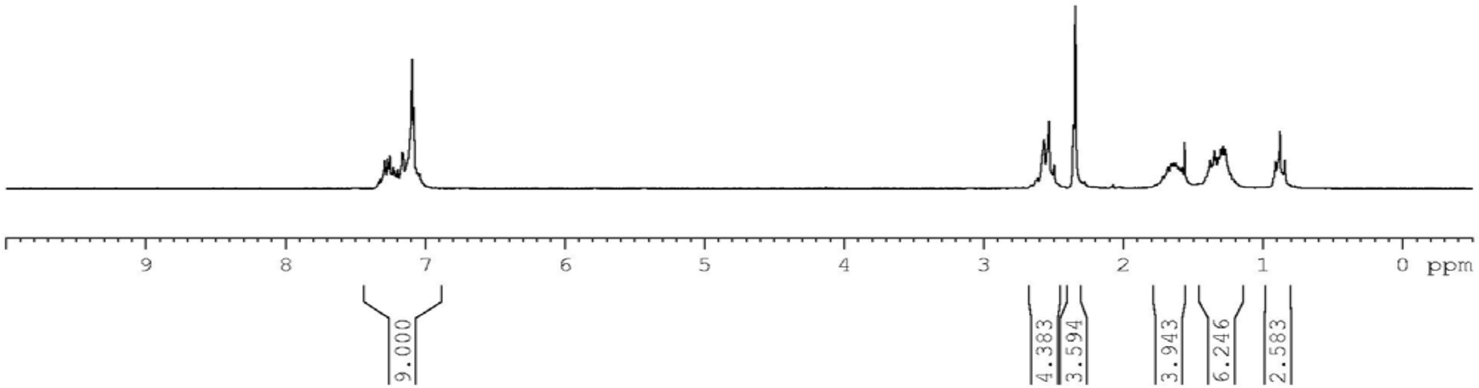
9
8
7
6
5
4
3
2
1
0 ppm
9.000
4.383
3.594
3.943
6.246
2.583

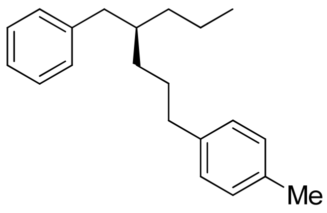
Me

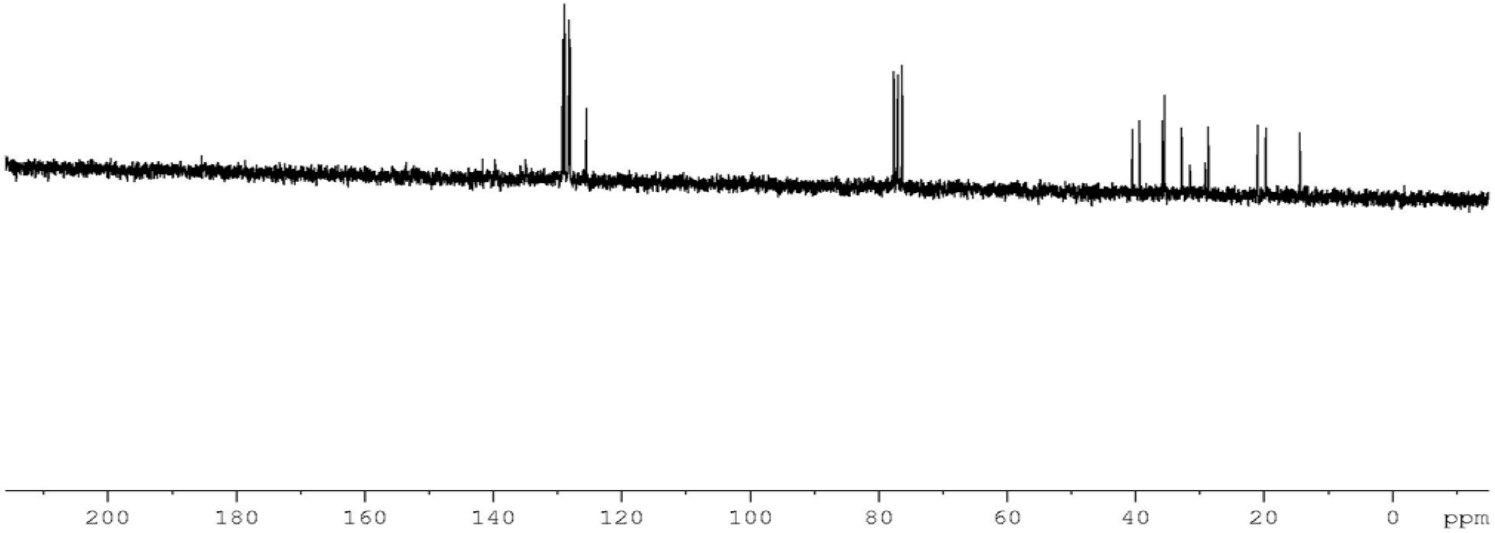
200
180
160
140
120
100
80
60
40
20
0
ppm

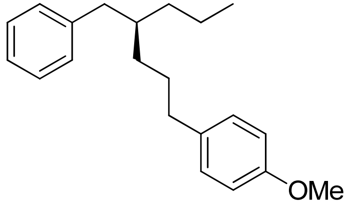
OMe

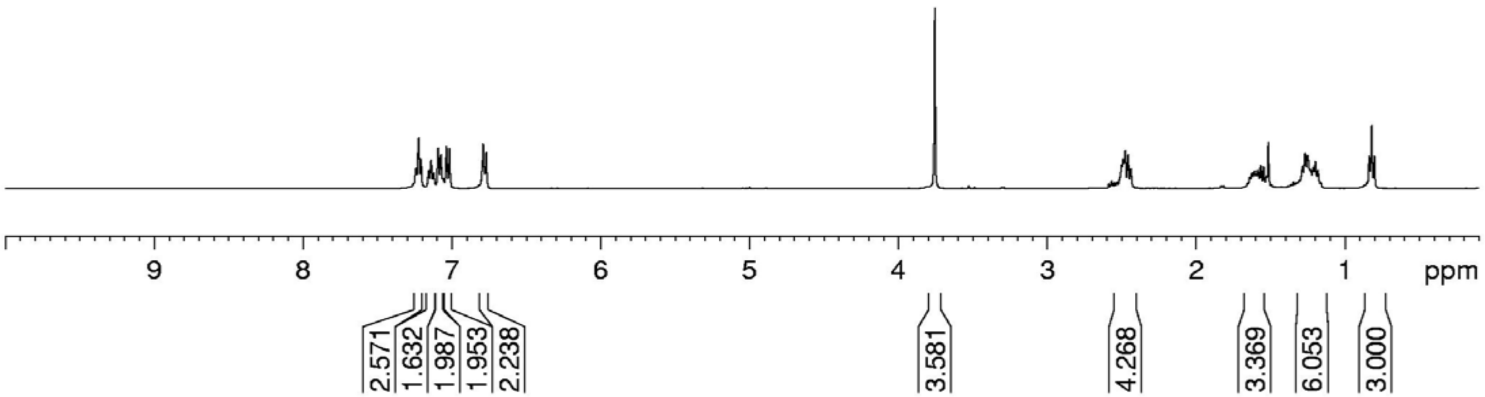
9
8
7
6
5
4
3
2
1
ppm
2.571
1.632
1.987
1.953
2.238
3.581
4.268
3.369
6.053
3.000

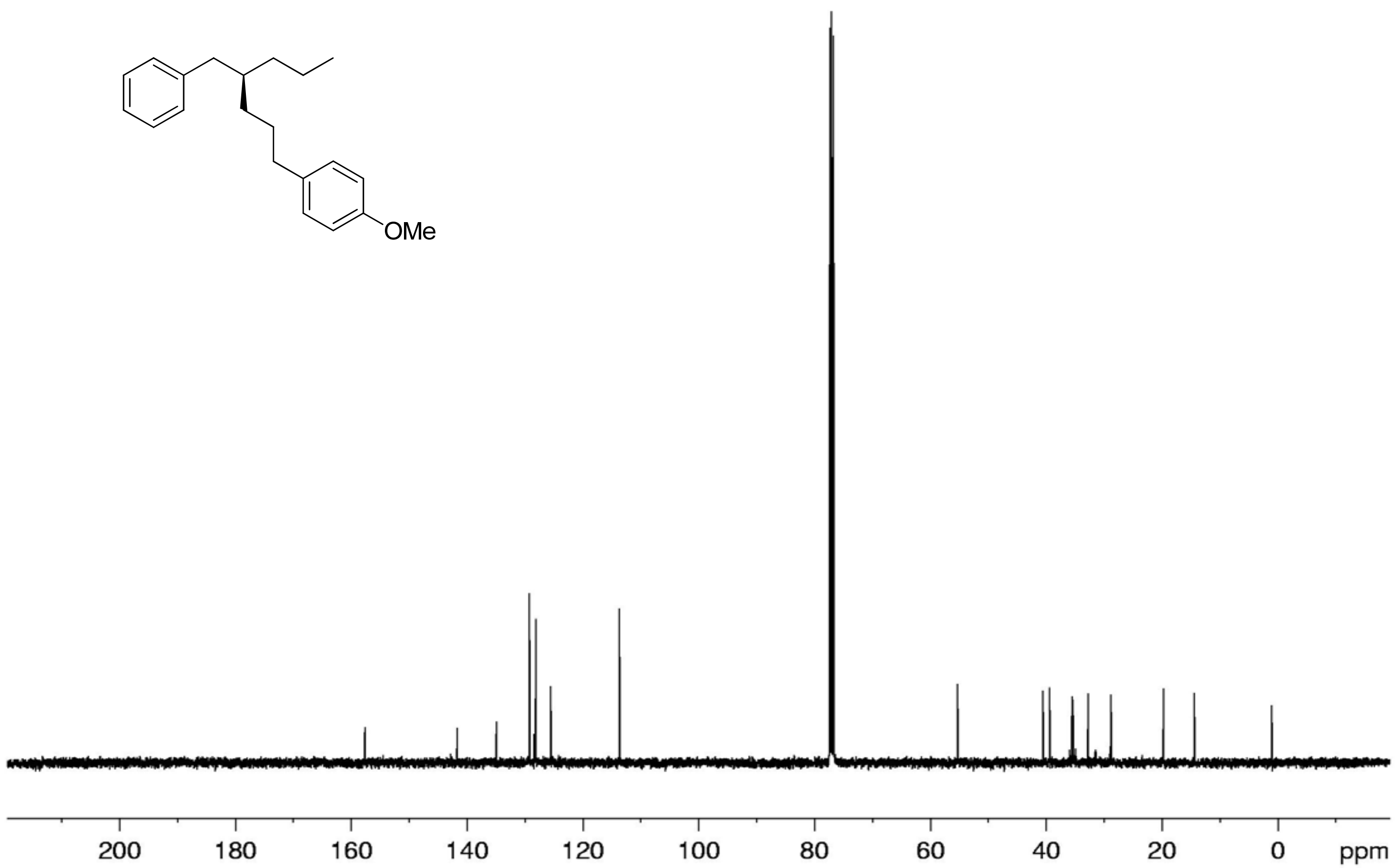
OMe
200
180
160
140
120
100
80
60
40
20
0
ppm

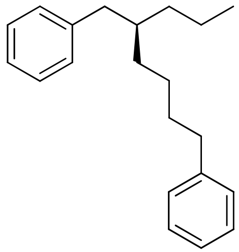

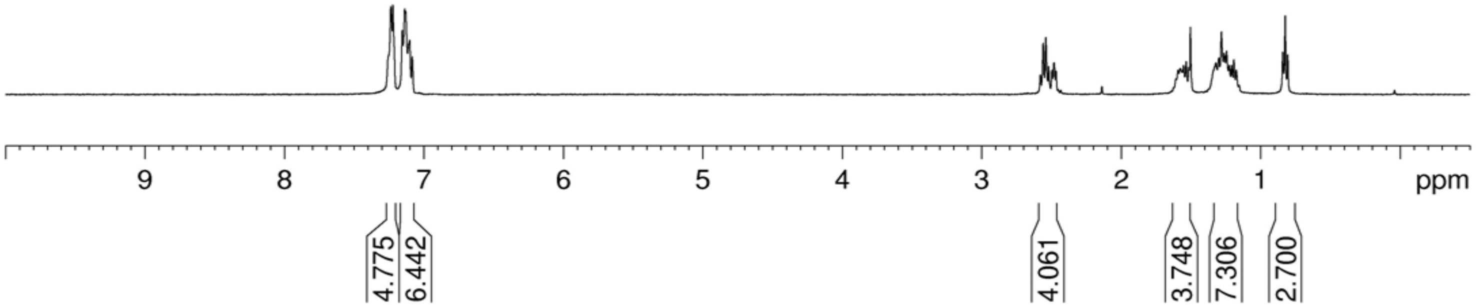

9
8
7
6
5
4
3
2
1
ppm
4.775
6.442
4.061
3.748
7.306
2.700

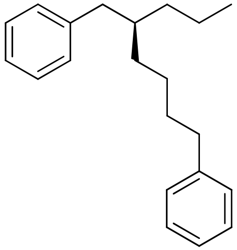

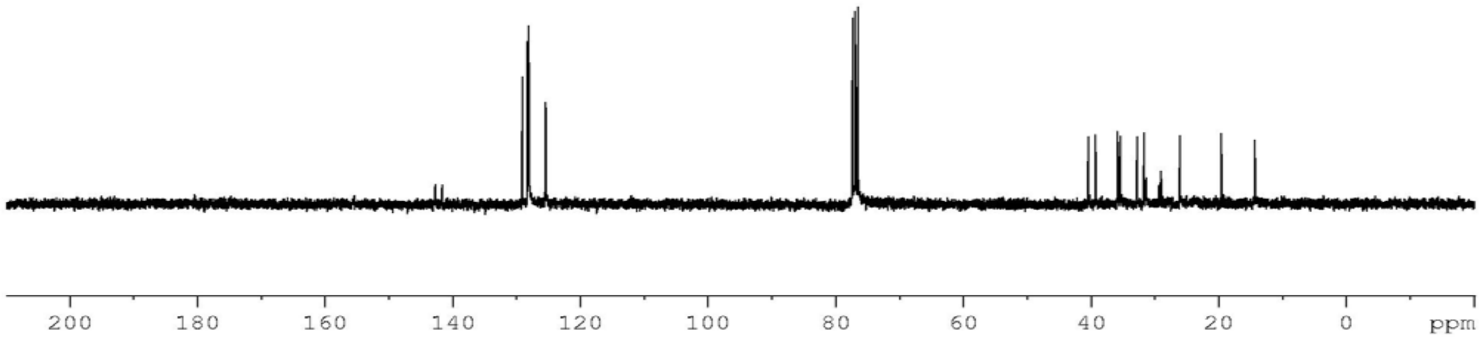
200
180
160
140
120
100
80
60
40
20
0
ppm

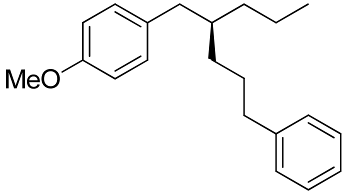
MeO

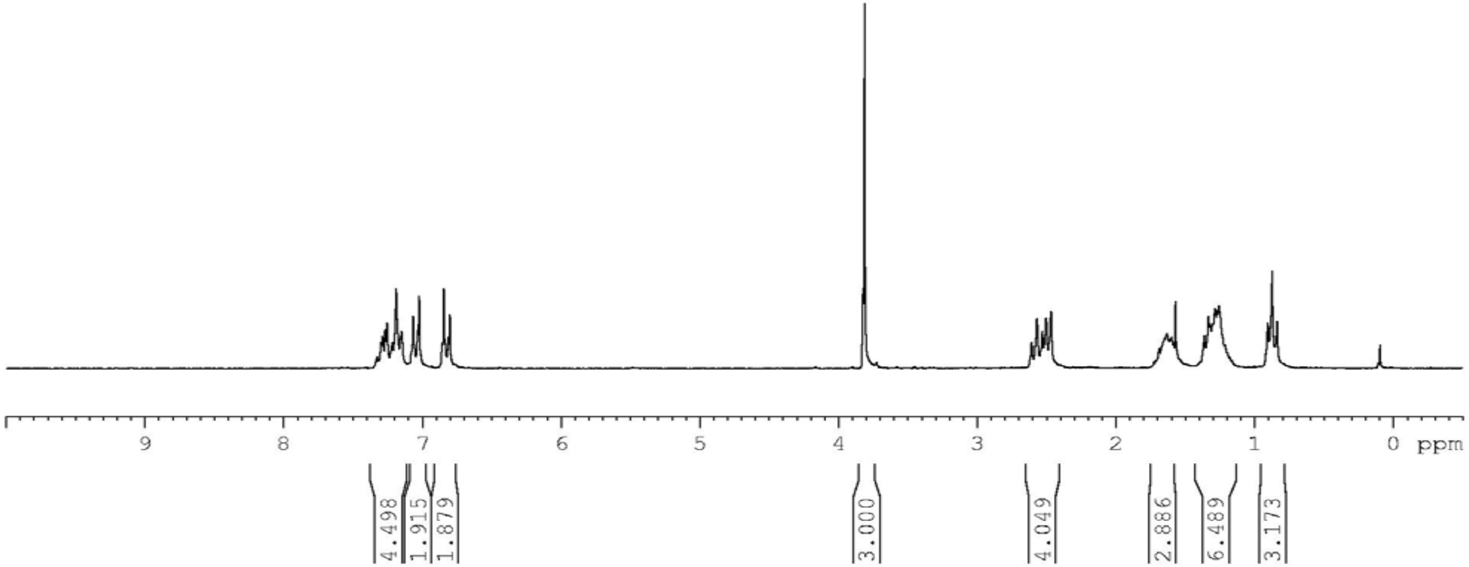
9
8
7
6
5
4
3
2
1
0 ppm
4.498
1.915
1.879
3.000
4.049
2.886
6.489
3.173

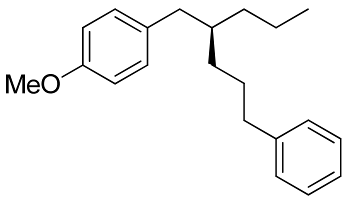
MeO

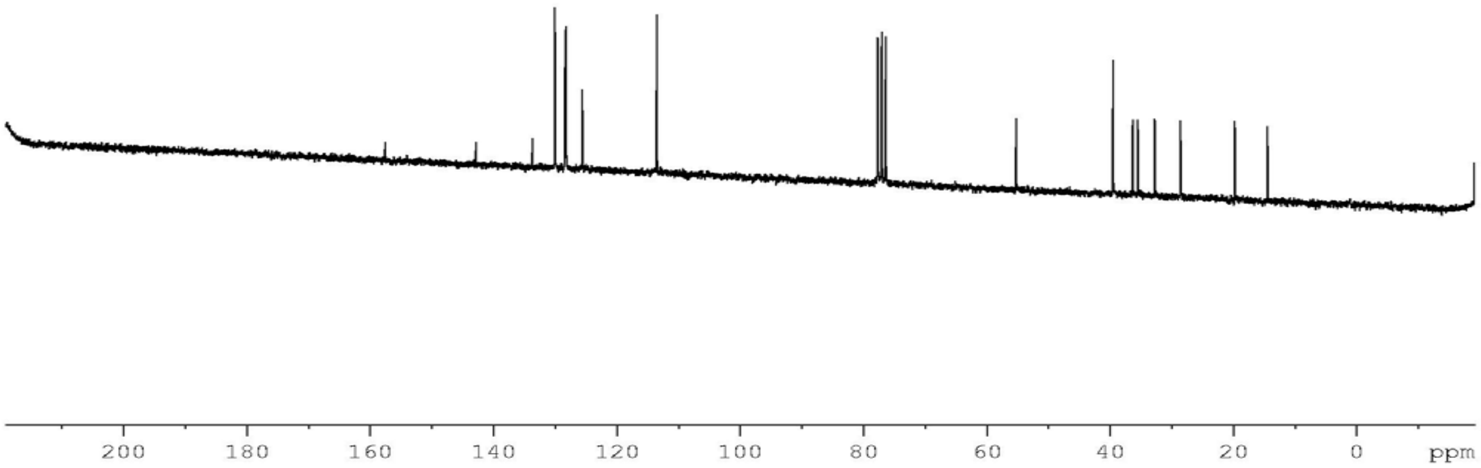
200
180
160
140
120
100
80
60
40
20
0
ppm

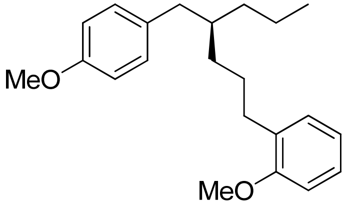
MeO
MeO

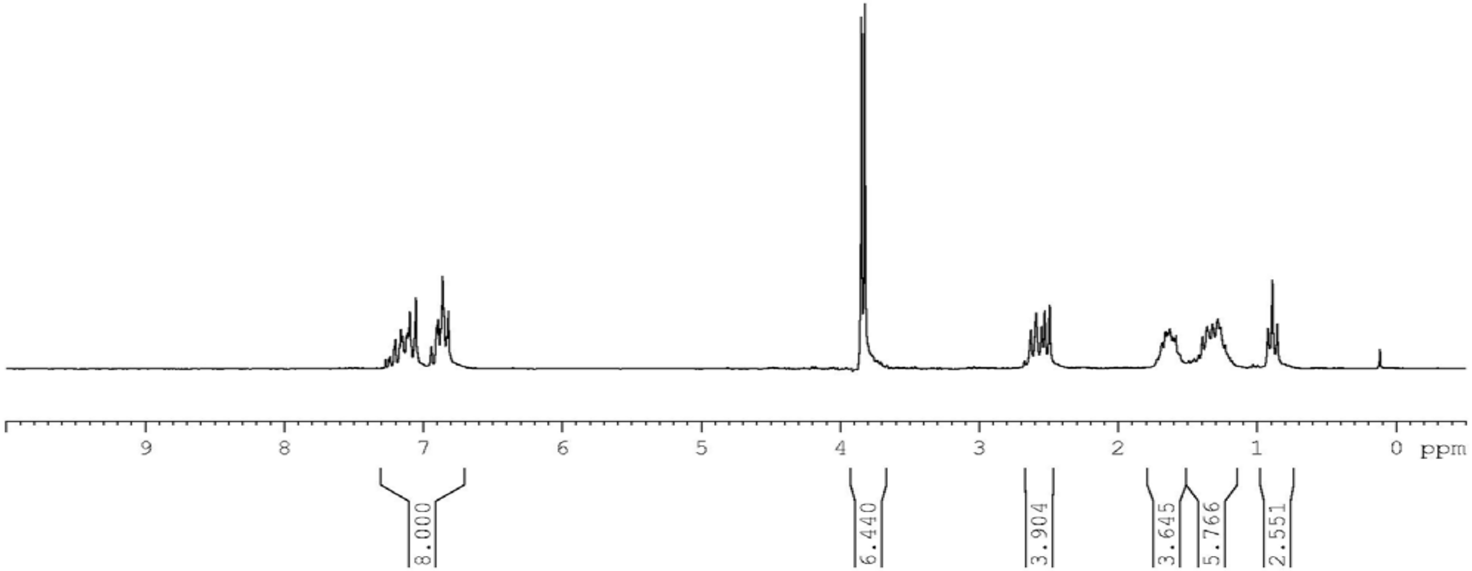
9
8
7
6
5
4
3
2
1
0 ppm
8.000
6.440
3.904
3.645
5.766
2.551

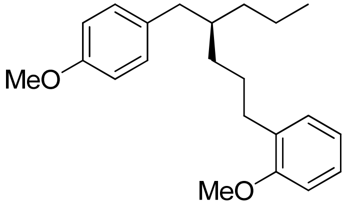
MeO
MeO

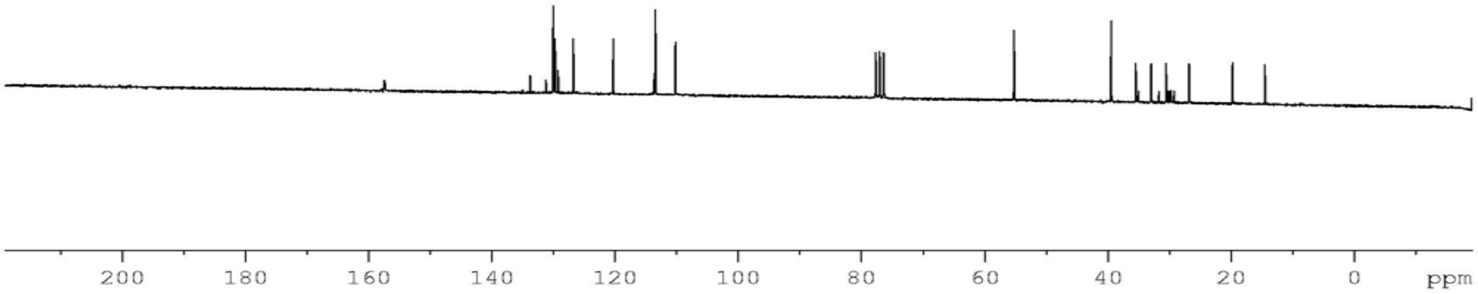
200
180
160
140
120
100
80
60
40
20
0
ppm

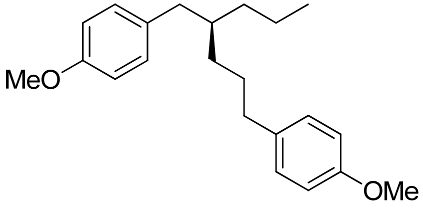
MeO
OMe

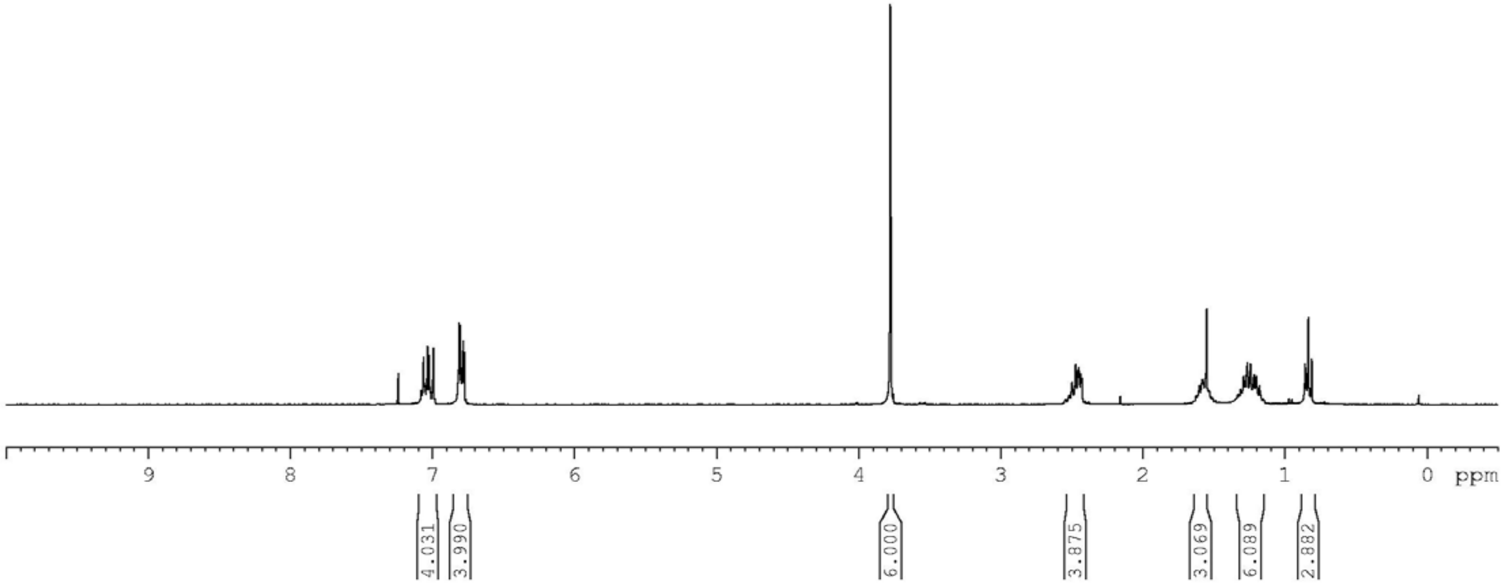
9
8
7
6
5
4
3
2
1
0
ppm
4.031
3.990
6.000
3.875
3.069
6.089
2.882

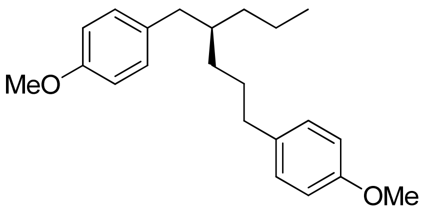
MeO
OMe

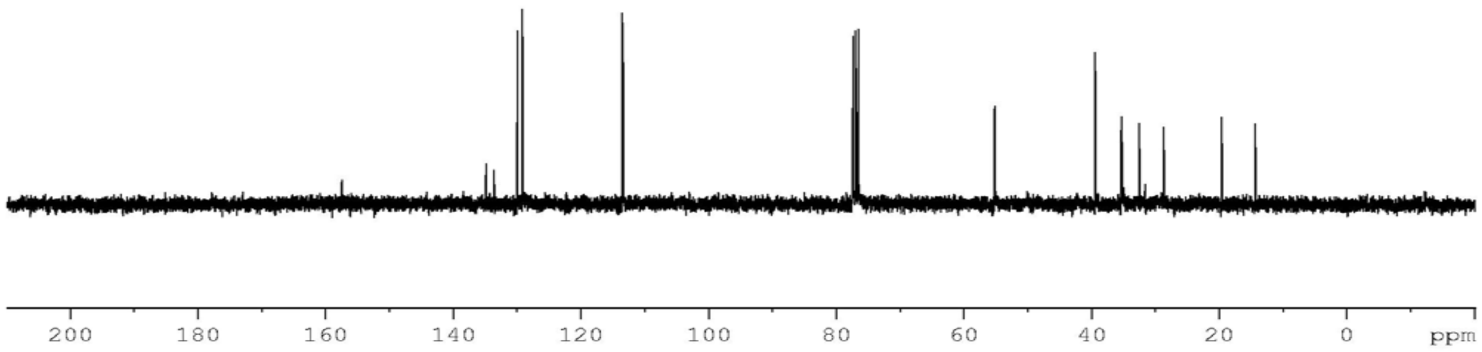
200
180
160
140
120
100
80
60
40
20
0
ppm

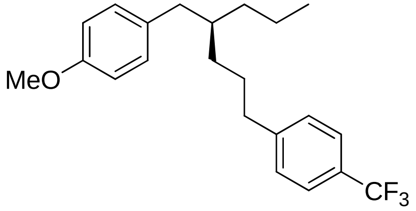
MeO
CF3

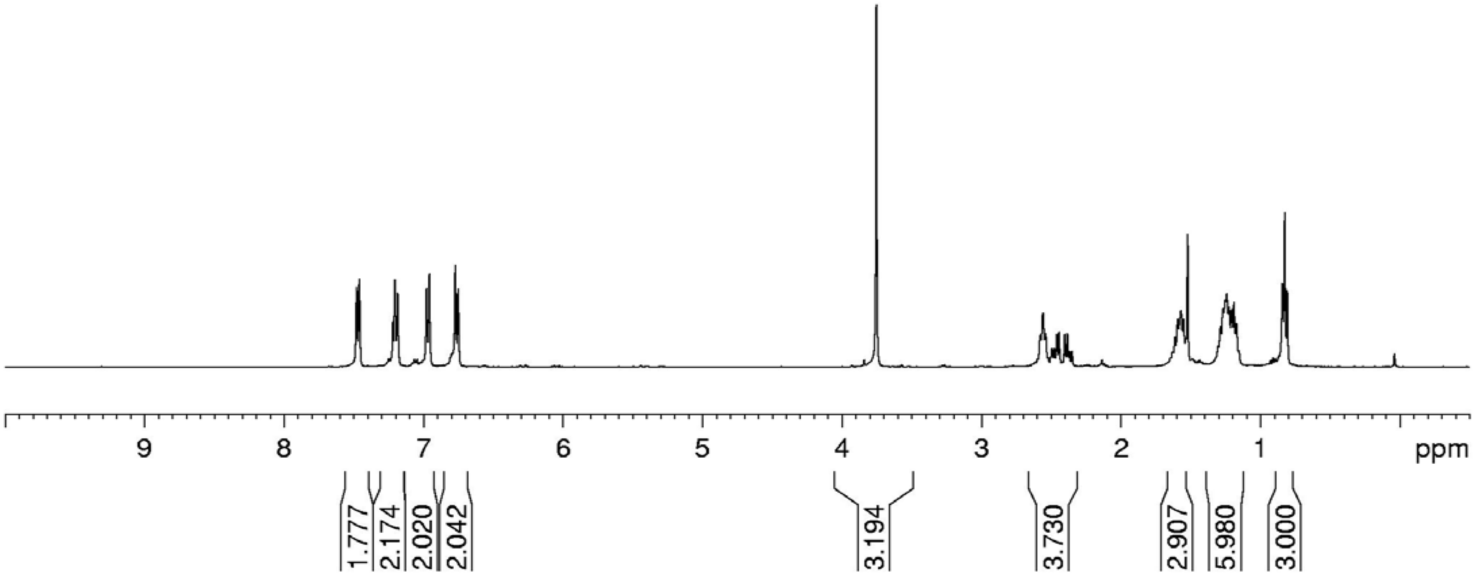
9
8
7
6
5
4
3
2
1
ppm
1.777
2.174
2.020
2.042
3.194
3.730
2.907
5.980
3.000

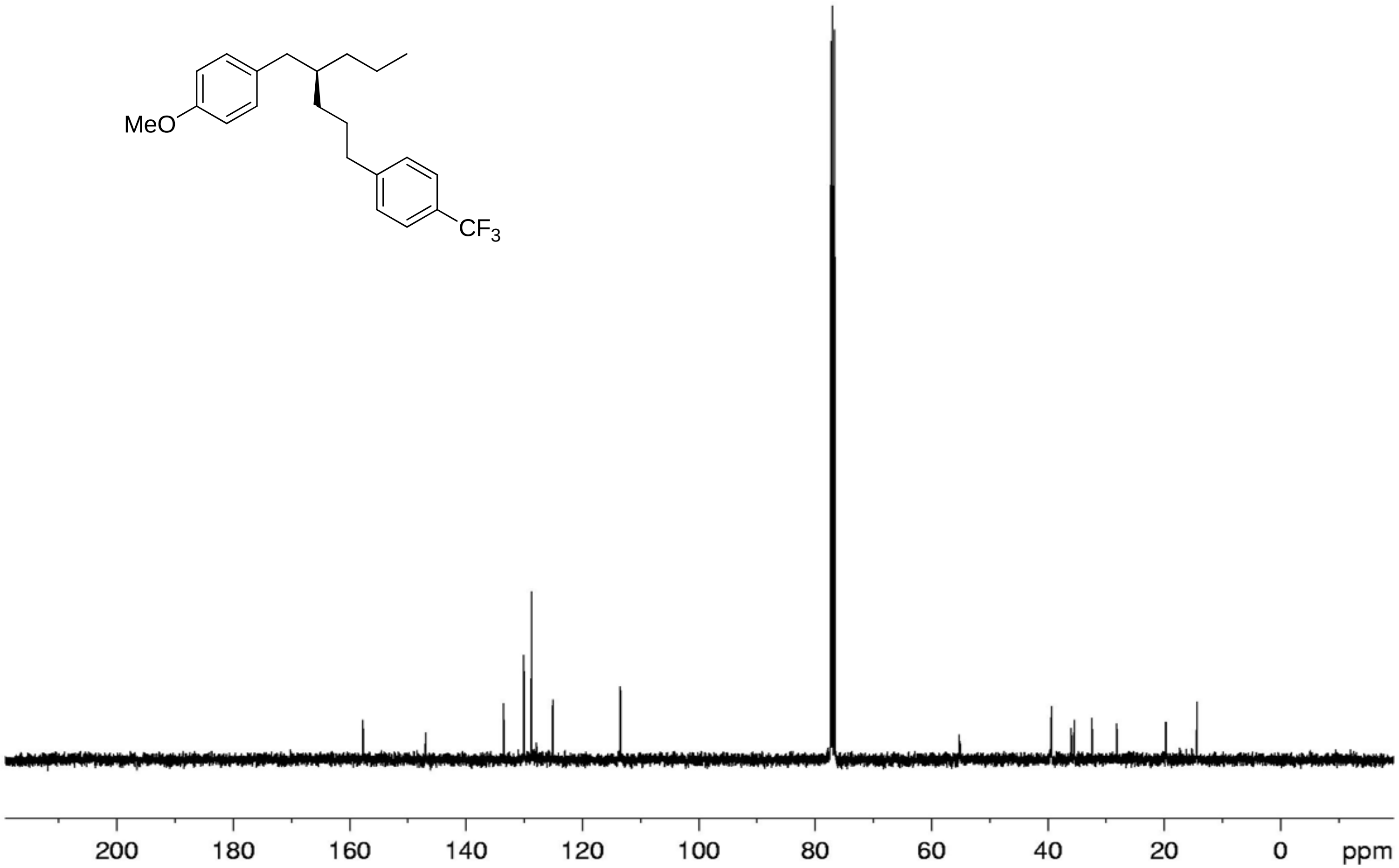
MeO
CF3
200
180
160
140
120
100
80
60
40
20
0
ppm

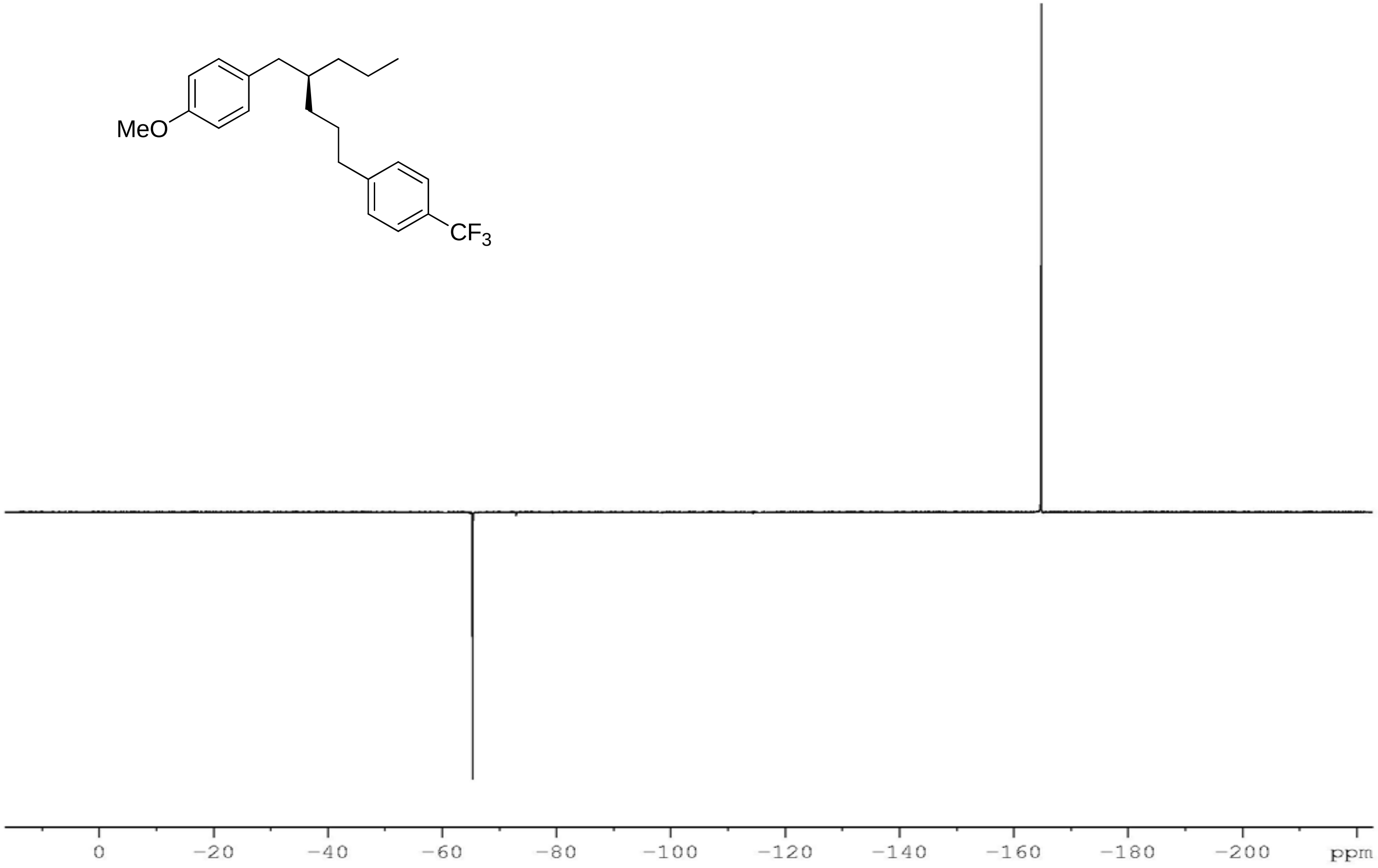
MeO
CF3
0
−20
−40
−60
−80
−100
−120
−140
−160
−180
−200
ppm

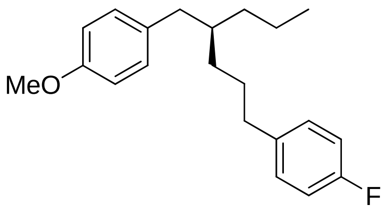
MeO
F

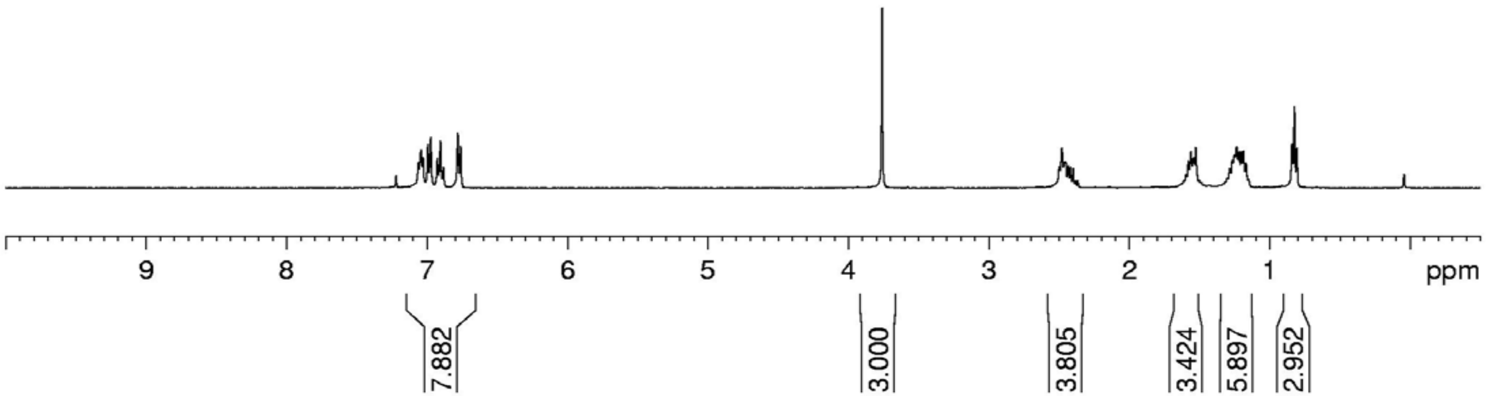
9
8
7
6
5
4
3
2
1
ppm
7.882
3.000
3.805
3.424
5.897
2.952

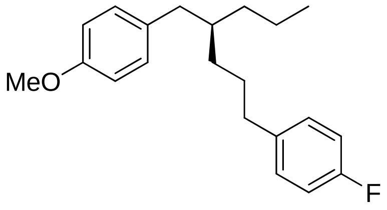
MeO
F

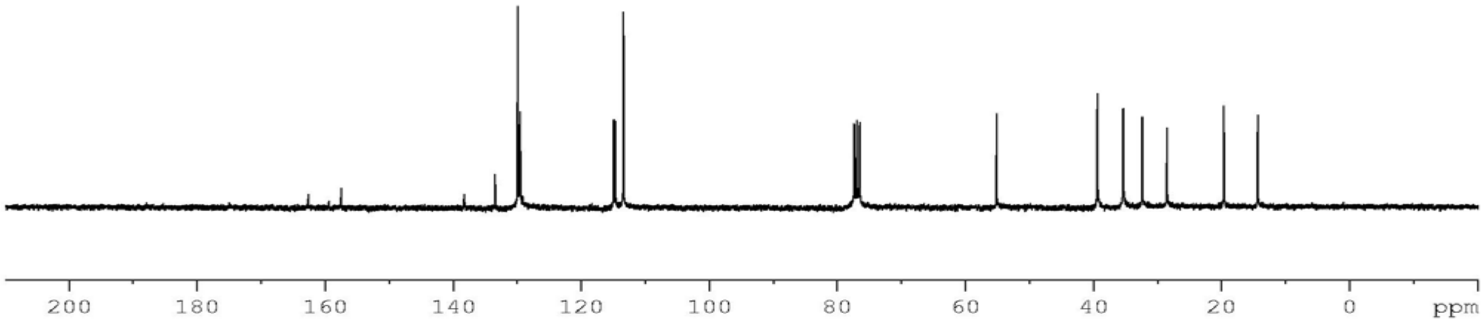
200 180 160 140 120 100 80 60 40 20 0 ppm

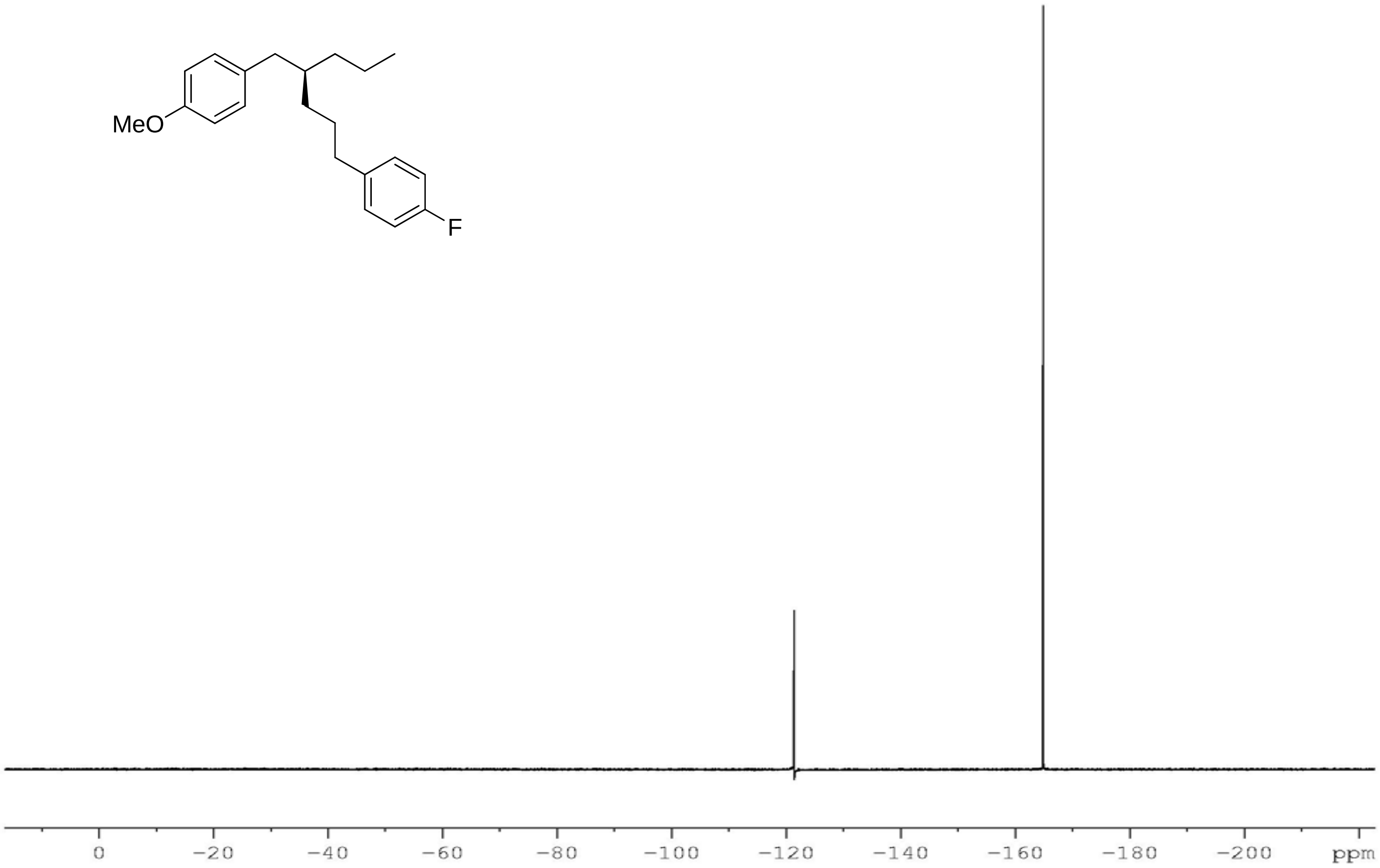
MeO
F
0
−20
−40
−60
−80
−100
−120
−140
−160
−180
−200
ppm

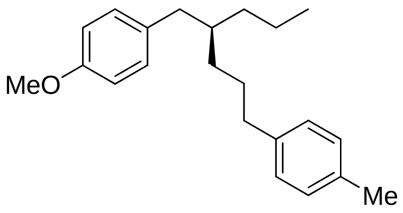
MeO
Me

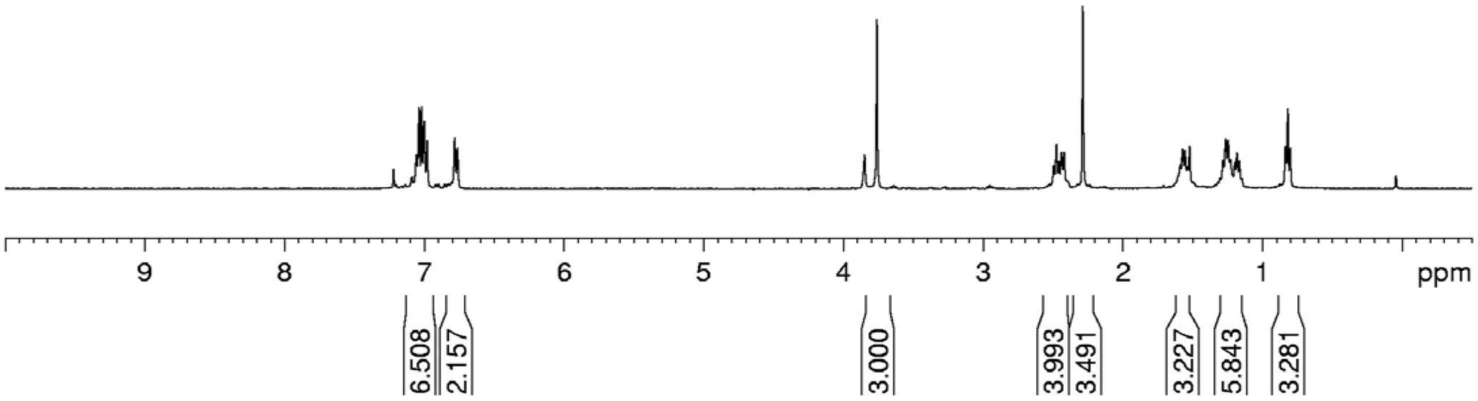
9
8
7
6
5
4
3
2
1
ppm
6.508
2.157
3.000
3.993
3.491
3.227
5.843
3.281

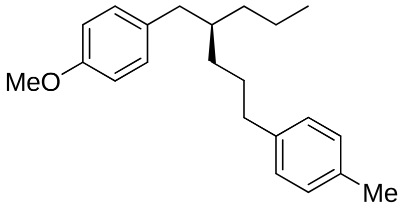
MeO
Me

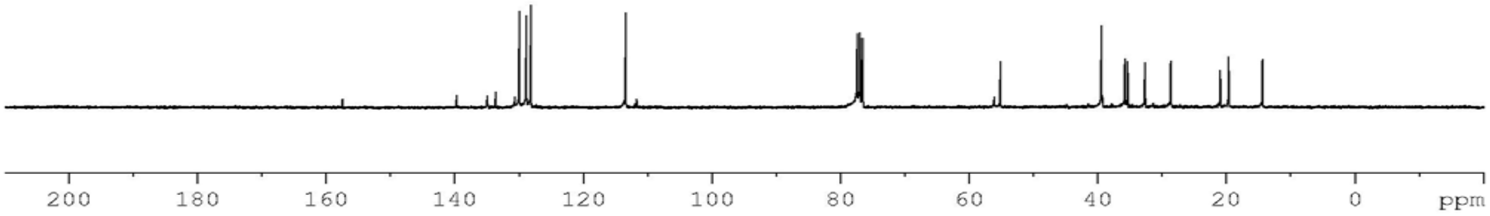
200
180
160
140
120
100
80
60
40
20
0
ppm

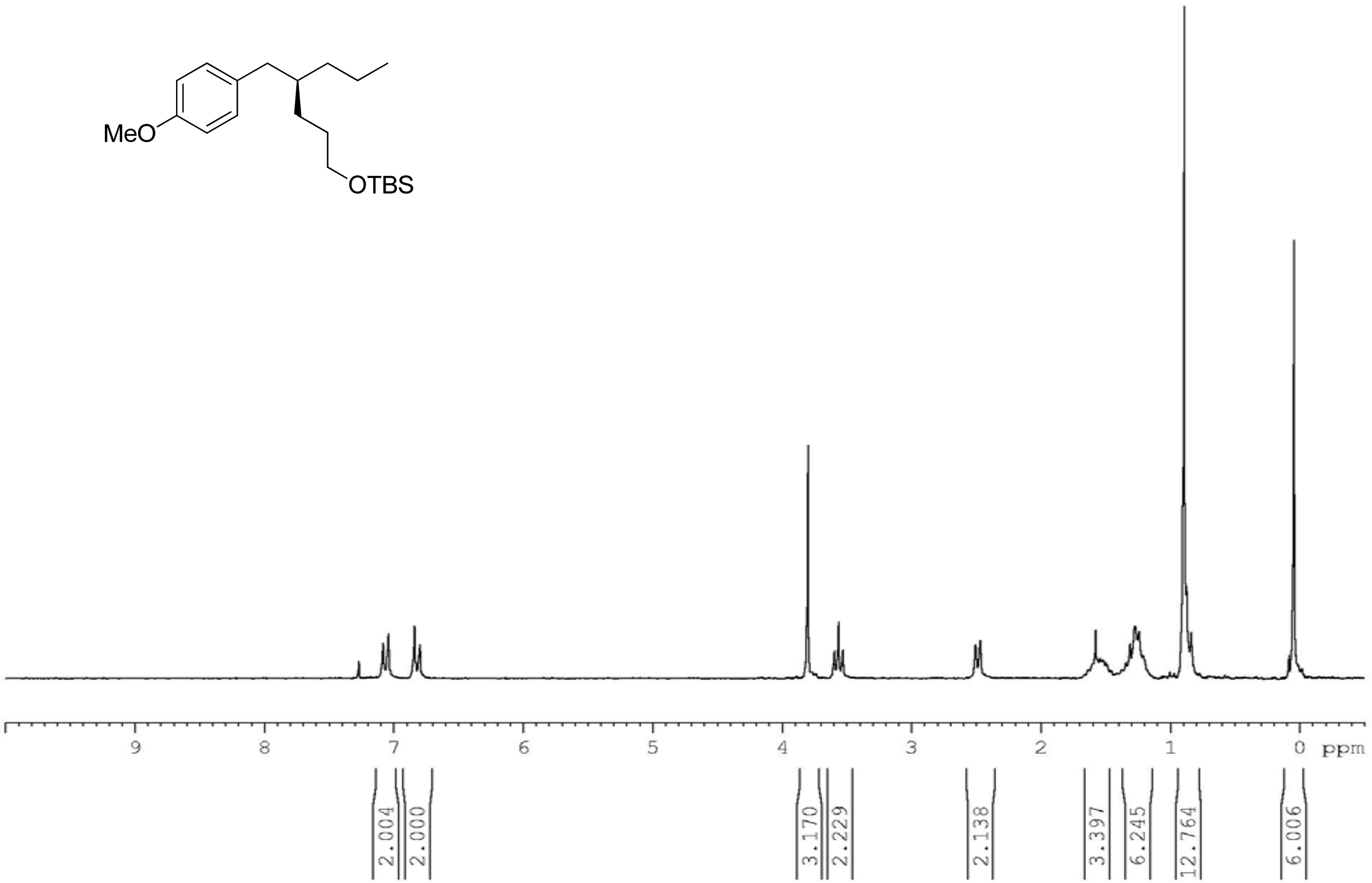
MeO
OTBS
9
8
7
6
5
4
3
2
1
0 ppm
2.004
2.000
3.170
2.229
2.138
3.397
6.245
12.764
6.006

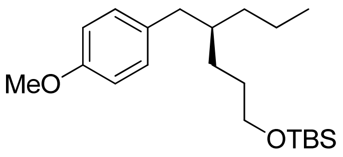

MeO
OTBS

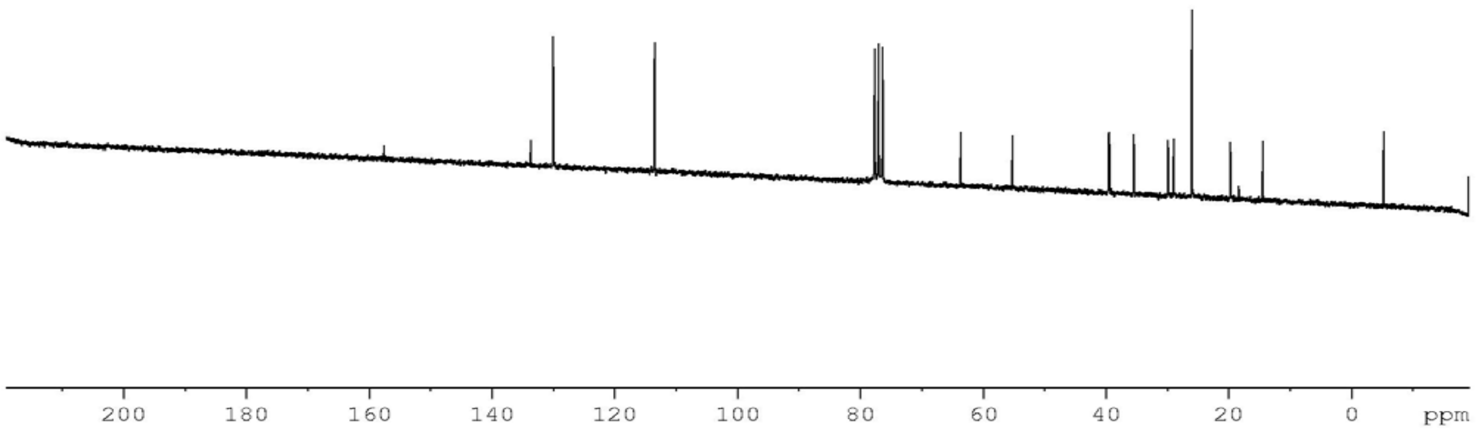

200
180
160
140
120
100
80
60
40
20
0
ppm

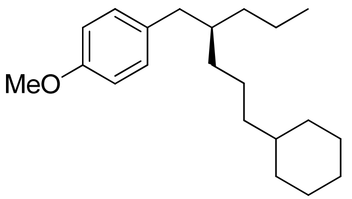
MeO

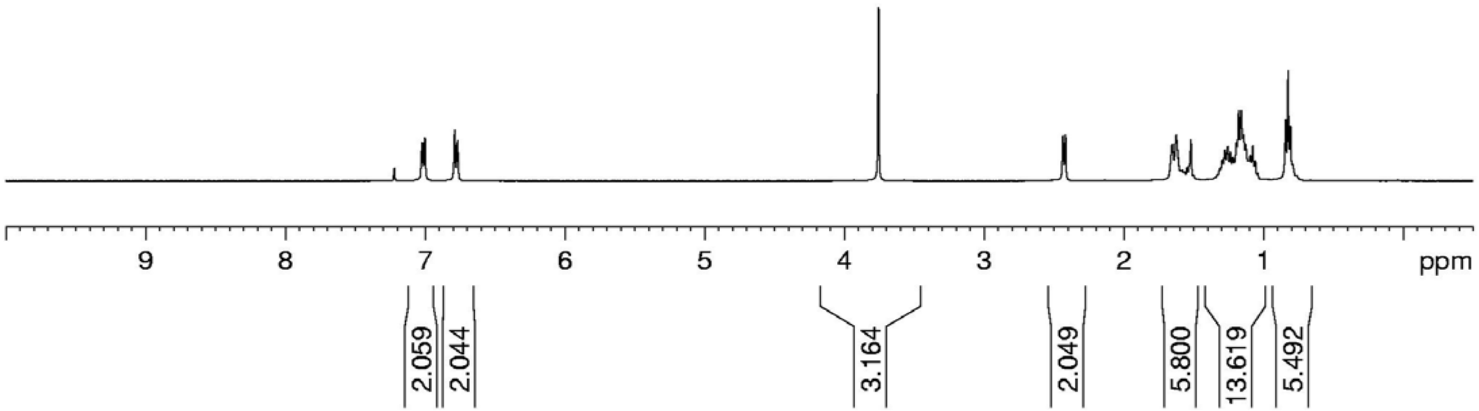
9
8
7
6
5
4
3
2
1
ppm
2.059
2.044
3.164
2.049
5.800
13.619
5.492

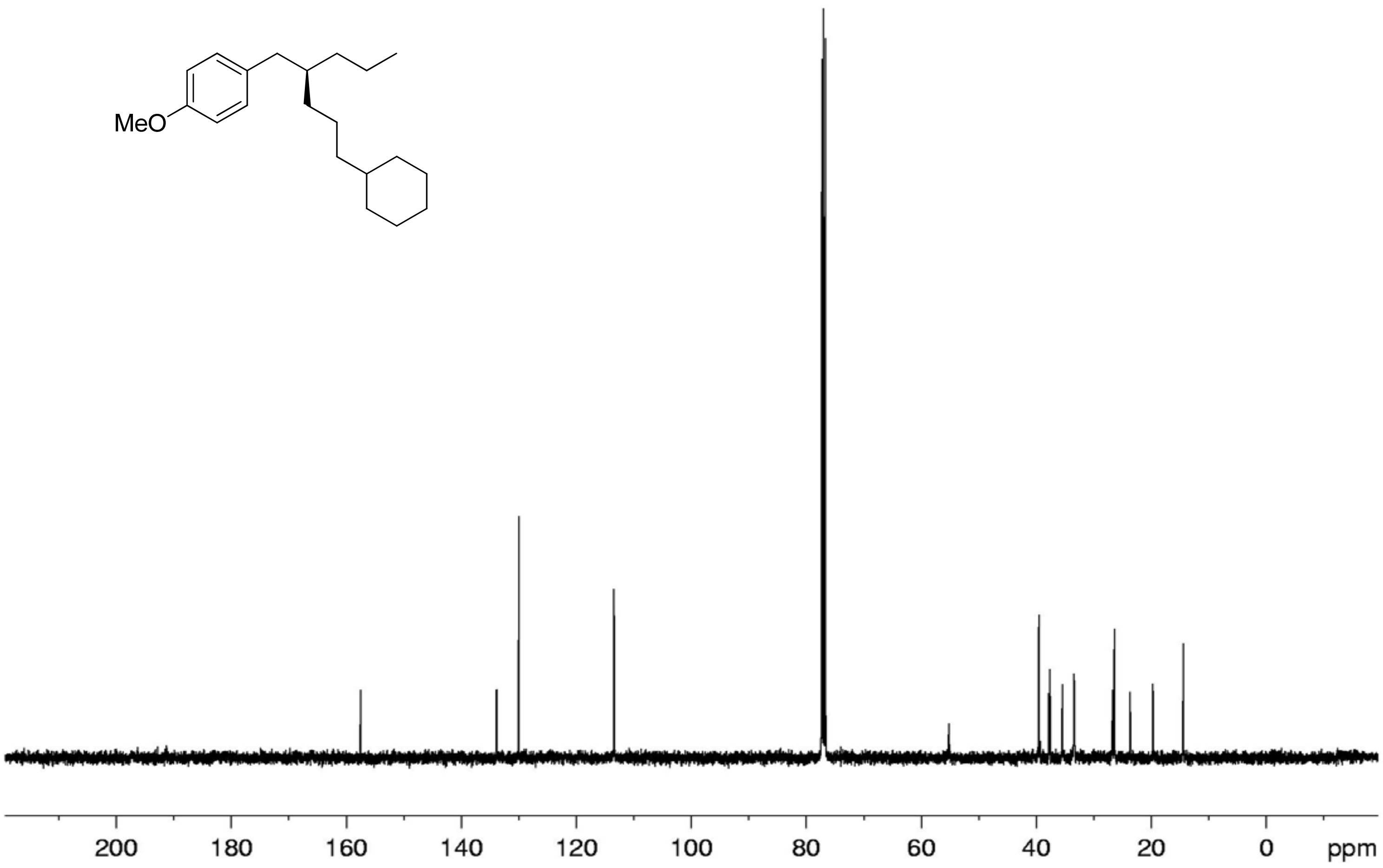
MeO
200
180
160
140
120
100
80
60
40
20
0
ppm

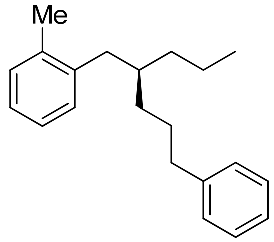
Me

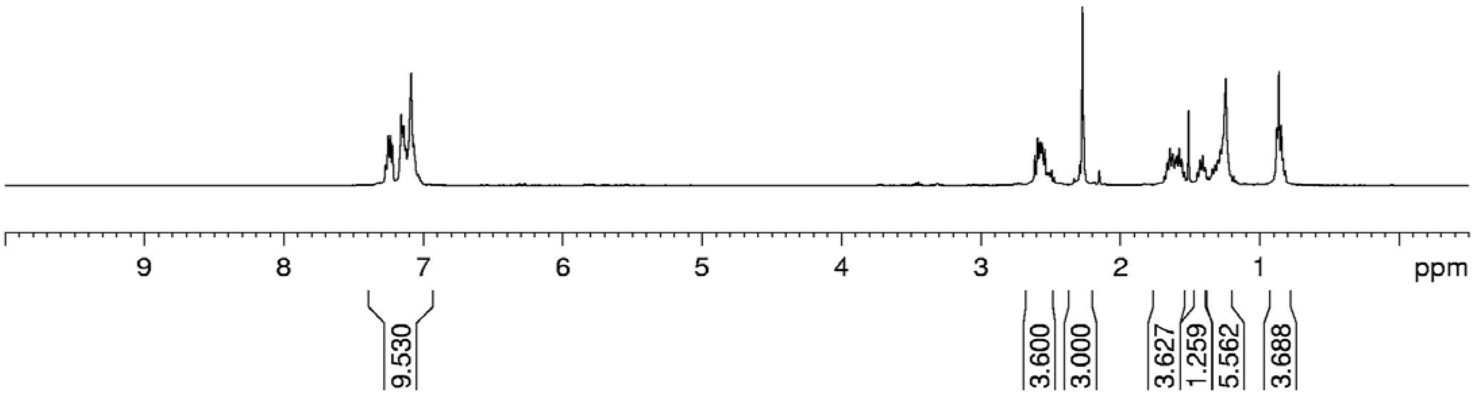
9
8
7
6
5
4
3
2
1
ppm
9.530
3.600
3.000
3.627
1.259
5.562
3.688

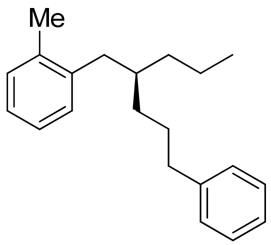
Me

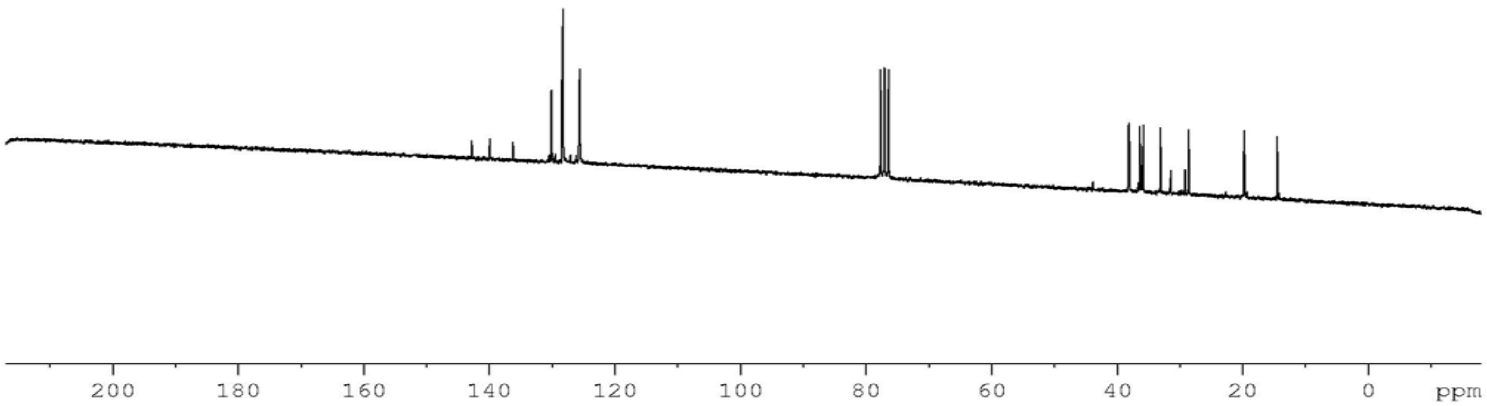
200
180
160
140
120
100
80
60
40
20
0
ppm

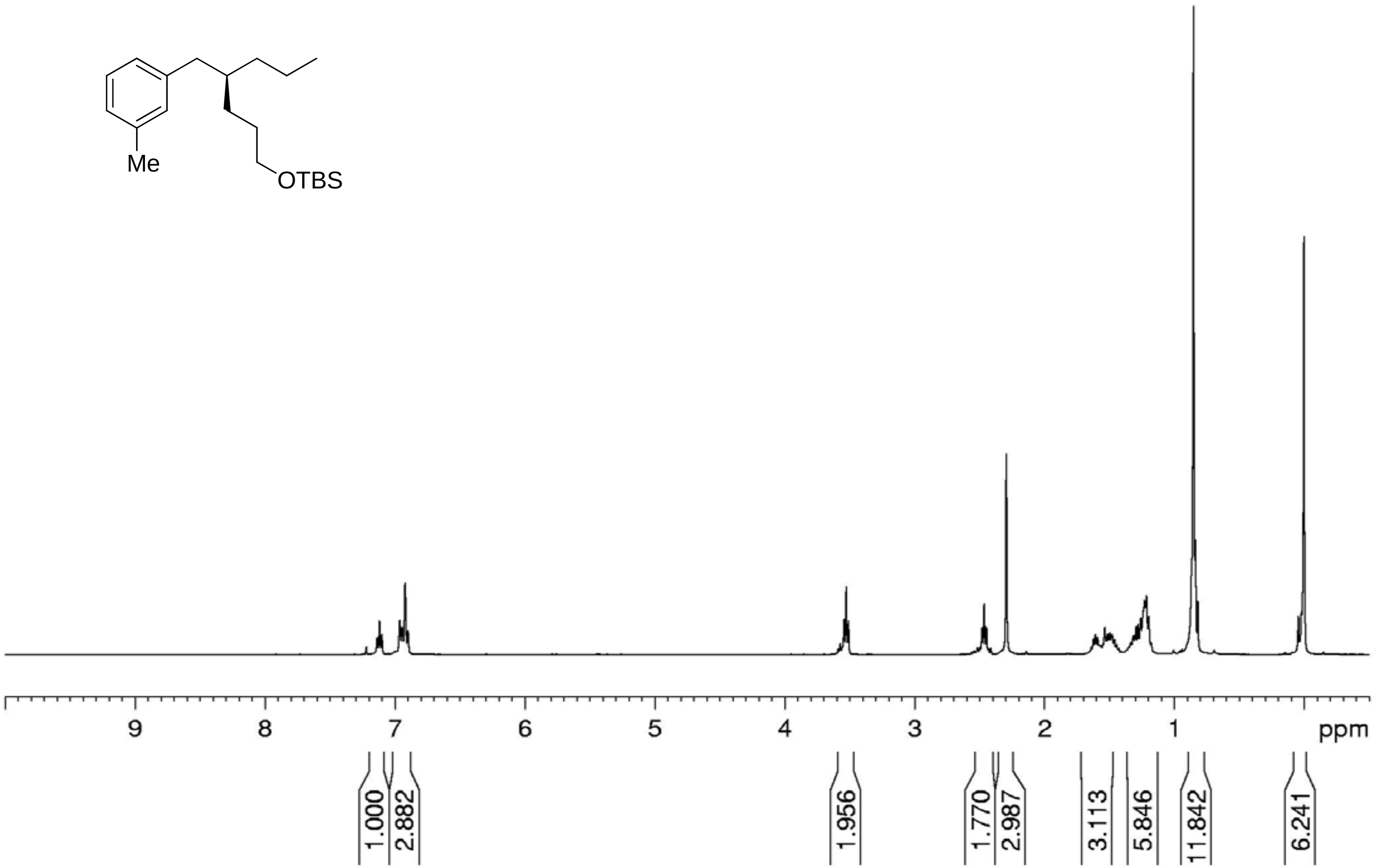
Me
OTBS
9
8
7
6
5
4
3
2
1
ppm
1.000
2.882
1.956
1.770
2.987
3.113
5.846
11.842
6.241

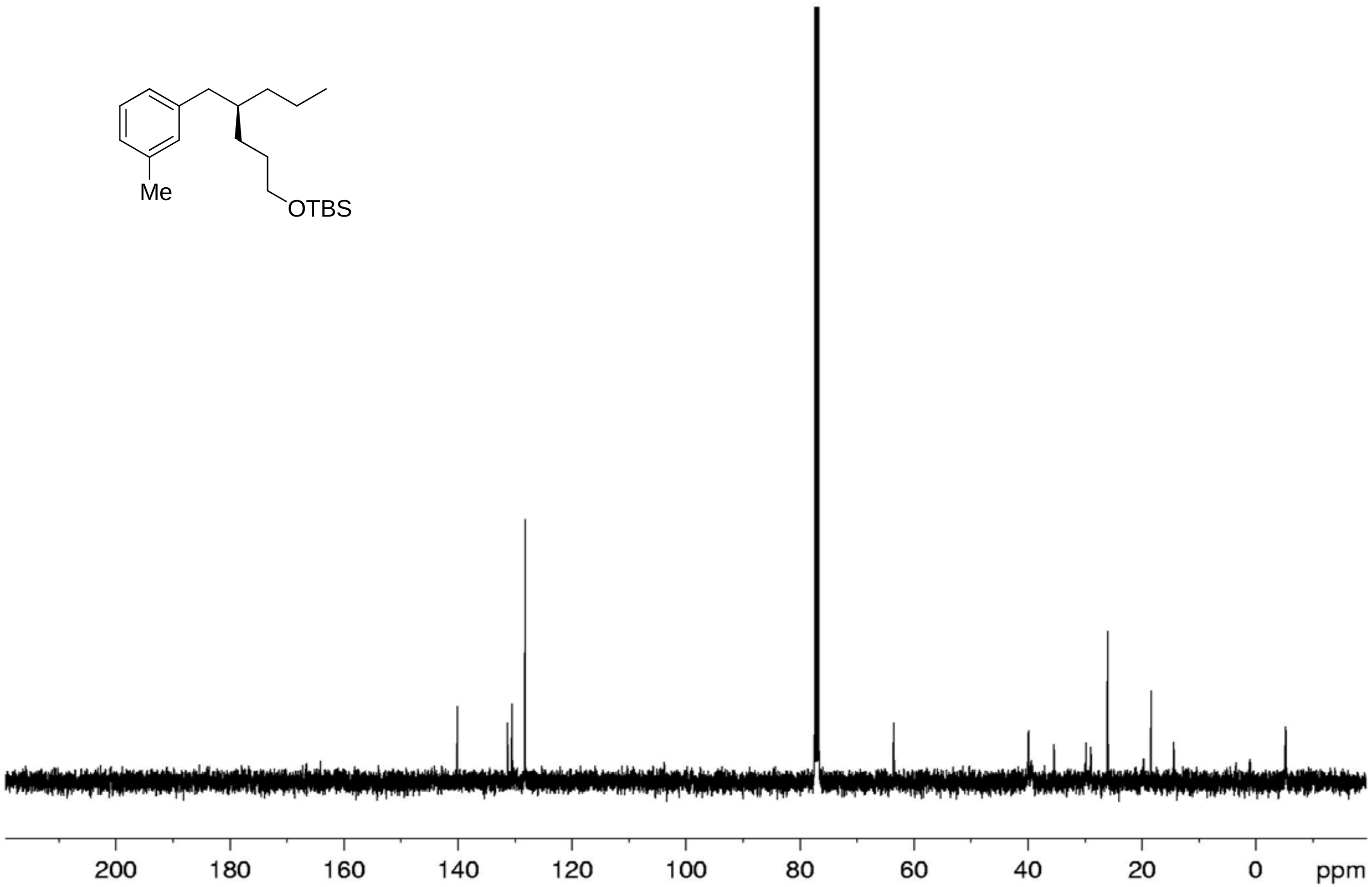
Me
OTBS
200
180
160
140
120
100
80
60
40
20
0
ppm

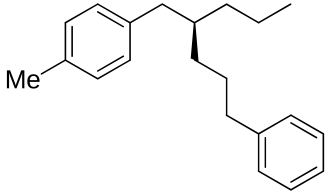
Me

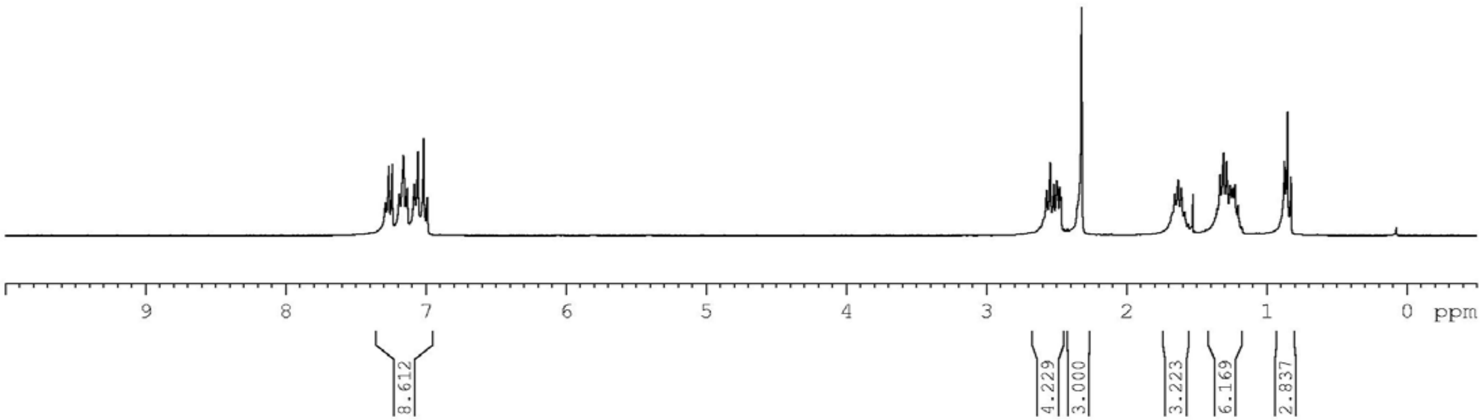
9
8
7
6
5
4
3
2
1
0 ppm
8.612
4.229
3.000
3.223
6.169
2.837

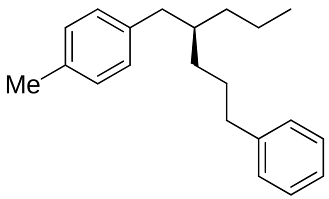
Me

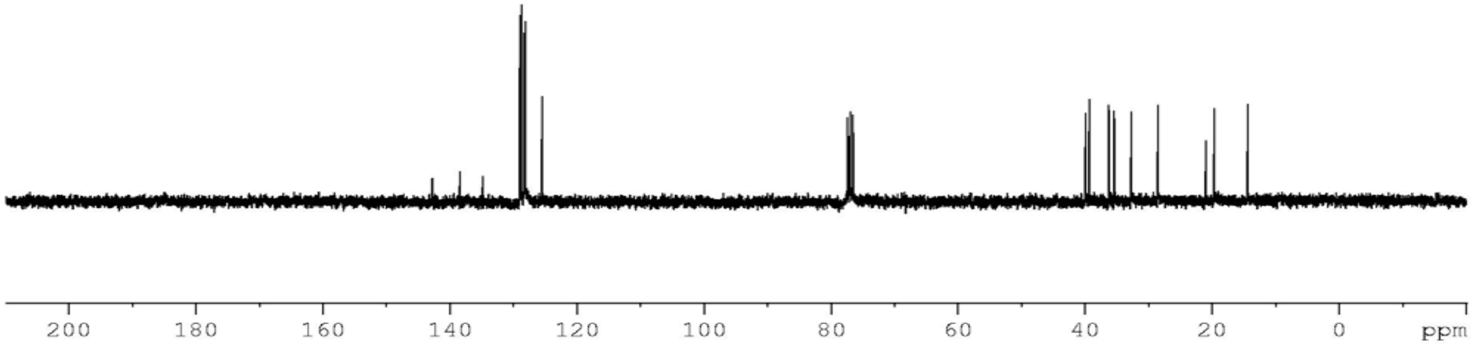
200
180
160
140
120
100
80
60
40
20
0
ppm

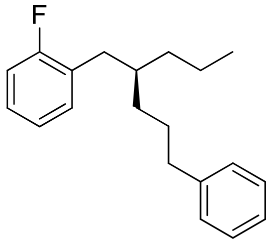
F

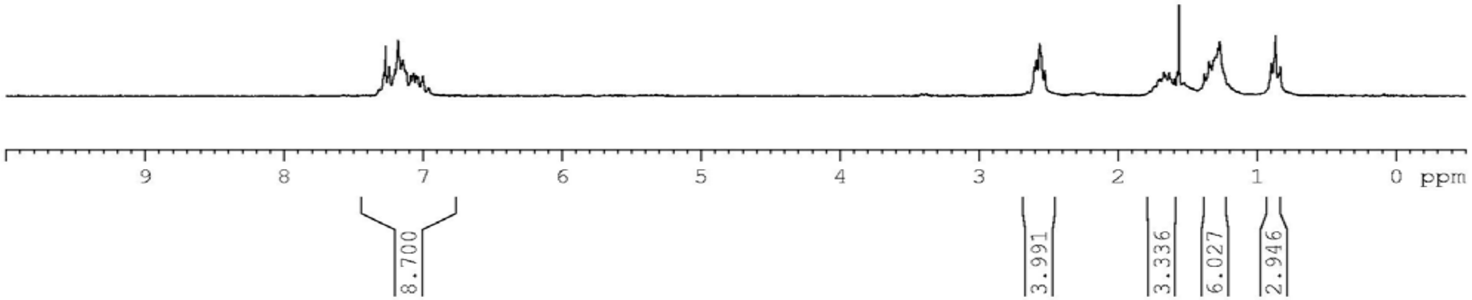
9
8
7
6
5
4
3
2
1
0 ppm
8.700
3.991
3.336
6.027
2.946

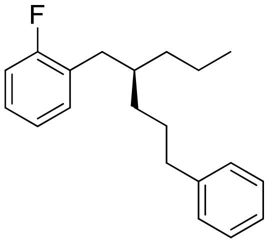
F

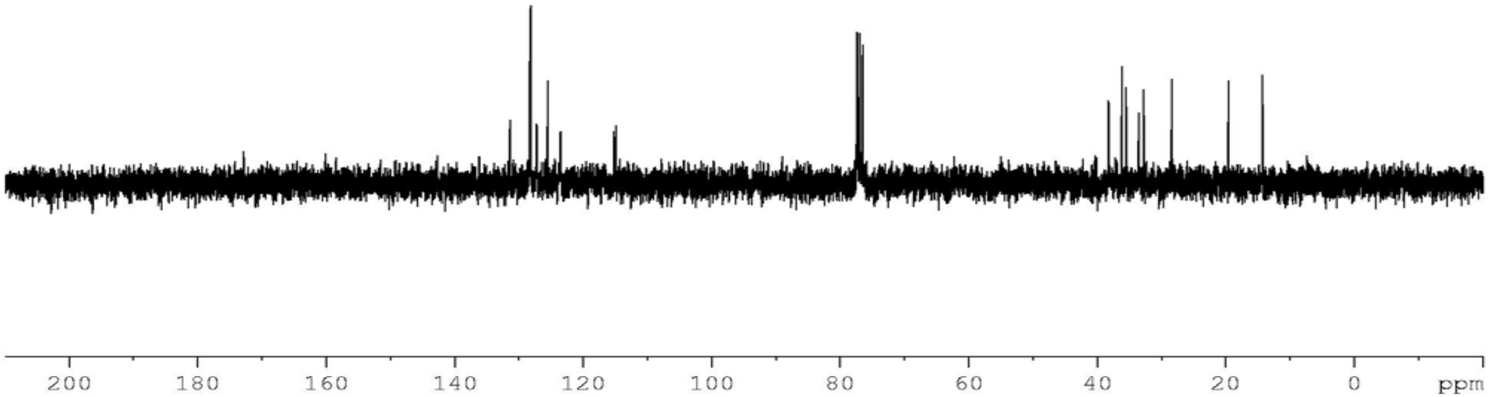
200
180
160
140
120
100
80
60
40
20
0
ppm

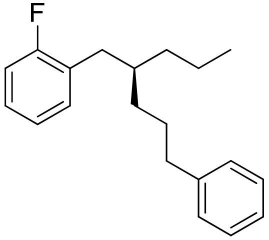

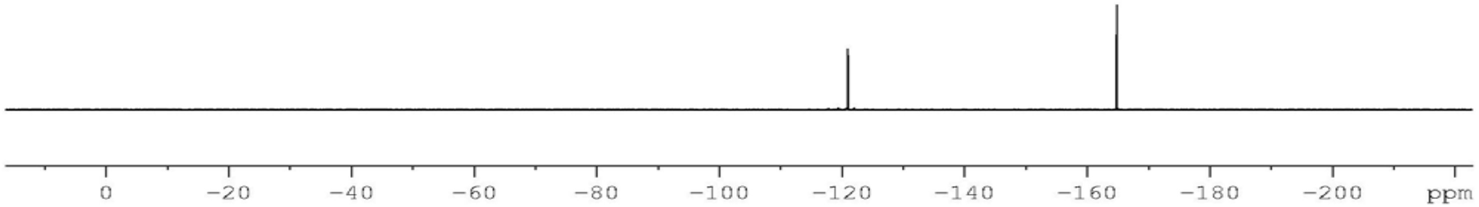
0
−20
−40
−60
−80
−100
−120
−140
−160
−180
−200
ppm

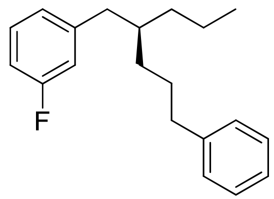
F

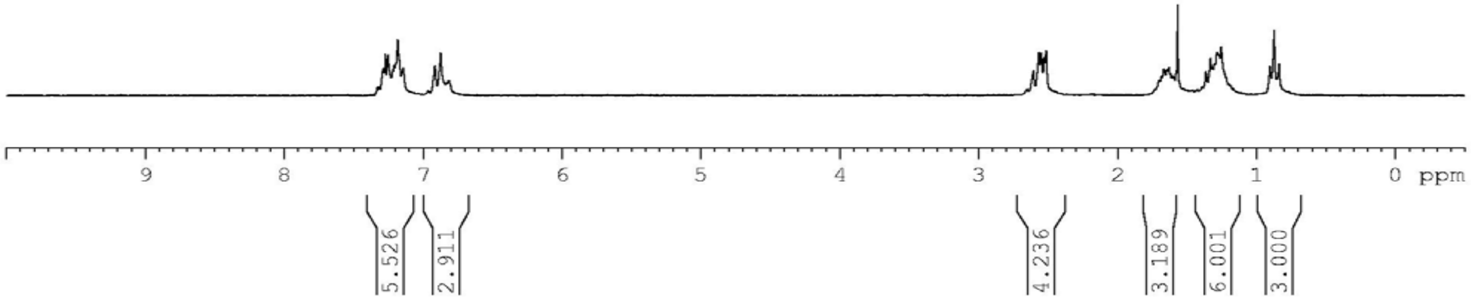
9
8
7
6
5
4
3
2
1
0 ppm
5.526
2.911
4.236
3.189
6.001
3.000

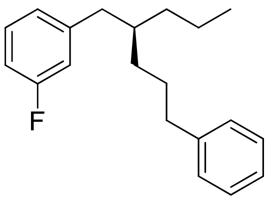
F

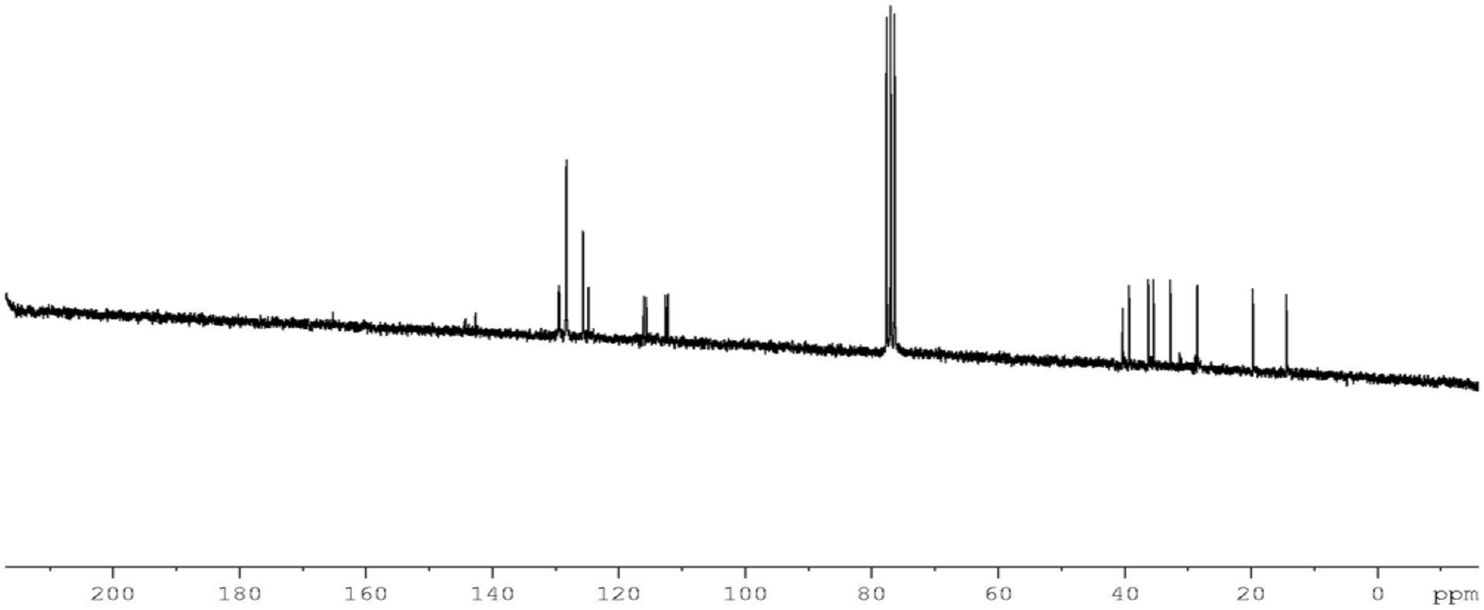
200
180
160
140
120
100
80
60
40
20
0
ppm

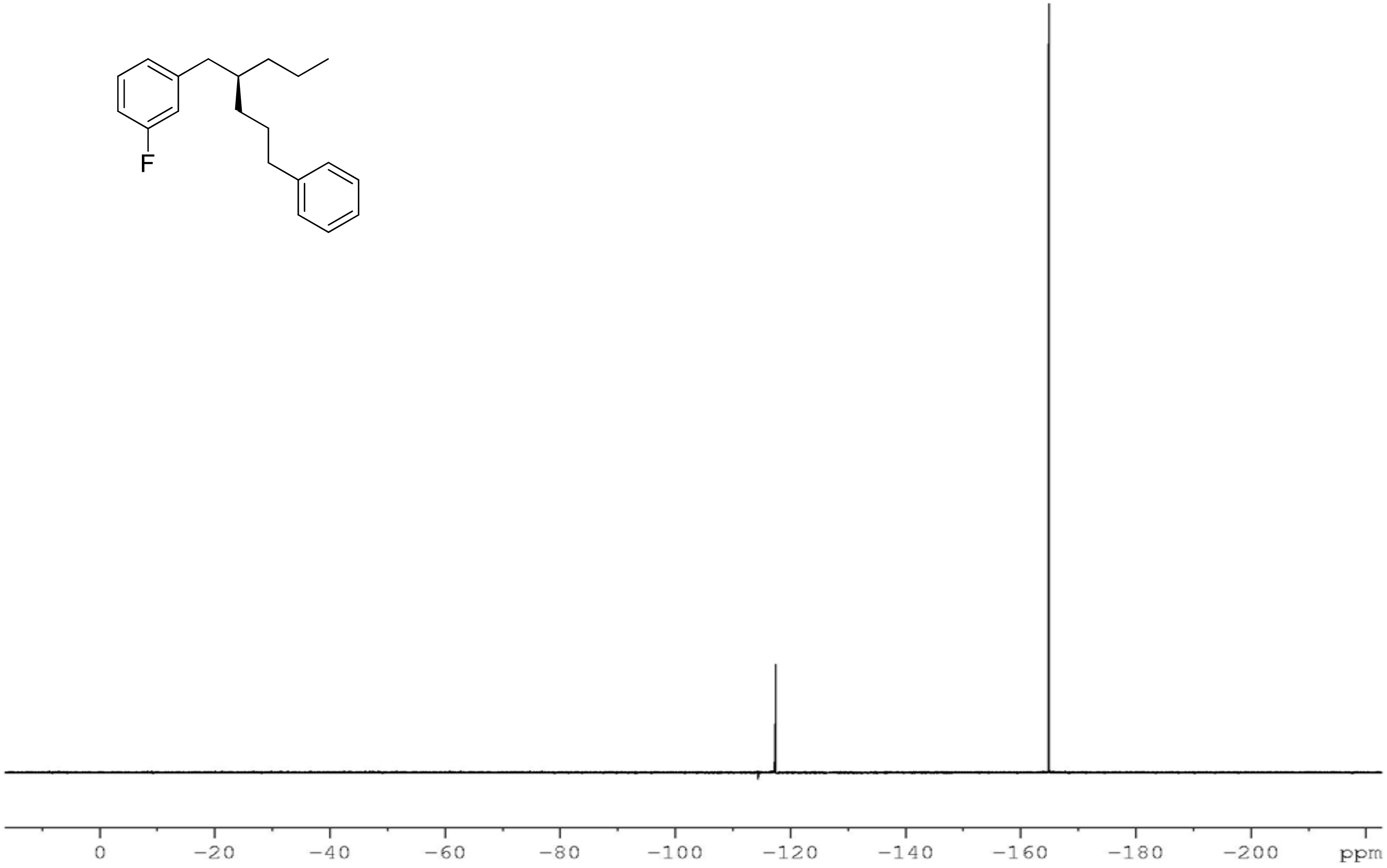

F
0
−20
−40
−60
−80
−100
−120
−140
−160
−180
−200
ppm

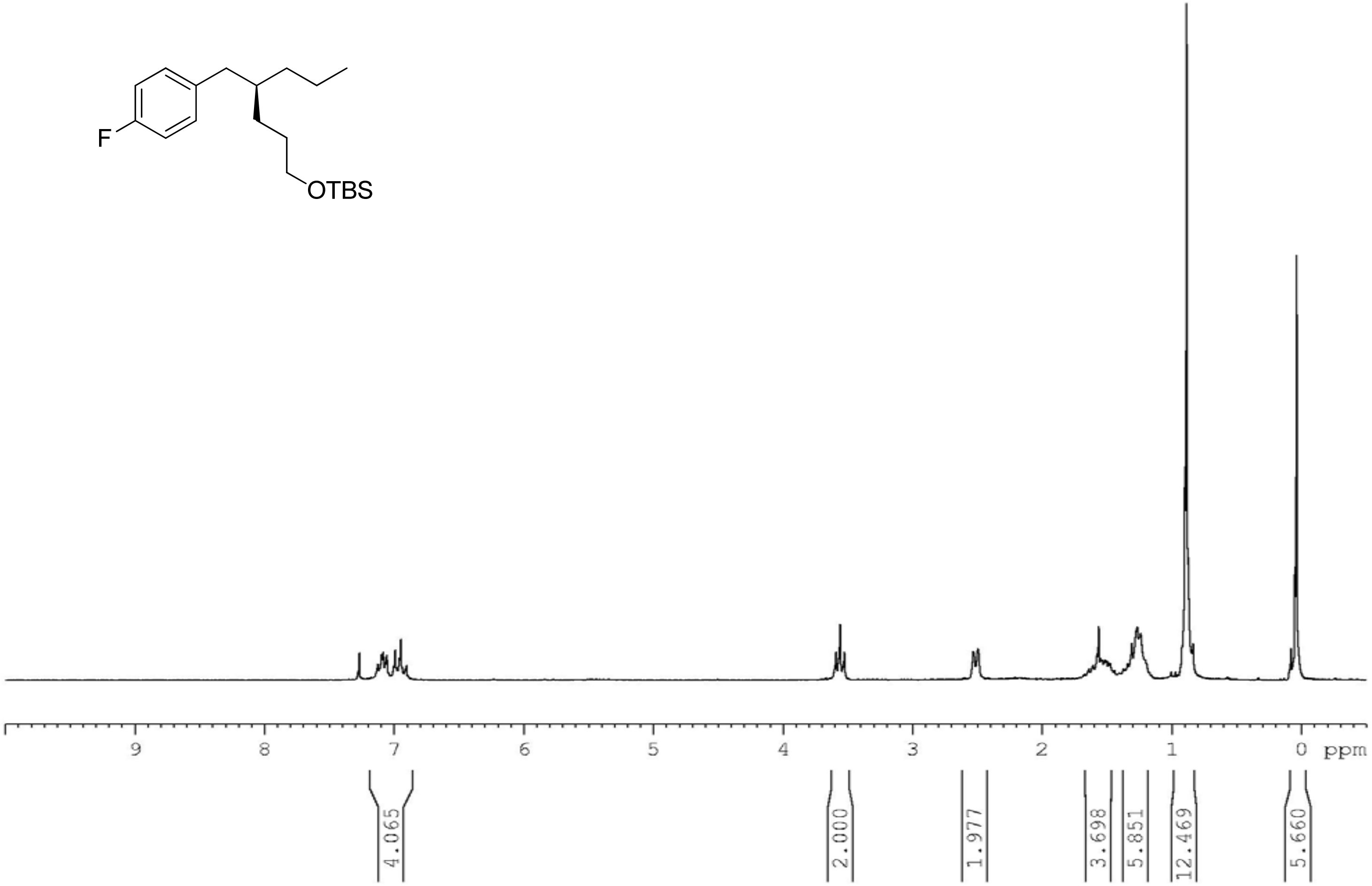
F
OTBS
9 8 7 6 5 4 3 2 1 0 ppm
4.065
2.000
1.977
3.698
5.851
12.469
5.660

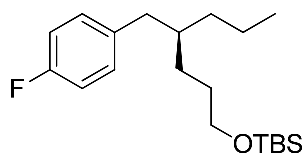
F
OTBS

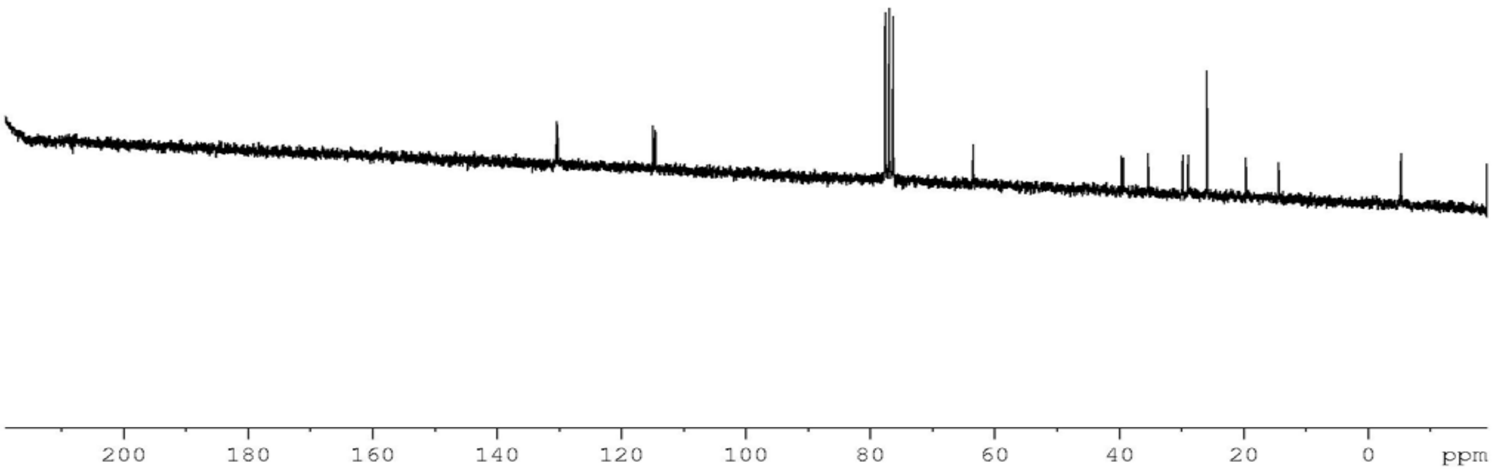
200
180
160
140
120
100
80
60
40
20
0
ppm

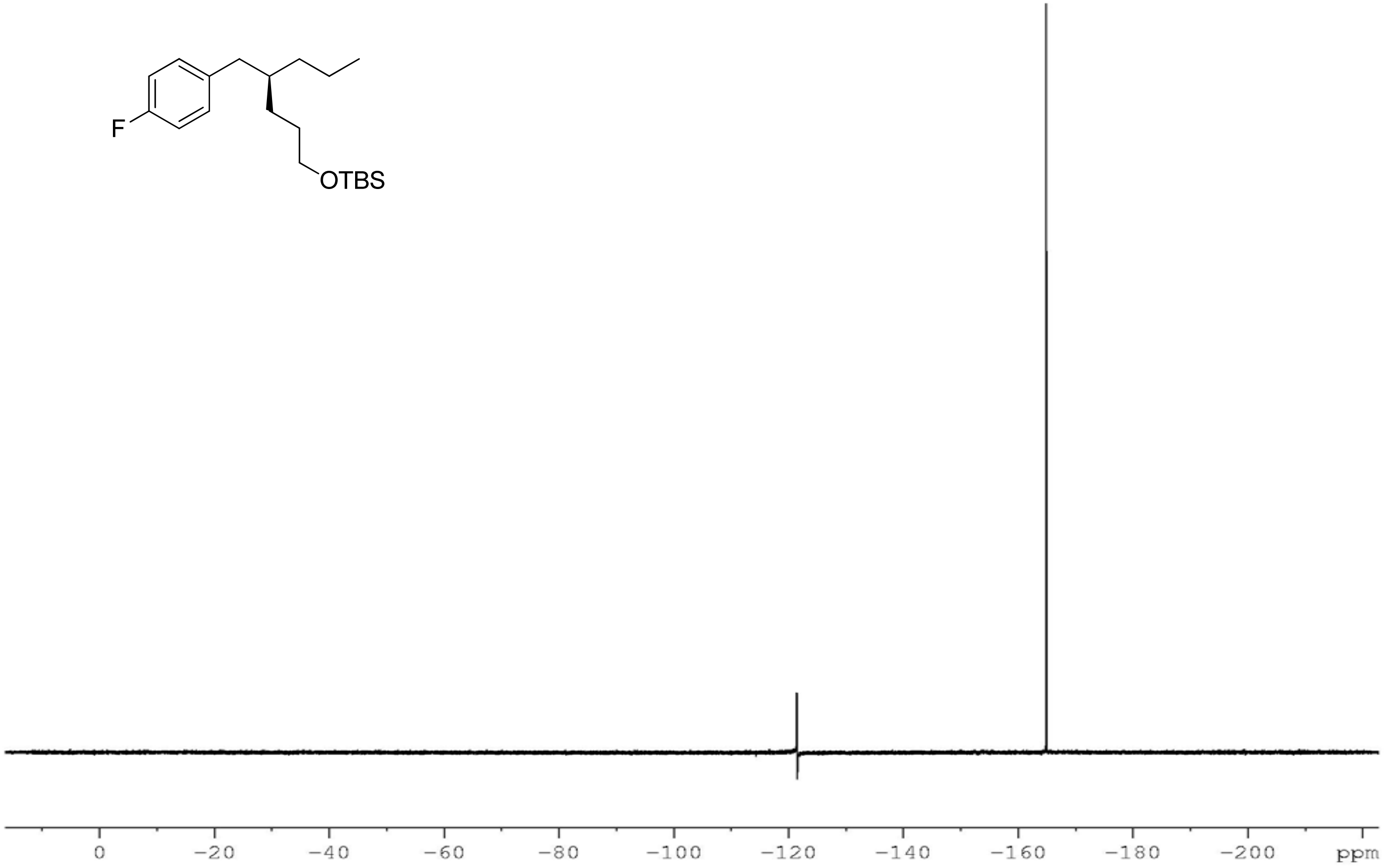
F
OTBS
0
−20
−40
−60
−80
−100
−120
−140
−160
−180
−200
ppm

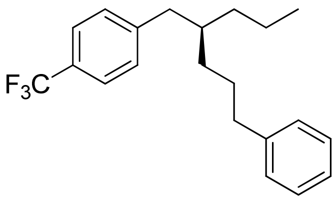
F3C

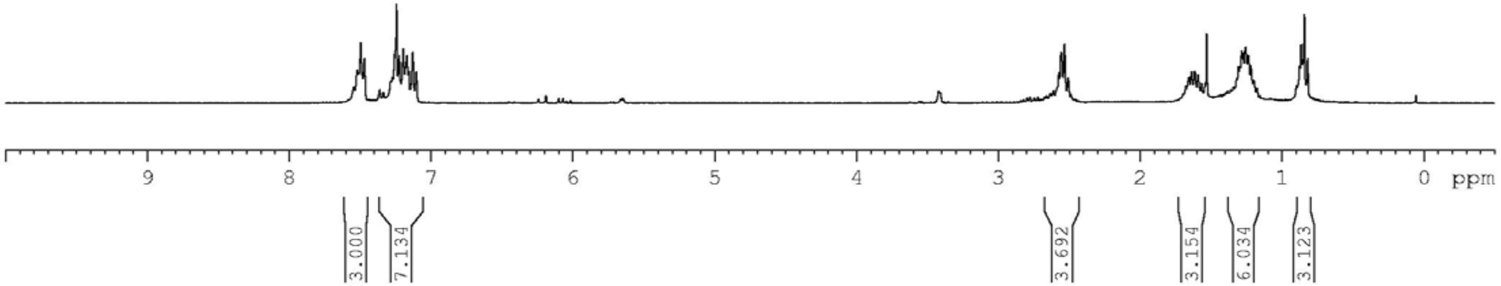
9
8
7
6
5
4
3
2
1
0
ppm
3.000
7.134
3.692
3.154
6.034
3.123

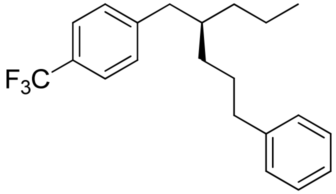
F3C

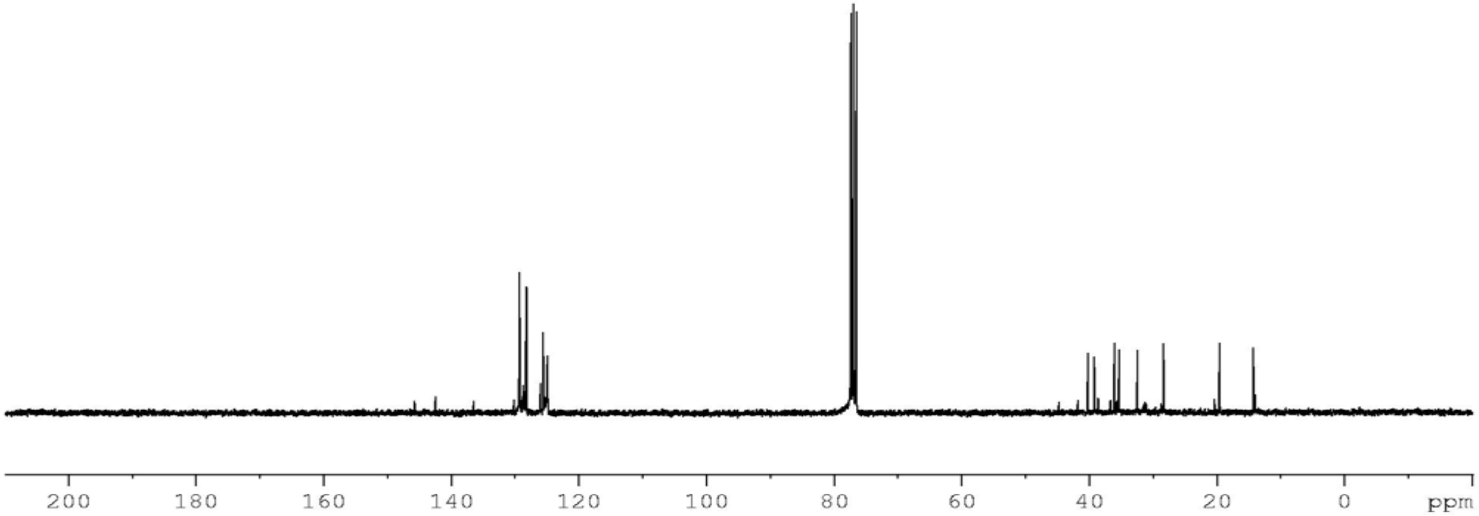
200
180
160
140
120
100
80
60
40
20
0
ppm

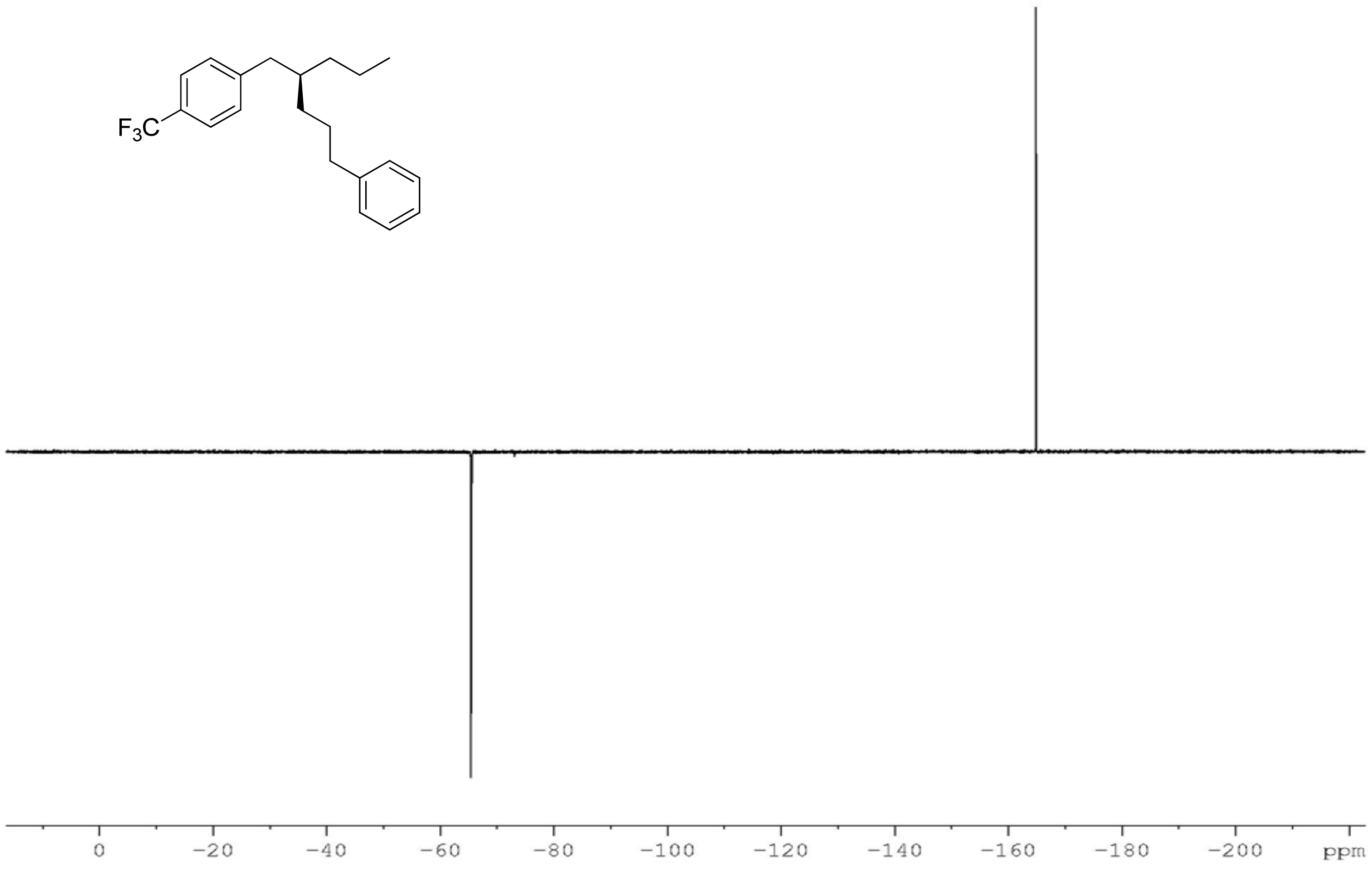
F3C
0
-20
-40
-60
-80
-100
-120
-140
-160
-180
-200
ppm

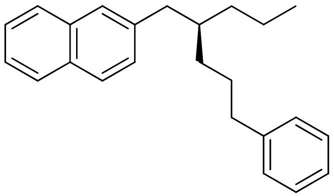

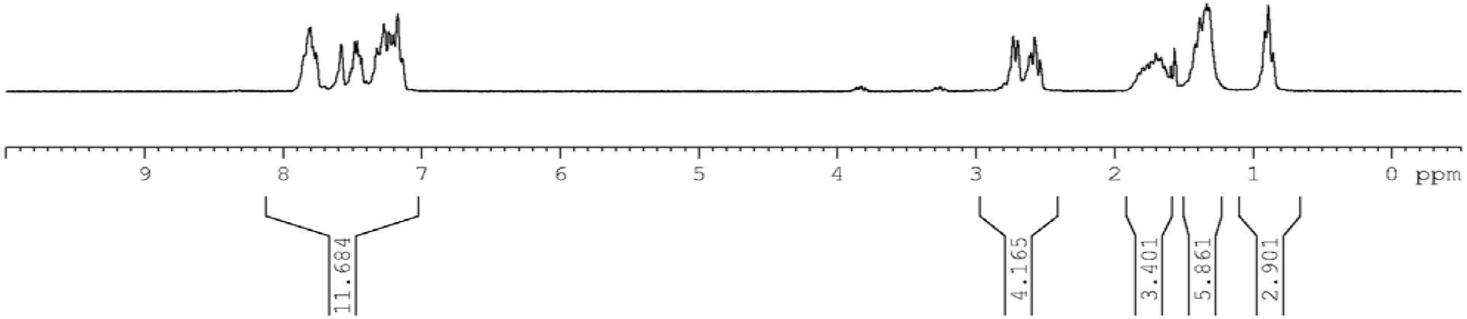
9
8
7
6
5
4
3
2
1
0 ppm
11.684
4.165
3.401
5.861
2.901

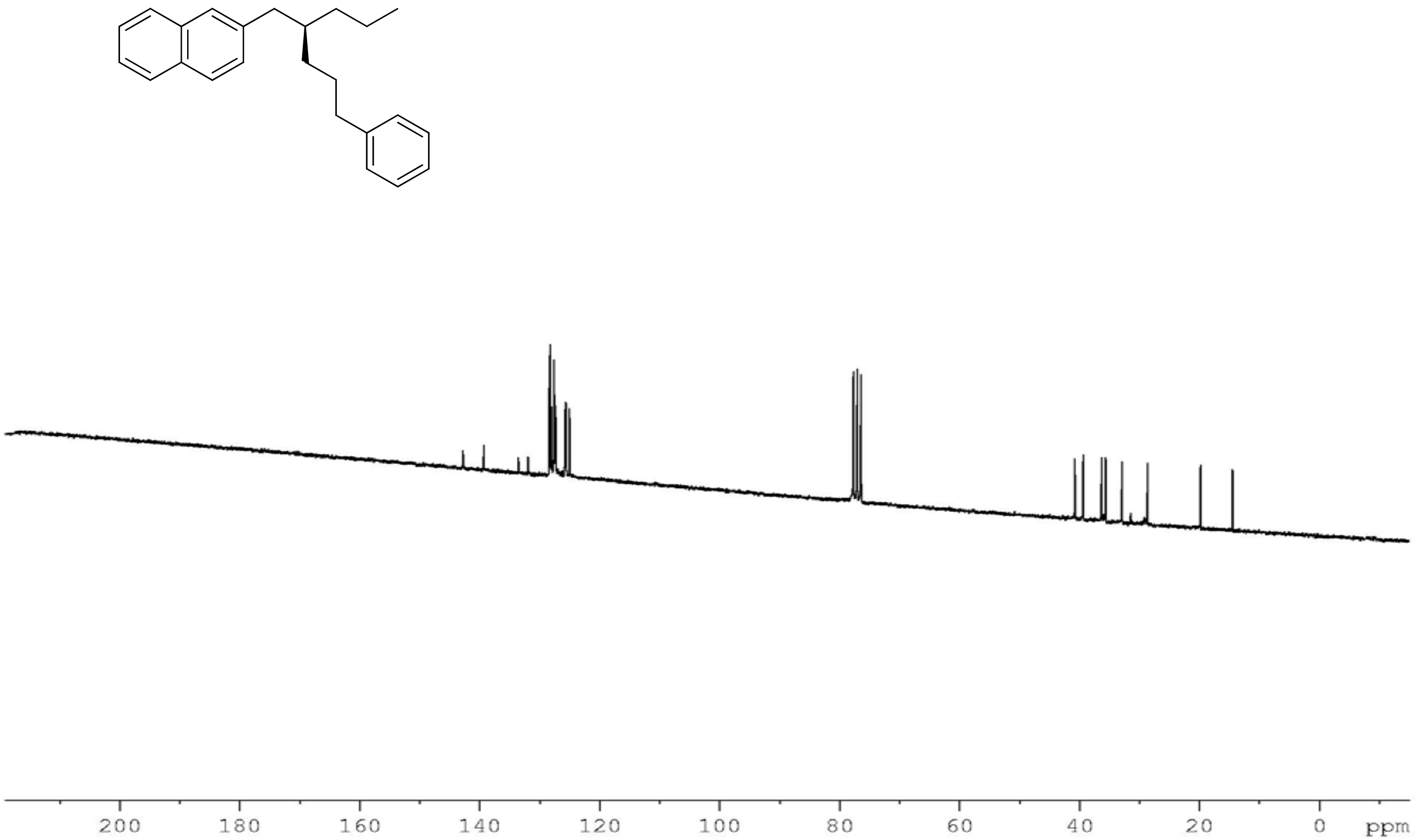
200
180
160
140
120
100
80
60
40
20
0
ppm

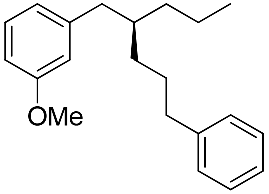
OMe

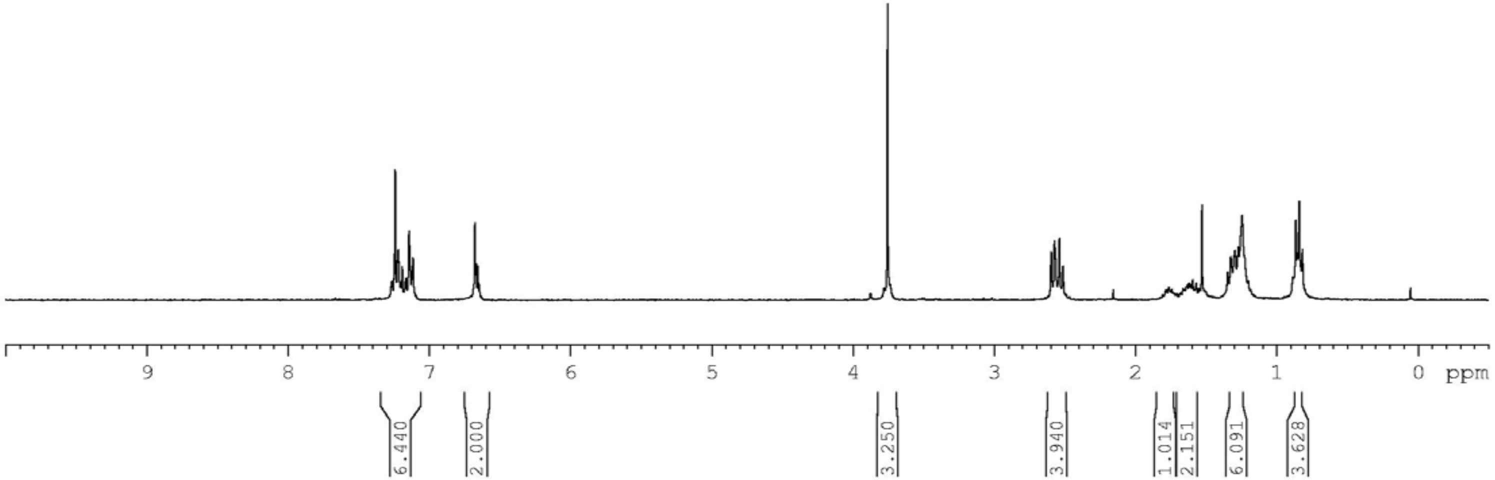
9
8
7
6
5
4
3
2
1
0 ppm
6.440
2.000
3.250
3.940
1.014
2.151
6.091
3.628

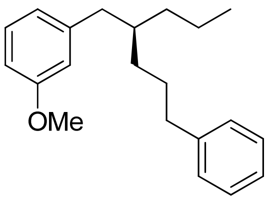
OMe

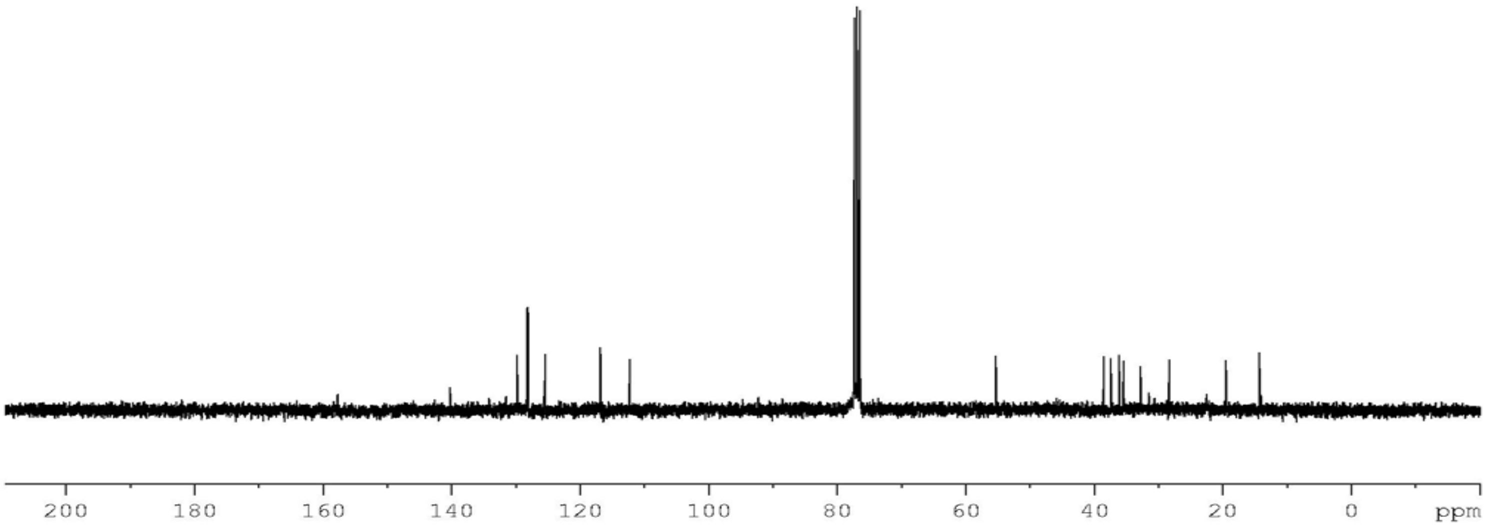
200
180
160
140
120
100
80
60
40
20
0
ppm

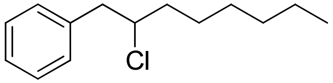
Cl

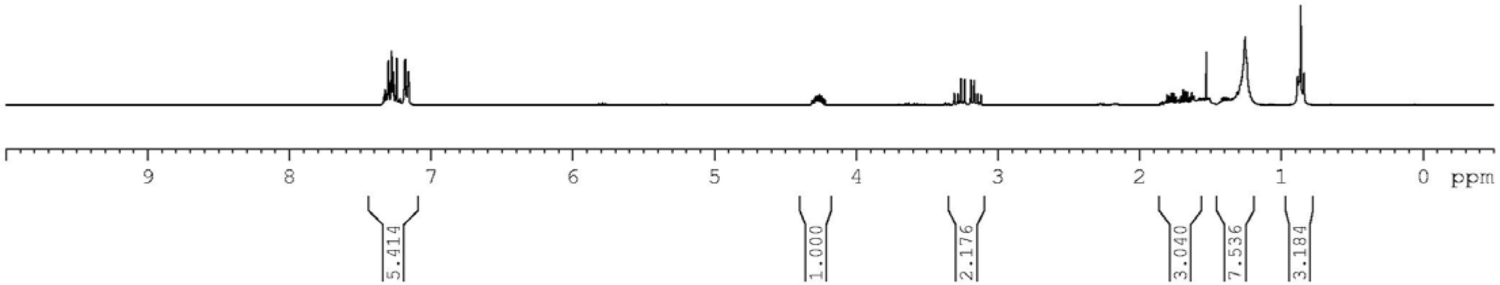
9
8
7
6
5
4
3
2
1
0
ppm
5.414
1.000
2.176
3.040
7.536
3.184

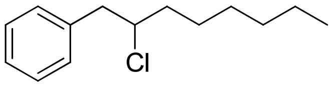
Cl

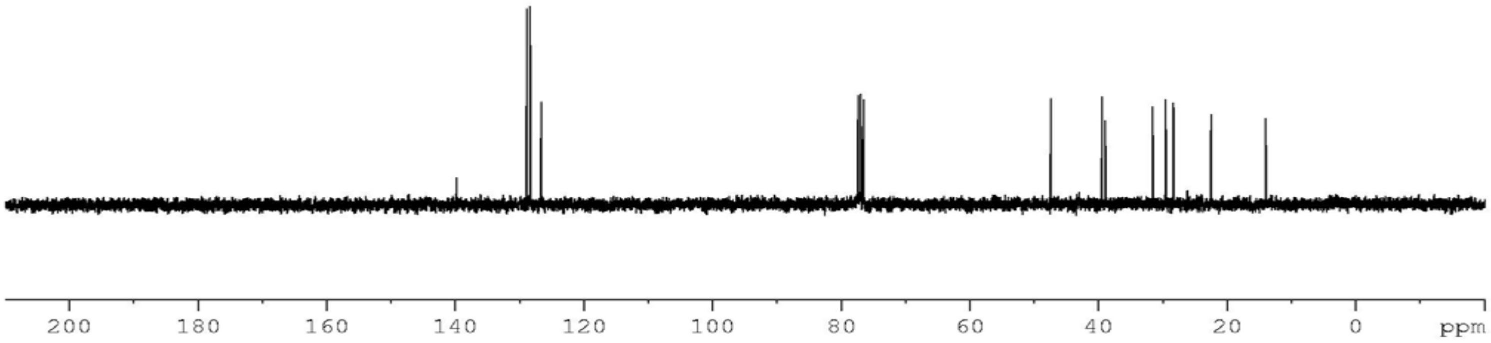
200
180
160
140
120
100
80
60
40
20
0
ppm
